# *Sophora flavescens*-*Astragalus mongholicus* herb pair in the progression of hepatitis, cirrhosis, and hepatocellular carcinoma: a possible mechanisms and relevant therapeutic substances

**DOI:** 10.3389/fphar.2024.1284752

**Published:** 2024-05-27

**Authors:** Xiao Yang, Chen Liang, Li Shao, Wenxuan Cui, Ruobing Ning, Fan Ke, Yue Wang, Peng Gao, Yidi Yin, Qing Li

**Affiliations:** ^1^ National and Local Joint Engineering Laboratory for Key Technology of Chinese Material Medica Quality Control, School of Pharmacy, Shenyang Pharmaceutical University, Shenyang, China; ^2^ Metabolomics Core Facility of RHLCCC, Feinberg School of Medicine, Northwestern University, Chicago, IL, United States

**Keywords:** hepatocellular carcinoma, *Sophora flavescens*-*Astragalus mongholicus* herb pair, Efficacy, mechanisms, therapeutic substances

## Abstract

**Background:**

Both *Sophora flavescens* (SF) and *Astragalus mongholicus* (AM) are known for their anti-inflammatory, antifibrotic, and anticancer activities. However, the efficacy, multi-target mechanisms, and therapeutic substances of SF-AM herb pair on the progression of hepatitis-cirrhosis-hepatocellular carcinoma hepatocellular carcinoma (HCC) remain unclear.

**Purpose:**

To investigate the efficacy, mechanisms, and potential therapeutic substances of SF-AM herb pair in the progression of hepatitis-cirrhosis-HCC.

**Methods:**

Firstly, diethylnitrosamine was used to establish the hepatitis-cirrhosis-HCC model. HE staining and non-targeted metabolomics were used to evaluate the efficacy of SF-AM herb pair. Subsequently, the absorbed components of SF-AM herb pair in the plasma of rats were determined through HPLC-Q-TOF-MS/MS analysis. Flow cytometry, Western blot, and qRT-PCR were then employed to assess CD4^+^ and CD8^+^ T lymphocytes, PI3K/Akt signaling pathway-related proteins, and their corresponding mRNAs. Simultaneously, the efficacy and mechanism of SF-AM herb pair on HCC were confirmed by *in vitro* experiments. Finally, Pearson correlation analysis was performed between pharmacodynamic indicators and *in vivo* components to identify the potential therapeutic substances of SF-AM herb pair.

**Results:**

SF-AM herb pair can alleviate the pathological damage and reverse metabolic abnormalities in hepatitis, cirrhosis, and HCC rats, particularly during the hepatitis and cirrhosis stages. Pharmacological researches have demonstrated that SF-AM herb pair can increase the proportion of CD8^+^ T lymphocytes, inhibit the expression of PI3K, Akt, p-Akt, NF-κB p65, NF-κB pp65, and Bcl-2, as well as increase the expression of IκBα, Bax, and cleaved caspase-3. These findings suggest that SF-AM herb pair has the ability to enhance immunity, anti-inflammation and promote apoptosis. Cell experiments have shown that SF-AM herb pair can inhibit the proliferation of HepG2 cell and regulate the PI3K/Akt signaling pathway. Moreover, 23 absorbed prototypical components and 53 metabolites of SF-AM herb pair were identified at different stages of HCC rats. Pearson correlation analysis revealed that matrine, cytisine, wogonoside, and isoastragaloside are potential therapeutic substances in SF-AM herb pair for the prevention and treatment of hepatitis, cirrhosis, and HCC.

**Conclusion:**

In summary, this study revealed the efficacy, mechanisms, and potential therapeutic substances of SF-AM herb pair in the hepatitis-cirrhosis-HCC axis and provided a reference for its clinical application.

## 1 Introduction

Liver cancer ranks third in death and sixth in incidence according to the latest global cancer data for 2020 released by the World Health Organization’s International Agency for Research on Cancer (Sung et al., 2021). Hepatocellular carcinoma (HCC) is the main type of primary liver cancer, accounting for approximately 75%–85% of cases (Feng et al., 2020). Chronic viral hepatitis, heavy alcohol use, genetic and metabolic disorders are the main risk factors for the development of HCC (Michael et al., 2015; Wangensteen and Chang, 2020). Exposure to risk factors such as HCV, HBV, alcohol, and diabetes can lead to inflammation in hepatic cell driven by Kupfer cells, dendritic cells, and hepatic stellate cells (Cubero, 2016). Chronic liver inflammation and the associated regenerative wound-healing response are closely linked to the development of fibrosis. Around 80% of HCC cases are associated with chronic hepatitis and cirrhosis, and a three-step process of hepatitis-cirrhosis-HCC is believed in the development of HCC.

Chronic inflammation is a risk factor for HCC and can damage hepatic epithelial cells, including hepatocytes and biliary epithelial cells. This damage induces substantial cell proliferation and reduces cell apoptosis due to the high regenerative capacity of liver. Further, chronic inflammation induces changes in the hepatic immune system, allowing cancer cells to easily evade immune surveillance (Yang et al., 2019). Generally, a variety of factors, including inflammation, immunity, and apoptosis acting together cause tumors to arise, and hepatitis-cirrhosis-HCC is considered as the progression process of HCC. Conventional chemotherapy drugs often contain only a single target, and their therapeutic effect in treating HCC remains unsatisfactory due to its toxicity. In contrast, traditional Chinese medicine (TCM) is characterized by multi-component and multi-target nature and has a long history of use in both the prevention and treatment of cancer in China and many other Asian regions. In recent years, TCM has gained popularity as a complementary and alternative medicine due to its excellent efficacy and safety with long-term medication (Wang et al., 2018).

From the perspective of TCM theory, *Sophora flavescens* Aiton [Fabaceae; Sophorae flavescentis radix] (SF) is cold in nature and bitter in taste and can clear heat and detoxify. *Astragalus mongholicus* Bunge [Fabaceae; Astragali radix] (AM) is mild in nature and sweet in taste and has the effect of invigorating “*qi*,” the substance that constitutes the human body and maintains human life activities. The combination of these two can achieve the effect of “strengthening the body and eliminating pathogens.” In modern medicine, SF is frequently employed in the treatment of viral inflammation and chronic liver illness because of its broad range of pharmacological effects, including anti-inflammatory, antifibrotic, antitumor, and immune regulatory effects (Shi et al., 2015; Sun et al., 2022). It has also been reported that AM has many pharmacological activities, including antiviral, antitumor, anti-inflammatory, and immunomodulatory actions (Li A. et al., 2020; Li S. et al., 2020). Additionally, both SF and AM have anticancer effects on H22 tumor-bearing mice (Zhang et al., 2020). Moreover, SF- and AM-related preparations, such as compound kushen injection (mainly composed of SF and Heterosmilax yunnanensis Gagnep./H. chinensis Wang) and KangAi injection (mainly composed of AM, Panax ginseng, and kushenin), are often used as adjuncts for cancer treatment clinically. Thus, the SF-AM herb pair has the potential to alleviate the hepatitis-cirrhosis-HCC axis. However, the efficacy and multi-target mechanisms of SF-AM herb pair in the development of hepatitis-cirrhosis-HCC are still unclear.

To explore the efficacy and multi-target mechanisms of SF-AM herb pair on diethylnitrosamine (DEN)-induced hepatitis-cirrhosis-HCC rats, the workflow of this study was mainly summarized as follows: 1) Evaluate the efficacy of SF-AM herb pair in hepatitis-cirrhosis-HCC rats by histopathological examination and non-targeted metabolomics; 2) Analyze the absorption and metabolism of SF-AM herb pair in the plasma of hepatitis-cirrhosis-HCC rats by HPLC-Q-TOF-MS/MS; 3) Investigate the pharmacological mechanisms of SF-AM herb pair on hepatitis-cirrhosis-HCC rats by determining CD4^+^ and CD8^+^ T lymphocyte subsets, PI3K/Akt signaling pathway-related proteins, and their corresponding mRNAs; 4) Explore the potential therapeutic substances of SF-AM herb pair in hepatitis-cirrhosis-HCC by correlating the drug-recalled endogenous metabolites with the absorbed prototypical components and verifying them by molecular docking experiments.

## 2 Materials and methods

### 2.1 Reagents and chemicals

*Sophora flavescens* Aiton [Fabaceae; Sophorae flavescentis radix] and *Astragalus mongholicus* Bunge [Fabaceae; Astragali radix] were obtained from Guoda Pharmacy (Shenyang, China). The origin *Sophora flavescens* is Hebei province and the batch number is 21040601; the origin of *Astragalus mongholicus* is Gansu province and the batch number is 21022001. The botanical and morphological authentication were conducted by Professor Dong Wang (School of Chinese Material Medica, Shenyang Pharmaceutical University, Shenyang, China).

The extraction process of SF-AM herb pair extract is as follows: Appropriate amount of SF and AM radix (3:1, w/w) were accurately weighted and boiled with 8 times the amount of water for 1 h. After filtration, the residue was collected, and the extraction was repeated with six times the amount of water. Subsequently, the two filtrates were combined and concentrated under vacuum to a final concentration of 1.0 g/mL (equal to crude plant) at 65°C. Single chemical fingerprinting method with three detection wavelengths was conduct to evaluate the chemical components of SF-AM herb pair extract, and the details were provided in Supplementary Material S1.1 and Supplementary Figure S1. Additionally, the quality consistency of SF-AM herb pair extract extracted at different times was evaluated by HPLC fingerprint chromatogram, and the similarity is greater than 0.8, as shown in Supplementary Figure S2.

PE Mouse Anti-Rat CD8a antibody was obtained from BD Biosciences (Lake Franklin, New Jersey, United States). FITC anti-rat CD4, and Anti-CD3 FITC antibody were bought from BioLegend (San Diego, California, United States). The reference standards (purity ≥98%) for component identification and internal standards are listed in Supplementary Table S1. DEN was obtained from Sigma-Aldrich (MO, United States). 5-Fluorouracil (5-Fu) and KangAi injection were purchased from Shanghai Xudong Haipu Pharmaceutical Co., Ltd. and Changbaishan Pharmaceutical Co., Ltd., respectively. Methanol, acetonitrile and formic acid were from Fisher Scientific (Fair Lawn, NJ, United States). Deionized water was bought from Wahaha Co. Ltd. (Hangzhou, China).

### 2.2 Animals

Wistar rats (Male, 200 ± 20 g, License NO. SCXK (LIAO) 2020-0001) were used. The Animal Ethical Committee of Changsheng Biotechnology authorized the protocol (CSE202104003), and all animal tests were done in compliance with the NIH criteria. Rats were housed in groups at indoor temperature with 40%–60% humidity for 7 days prior to the trials. Water and food were freely available.

**FIGURE 1:**
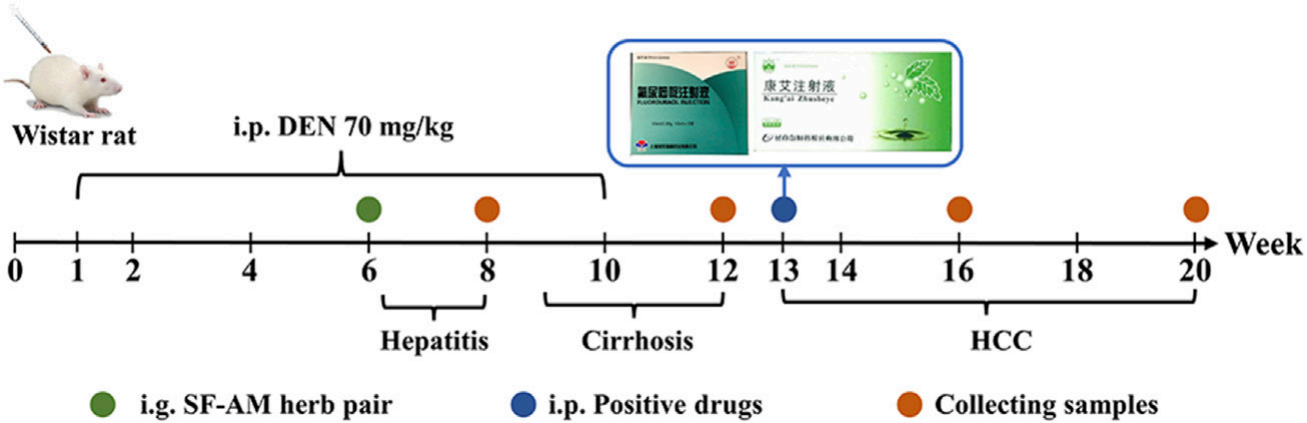
Experimental schedule.

156 rats were divided into five groups: control group (Control, *n* = 24), model group (Model, *n* = 60), low-dose group (Low, *n* = 24, 2.5 g/kg), middle-dose group (Middle, *n* = 24, 5.0 g/kg) and high dose group (High, *n* = 24, 10.0 g/kg). Except for the control group, all rats were given DEN (70 mg/kg, ip. once a week, Sigma, United States) for 10 weeks, and the rats in the control group were given equal amount of 0.9% NaCl (ip.). The rats in herb pair groups were given SF-AM herb pair extract (ig. once a day) at week 7 until week 20. By the week of 13, the previous model group was further divided into the following four groups: model group (*n* = 12), 5-Fu group (ip. 20 mg/kg, once a week, *n* = 12), KangAi group (ip., 4.5 mL/kg, once a day, *n* = 12) and Union group (5-Fu+Middle-dose group, *n* = 12). The experiment schedule was given in Figure 1. At week 8, 12, 16, and 20, the rats were given a 12-h fast, and blood was taken to make plasma for usage. In the middle-dose group, the plasma 1 h after administration was collected for *in vivo* components analysis. Rats in each group were then sacrificed, partial fresh liver tissues were taken to determine T lymphocyte subsets and other part were soaked in 4% paraformaldehyde for histological analysis. The leftover liver tissues were kept at −80°C for later use.

### 2.3 Histopathological examination

Rat livers were sliced into 4-mm-thick slices after being sectioned into the necessary blocks, fixed in 4% paraformaldehyde for 48 h, dried until translucent, and embedded in wax. Then, these slices were then stained with hematoxylin-eosin (HE) for histopathological analysis under light microscopy.

### 2.4 Non-targeted metabolomics analysis

The experiments were performed on an Agilent 1260 infinity liquid chromatography system (Agilent, Santa Clara, CA, United States) in tandem with a triple TOF 5600+ mass spectrometer (Sciex, Redwood City, CA, United States) via an electrospray ionization interface. For separation, an InfinityLab Poroshell 120 SB-AQ column (4.6 mm × 100 mm, 2.7 μm) was used. The mobile phase included water (A) and acetonitrile (B), both of them containing 0.1% formic acid. Gradient elution program was as follows: 0.01–5.00 min from 2% to 10% B, 5.00–10.00 min from 10% to 40% B, 10.00–20.00 min 40% to 60% B, 20.00–30.00 min 60% to 90% B, 30.00–36.00 min 90% to 2% B and hold on 2% B for 5 min at 0.5 mL/min. Non-targeted MS/MS acquisition was conducted by SWATH (Q1 *m/z*: 50–1,000). swathTUNER was used to optimize the size of Q1 windows. Experiment window design was provided in Supplementary Tables S2, S3. For sample preparation, 100 μL plasma, 10 μL methanol, 10 μL internal standards (containing 10 μg/ml L-2-chlorophenylalanine, d_4_-cortisol, heptadecanoic acid, d_8_-Arachidonic acid) and 300 μL methanol-acetonitrile (1:1, *v/v*) were added into a 1.5 mL Eppendorf tube, vortexed for 30 s, and centrifugated at 12,000 rpm for 5 min (4°C). The supernatants were transferred and dried under 35°C, then 70 μL of 75% methanol solution was added, vortexed for 30 s, ultrasound for 5 min, and centrifugated (12,000 rpm, 4°C, 10 min). Finally, 4 μL of supernatants were used for analysis.

The raw data were converted to Analysis Base Files via the ABF converter firstly and then MS-DIAL software (parameters were listed in Supplementary Table S4) was employed to analyze the data. OPLS-DA score plots were applied to visualize the separation among different groups. Differential endogenous metabolites were screened out by combining *p* < 0.05 in the Student’s t-test and VIP>1 in OPLS-DA. Finally, the MetaboAnalyst platform 5.0 (https://www.metaboanalyst.ca/) was used for pathway enrichment analysis.

### 2.5 *In vivo* components of SF-AM herb pair in the plasma of hepatitis-cirrhosis-HCC rats

A total of 20 μL of internal standards (containing 10 μg/mL nuciferine, galuteolin and Ginsenoside Re) and 600 μL of methanol were added into 200 μL medicated plasma, vortexed for 30 s, and centrifugated at 12,000 rpm for 5 min (4°C). The supernatants were transferred and dried under 35°C. Then, 70 μL of 75% methanol solution was added, vortexed for 30 s, ultrasound for 5 min, and centrifugated (12,000 rpm, 4°C, 10 min). And they were then examined by HPLC-Q-TOF-MS/MS system (Supplementary Material S1.2).

### 2.6 Flow cytometry analysis

A 6-well plate containing fresh liver tissue was filled and ground. The cells were then extracted with the aid of a lymphocyte separation solution, labeled with the aforementioned antibodies, and analyzed by flow cytometry (ACEA NovoCyte, United States), the data was analyzed by NovoExpress (Agilent, Santa Clara, CA, United States).

### 2.7 Western blot analysis

Liver tissue was cut and lysed in RIPA buffer containing 1% PMSF, ultrasonicated on ice (150 W, 120 s), and centrifuged (12,000 rpm, 4°C, 15 min). The protein concentration was determined by a BCA determination kit (Thermo Scientific Co., Ltd.). Forty-five micrograms of total protein were separated by 10% or 12% SDS-PAGE and then transferred to a PVDF membrane. The membrane was blocked by rapid sealing fluid for 10 min (Genefist, Nanjing, China), and then incubated for 12 h with PI3K (1:1,000, 4249T, CST), Akt (1:1,000, 4691T, CST), p-Akt (phospho T308, 1:1,000, ab278565, abcam), NF-κB p65 (1:1,000, 8242T, CST), NF-κB pp65 (phosphorylation site Ser536, 1:1,000, 3033T, CST), IκBα (1:1,000, 4812T, CST), Bcl-2 (1:500, ab196495, Abcam), Bax (1:1,000, 2772T, CST), cleaved caspase-3 (1:1,000, 9661T, CST), β-actin (1:1,000, 4970T, CST) at 4°C. After washing (5 min × 3), the membrane was incubated with a secondary antibody (1:10,000, bs-0295G-HRP, Bioss, Beijing, China) for 1 h. Following washing, the ECL chemiluminescence solution was uniformly applied and the Tanon gel imaging system (Tanon-5200Multi, Tanon, Shanghai, China) was used for detection. Then the strip was quantified by ImageJ (NIH, Bethesda, MD, United States). As a measure of protein expression in each membrane, β-actin was computed.

### 2.8 Quantitative real-time PCR (qRT-PCR) analysis

Total RNA was extracted from the liver tissues using TransZol Up (Transgen, China). NanoDrop spectrophotometer (NanoDrop one, Thermo Scientific, United States) was used to analyze the RNA yields and purity. Total RNA (1 μg/μL) transcription was performed using an *in vitro* transcription kit (RevertAid Master Mix, Themo Scientific, United States). The qRT-PCR reactions (20 μL) consisted of 10 μL iTaq Universal SYBR Green Supermix (2×), 0.8 μL PCR forward primer (10 μM), 0.8 μL PCR reverse primer (10 μM), 1 μL cDNA, and 7.4 μL water (nuclease-free). A typical qRT-PCR procedure was set according to the manufacturer’s instructions. PCR was carried out in triplicate, and the primer sequences were listed in Table 1.

**TABLE 1:** The sequence of the primers for PI3K, Akt, NF-κB p65, Bcl-2, Bax, caspase-3, and GAPDH.

Genes		Primer sequence (5′>3′)
PI3K	F	GCT​GTT​GAT​AGA​CCA​CCG​CTT​CC
	R	TGC​CCT​GTT​CCT​CTG​CCT​TCC
Akt	F	TCA​CCT​CTG​AGA​CCG​ACA​CC
	R	ACT​GGC​TGA​GTA​GGA​GAA​CTG​G
NF κB p65	F	ACT​CTT​GAG​ACC​CTG​CTT​CC
	R	TGC​TTT​GGA​TCA​AGG​TGT​GC
Bcl-2	F	GGG​TCA​TGT​GTG​TGG​AGA​G
	R	AGC​CAG​GAG​AAA​TCA​AAC​AG
Bax	F	GAA​CTG​GAC​AAC​AAC​ATG​GA
	R	GCA​AAG​TAG​AAA​AGG​GCA​AC
Caspase-3	F	ACG​GGA​CTT​GGA​AAG​CAT​C
	R	TAA​GGA​AGC​CTG​GAG​CAC​AG
GAPDH	F	AGA​CAG​CCG​CAT​CTT​CTT​GT
	R	CTT​GCC​GTG​GGT​AGA​GTC​AT

qRT-PCR was conducted by CFX-96 Optics Module (Bio-RAD, Singapore). Data analysis was ascertained using the 2^−ΔΔCt^ method, and normalized by GAPDH. The relative integrated intensity in relation to the control group was used to express the results.

### 2.9 Cell culture and treatments

The human HCC cell line HepG2 and human hepatocyte cell line L02 were purchased from the Meilunbio Co., Ltd. (Dalian, China) and FENGHUISHENGWU Co., Ltd. (Changsha, China), respectively. HepG2 cells were grown in MEM medium (HyClone, UT, United States) with 10% fetal bovine serum (Gibco, NY, United States) and 1% penicillin-streptomycin. L02 cells were grown in RPMI-1640 medium (HyClone, UT, United States) with 20% fetal bovine serum (Gibco, NY, United States) and 1% penicillin-streptomycin. Cell lines were kept at 37°C in a cell culture incubator (Model 3111, Thermo, CA, United States) with 5% CO2.

For cell viability tests, cells were seeded in 96-well culture plates (Thermo Scientific, CA, United States) at a density of 5 × 10^3^ cells per well and then treated with various concentrations of SF-AM herb pair extract (0, 5, 10, 20, 30, 40, and 50 mg/mL) for 24, 36, and 48 h. Cell viability was assessed following incubation using a standard methyl thiazolyl tetrazolium assay. An Infinite 200 Pro fluorescence spectrometer (Tecan, Salzburg, Austria) was used to measure fluorescence intensities.

For *in vitro* mechanism verification, HepG2 cells were seeded in 100-mm cell culture dishes at a density of 3 × 10^6^ and incubated for 24 h. Then, HepG2 cells were divided into the HepG2 group, HepG2+LY294002 (PI3K inhibitor, 20 μM), HepG2+SF-AM-L, HepG2+SF-AM-H, and HepG2+LY294002+SF-AM-H groups. The low- and high-dose concentrations of SF-AM herb pair extract were 10 and 30 mg/mL, respectively. These cells were cultured with the corresponding drug for 24 h and then washed and lysed. The levels of PI3K, Akt, p-Akt, NF-κB p65, NF-κB pp65, IκBα, Bcl-2, Bax, and cleaved caspase-3 protein in these groups were determined in accordance with the “Section 2.7 Western blot analysis” method.

### 2.10 Molecular dockings

SDF 2D structures of bioactive components were obtained from Pubchem (https://pubchem.ncbi.nlm.nih.gov/) and then transformed into mol2 structure by chem3D. The protein files of PI3K (PDB: 4UWH), Akt (PDB: 4GV1), NF-κB p65 (PDB: 6NV2), and Bcl-2 (PDB: 6O0K) were acquired from the RCSB Protein Data Bank (https://www.rcsb.org/). These models were then submitted to the Protein Preparation Wizard where atom and bond types were corrected and protonation states of ionizable species adjusted to pH 7.4 + 1.0 by Epik.90. These models were minimized using the OPLS_2005 forcefield to a converged heavy atom RMSD ≤ 0.3Å. Ligands were prepared using LigPrep with protonation states set at pH 7.4 + 1.0 by Epik and structures minimized using the OPLS_2005 forcefield. Docking of ligands into proteins were performed using Schrödinger (Maestro Version 12.8). In Schrödinger, the Receptor Grid generated by picking the default ligand molecule. XP mode was performed in molecular docking, and the final result was chosen according to the Glide scoring function.

### 2.11 Statistical analysis

Differences between mean values of normally distributed data were evaluated by Student’s t-test using SPSS 25.0 software (SPSS Inc., Chicago, IL, United States). *p* < 0.05 was considered as significant difference. All the data were expressed as mean ± SD.

## 3 Results

### 3.1 SF-AM herb pair alleviates liver pathological damage in hepatitis-cirrhosis-HCC rats

**FIGURE 2:**
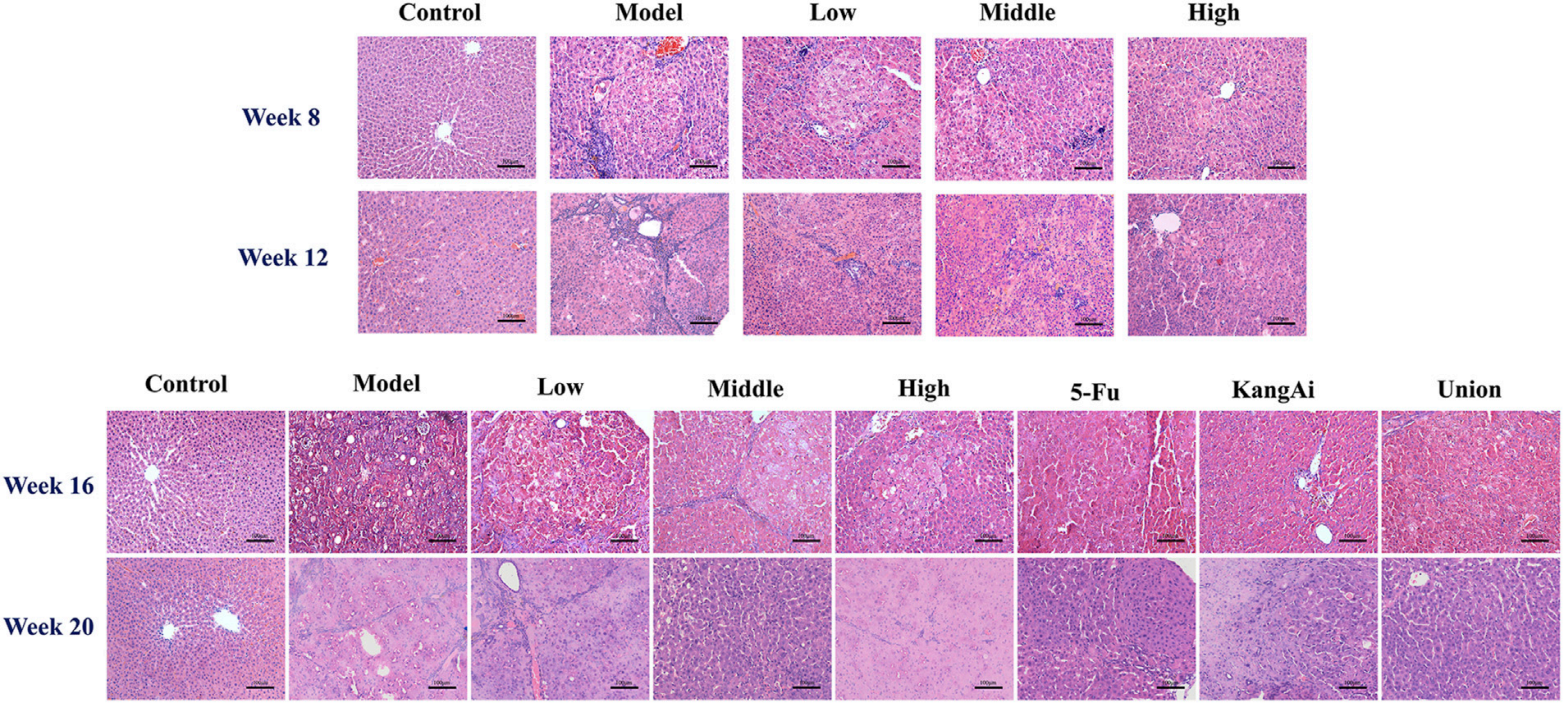
HE staining results of the liver in different groups of rats at hepatitis-cirrhosis-HCC stages.

At week 8, the changes of liver in the model group could not be seen by naked eye (Supplementary Figure S3), but some inflammatory cells were observed under light microscope (Figure 2). At week 12, the surface of the liver was gradually rough, with nearly circular lesions. HE staining assay revealed that liver cells were swollen with obvious fibrosis and apparent false lobules. At week 16 and week 20, the rats in model group exhibited numerous gray nodules of liver cancer, accompanied by bleeding and necrosis. Under a light microscope, the hepatic plate structure of the model group appeared disorganized, with many hepatocytes showing pyknotic chromatin, prevalent necrotic cells, and certain areas displaying vacuoles. Additionally, the size of cancerous cells varied. These results indicated that our model went through three stages: hepatitis (week 8), cirrhosis (week 12), and HCC (week 16 and week 20). After treatment with SF-AM herb pair, these symptoms were all improved in different degrees.

### 3.2 SF-AM herb pair reverses the metabolic abnormality in hepatitis-cirrhosis-HCC rats

**FIGURE 3:**
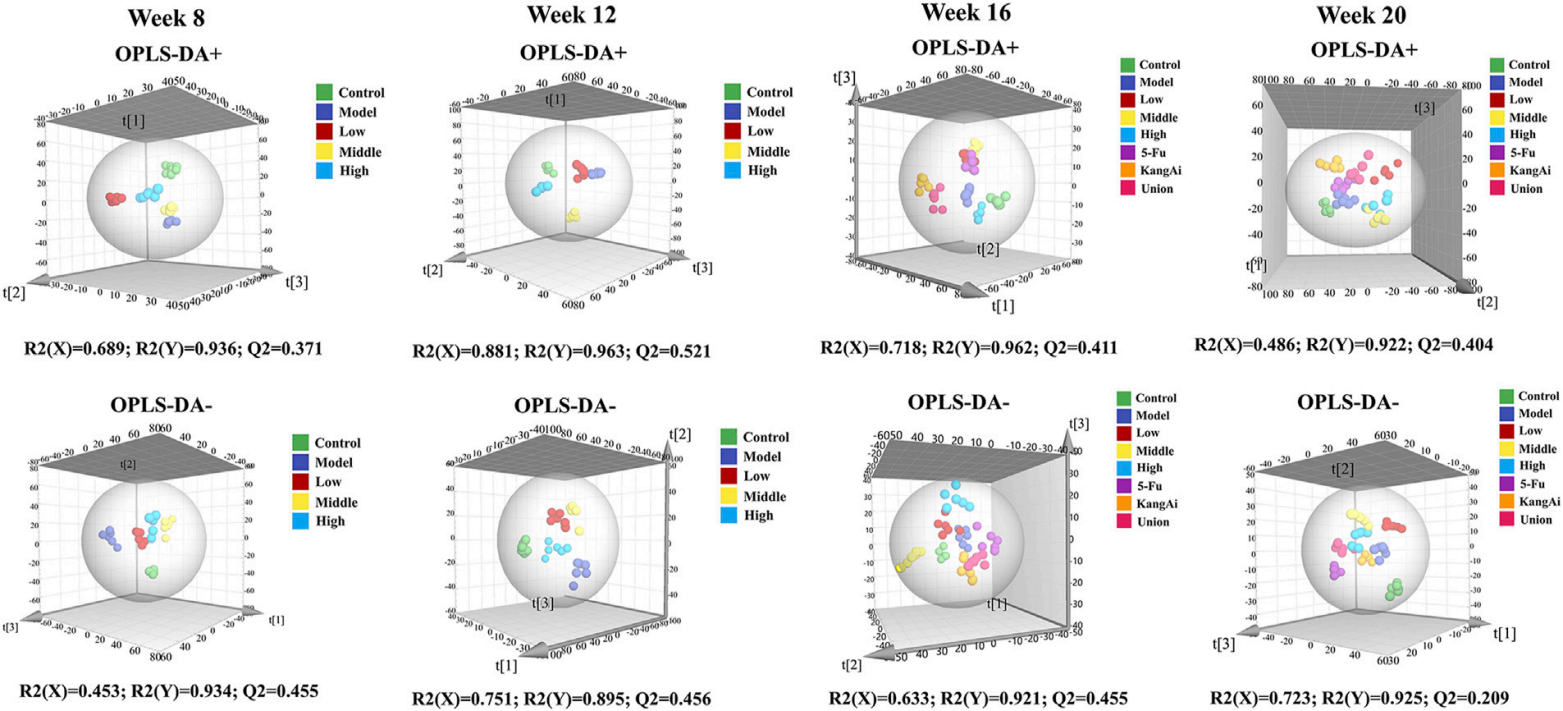
OPLS-DA score plot of different groups of rats at hepatitis-cirrhosis-HCC stages.

**FIGURE 4:**
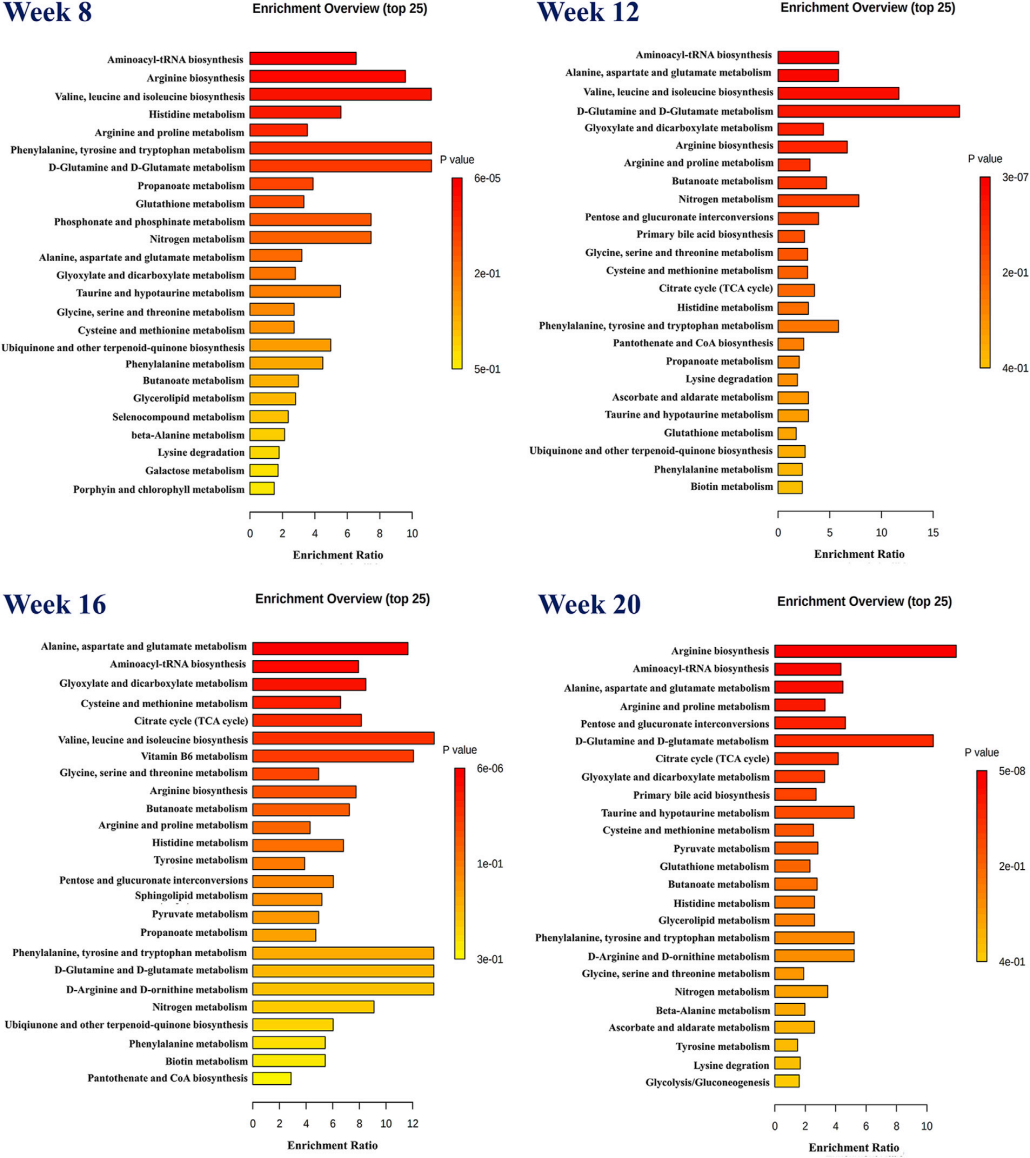
Pathway enrichment analysis based on biomarkers in hepatitis-cirrhosis-HCC stages.

To investigate the effect of SF-AM herb pair on hepatitis-cirrhosis-HCC rats at the metabolite level, a validated HPLC-Q-TOF-MS/MS method was used to investigate the metabolic profiles before and after drug treatment, the details of methodological validation were provided in Supplementary Tables S5, S6. Analysis and visualization of TOF-MS information from different samples were conducted via OPLS-DA in both positive and negative modes without the risk of overfitting (Supplementary Figure S4), and obvious separations were observed among all groups (Figure 3). Endogenous metabolites with *p* < 0.05 and VIP > 1 were chosen as potential biomarkers. As listed in Supplementary Table S7, we identified 37, 66, 70 and 76 potential biomarkers in rat plasma at week 8, 12, 16 and 20, respectively. Pathway enrichment analysis (Figure 4) showed that many amino acid-related metabolic pathways, such as aminoacyl-tRNA biosynthesis, arginine biosynthesis, valine, leucine and isoleucine biosynthesis, and D-glutamine and D-glutamate metabolism were disturbed in hepatitis, cirrhosis, and HCC rats. At week 8, arginine biosynthesis, valine, leucine and isoleucine biosynthesis, histidine metabolism and many other metabolic pathways were abnormal. Starting from week 12, apart from some amino acid metabolic pathways, primary bile acids and the TCA cycle were also disturbed. During the HCC stage at week 16 and week 20, many energy-related metabolism pathways, such as many amino acid metabolic pathways and the TCA cycle, were continuously activated to support the material and energy requirement for the massive proliferation of liver cancer cells. In addition, pyruvate metabolism was disturbed at week 16 and week 20, leading to abnormal energy metabolism.

After treatment with SF-AM herb pair, 9, 15, 30, and 12 differential endogenous metabolites were recalled at week 8, 12, 16, and 20, respectively (Supplementary Tables S8, S9). The results revealed that these metabolites mainly regulate amino acids and organic acids metabolites, including 2-ketobutyric acid, L-threonine, L-glutamic acid, benzoic, L-tyrosine, L-methionine, pyruvic acid, and so on. Furthermore, the results suggested that SF-AM herb pair may had a more pronounced effect at week 8, week 12 and week 16, and a weaker effect at week 20, based on the callback degree of the OPLS-DA score plot and the number of recalled metabolites.

### 3.3 The absorbed component of SF-AM herb pair in the plasma of hepatitis-cirrhosis-HCC rats

**FIGURE 5:**
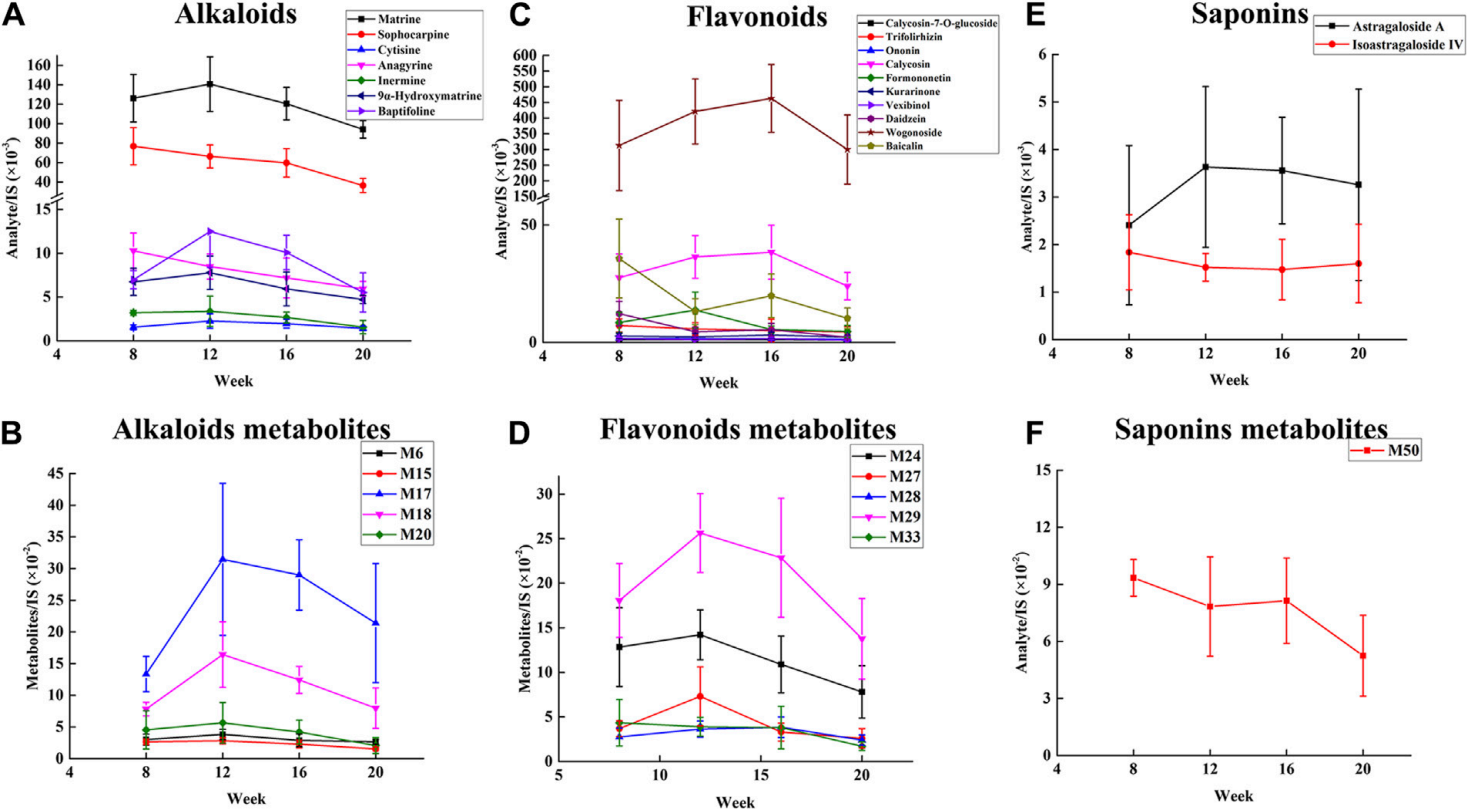
Relative concentration changes of prototypical components and metabolites of SF-AM herb pair in the plasma of hepatitis-cirrhosis-HCC rats. **(A,B)** Relative concentration changes of alkaloids and their representative metabolites; **(C,D)** Relative concentration changes of flavonoids and their representative metabolites; **(E,F)** Relative concentration changes of saponins and their representative metabolites.

**FIGURE 6:**
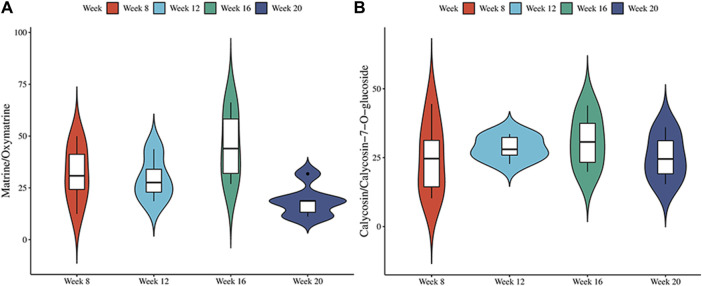
The metabolic conversion ratio of SF-AM herb pair *in vivo*. **(A)** The ratio of matrine/oxymatrine; **(B)** The ratio of calycosin/calycosin-7-O-glucoside.

To identify the therapeutic material basis of SF-AM herb pair in the development of HCC, we analyzed and compared the prototypical components and metabolites of SF-AM herb pair in the plasma of hepatitis-cirrhosis-HCC rats by HPLC-Q-TOF-MS/MS. The precision, repeatability, and sample stability of the developed method were all within acceptable limits (Supplementary Table S10). By analyzing mass errors, retention times and secondary fragmentation information for reference controls, 23 prototypical components and 53 metabolites were found at each stage (Supplementary Tables S11, S12). Although the types of components entering the blood were same at different stages, their concentrations in the blood varied (Supplementary Tables S13, S14). The concentrations of most of the prototypical components were highest at week 8 and week 12, but decreased at week 16 and week 20. The metabolites reached their highest levels at week 12 and 16 and decreased by week 20, aligning with the trend observed in the prototypical components (Figure 5). This indicated the absorption or metabolism of the drug in rats were poorer in HCC. To further study the metabolism of the drug *in vivo*, we analyzed the ratio of matrine/oxymatrine and calycosin/calycosin 7-O-glucoside. Matrine and calycosin can be metabolized from oxymatrine and calycosin 7-O-glucoside by CYP450 enzymes (mainly CYP3A4) *in vivo*, respectively. As shown in Figure 6, the proportions of matrine/oxymatrine and calycosin/calycosin 7-O-glucoside were not significantly different in the early stage of HCC, but decreased in late stage (week 20), suggesting that the metabolism of drugs might be decreased. CYP450 enzymes are a class of metabolic enzymes present in liver microsomes, of which CYP3A4 catalyzes the metabolism of many drugs (Zhang et al., 2022). It can be used as a terminal oxygenase to participate in the metabolism of most antitumor drugs and exogenous compounds. Many literatures have reported that the expression of CYP3A4 was decreased in patients with HCC (Ashida et al., 2017), which also verified our assumption.

### 3.4 SF-AM herb pair regulates the CD4^+^ and CD8^+^ T lymphocytes in the liver of hepatitis-cirrhosis-HCC rats

**FIGURE 7:**
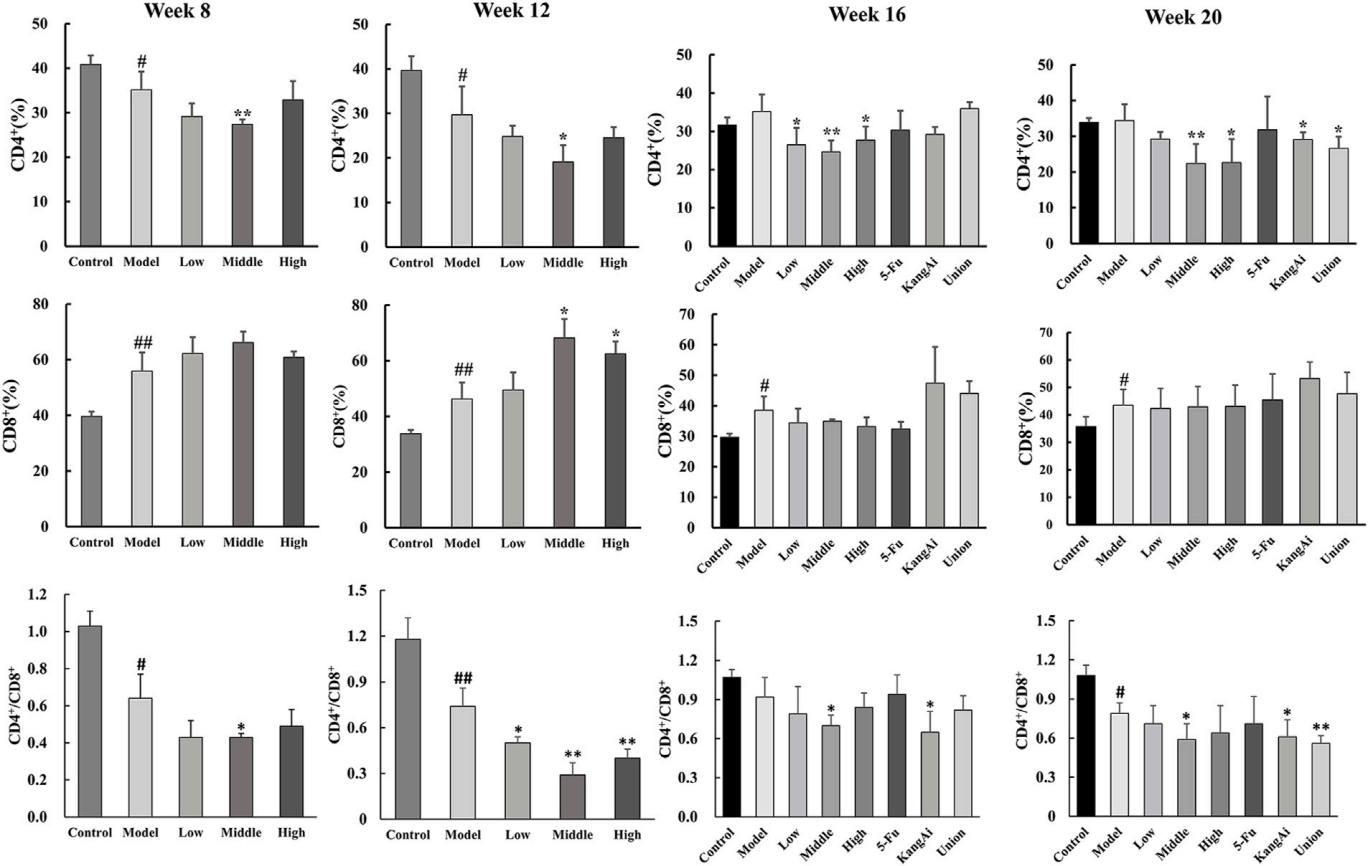
Expression of CD4^+^, CD8^+^, and CD4^+^/CD8^+^ T lymphocytes in rat liver at hepatitis-cirrhosis-HCC stages. ^#^ *p* < 0.05, ^##^ *p* < 0.01, compared with control group; *p < 0.05, **p < 0.01, compared with model group.

To explore how the SF-AM herb pair affected the regulation of immunity, CD4^+^ and CD8^+^ T lymphocytes in liver tissues were detected by flow cytometry at the 8th, 12th, 16th, and 20th week (Supplementary Figure S5). Starting from week 8, the percentage of CD4^+^ T lymphocyte decreased in the model group while CD8^+^ T lymphocytes increased, and the ratio of CD4^+^ to CD8^+^ decreased (Figure 7). After treatment, the percentage of CD8^+^ T lymphocyte continued to rise, and the CD4^+^/CD8^+^ ratio was decreased. CD8^+^ T lymphocytes are mainly cytotoxic T lymphocytes, which can release perforin and particle enzymes, destroy and decompose tumor cells, and trigger apoptosis of target cells. However, at week 16 and week 20, the SF-AM herb pair only had the effect of decreasing CD4^+^/CD8^+^, indicating that the drugs are less effective on the immune system at the 16th and 20th week than at the 8th week and 12th week. In addition, the immunoregulatory ability of SF-AM herb pair was similar to that of KangAi injection, and the combination of SF-AM herb pair with 5-Fu was better than 5-Fu alone, indicating that the SF-AM herb pair can be used as adjuvant therapy in clinical practice.

### 3.5 SF-AM herb pair alleviates hepatitis-cirrhosis-HCC via regulating PI3K/Akt pathway related protein and mRNA expression

**FIGURE 8:**
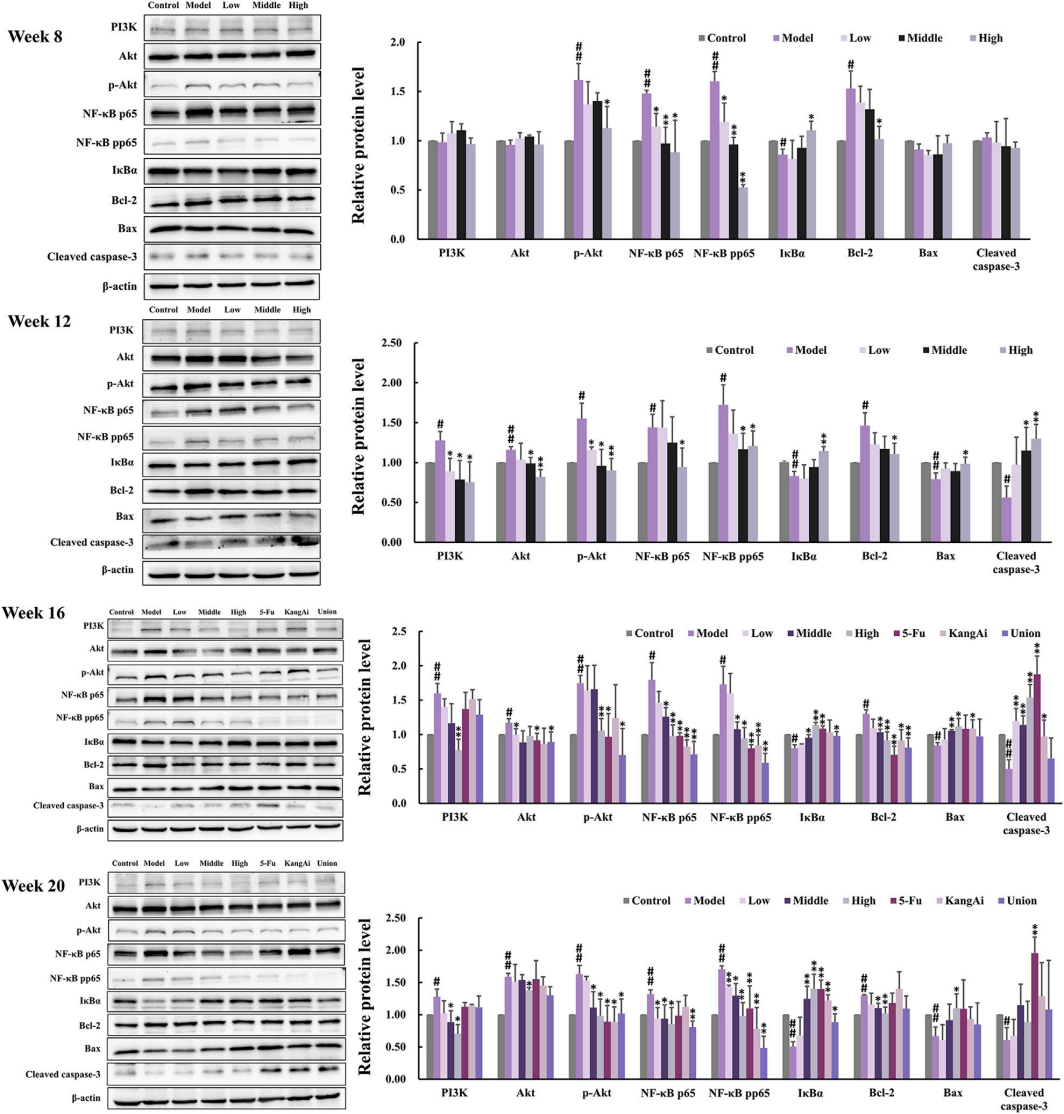
Typical protein bands and relative expression of PI3K, Akt, p-Akt, NF-κB p65, NF-κB pp65, IκBα, Bcl-2, Bax and cleaved caspase-3 in Western blot analysis (*n* = 3 per group). ^#^ *p* < 0.05, ^##^ *p* < 0.01, compared with control group; *p < 0.05, **p < 0.01, compared with model group.

**FIGURE 9:**
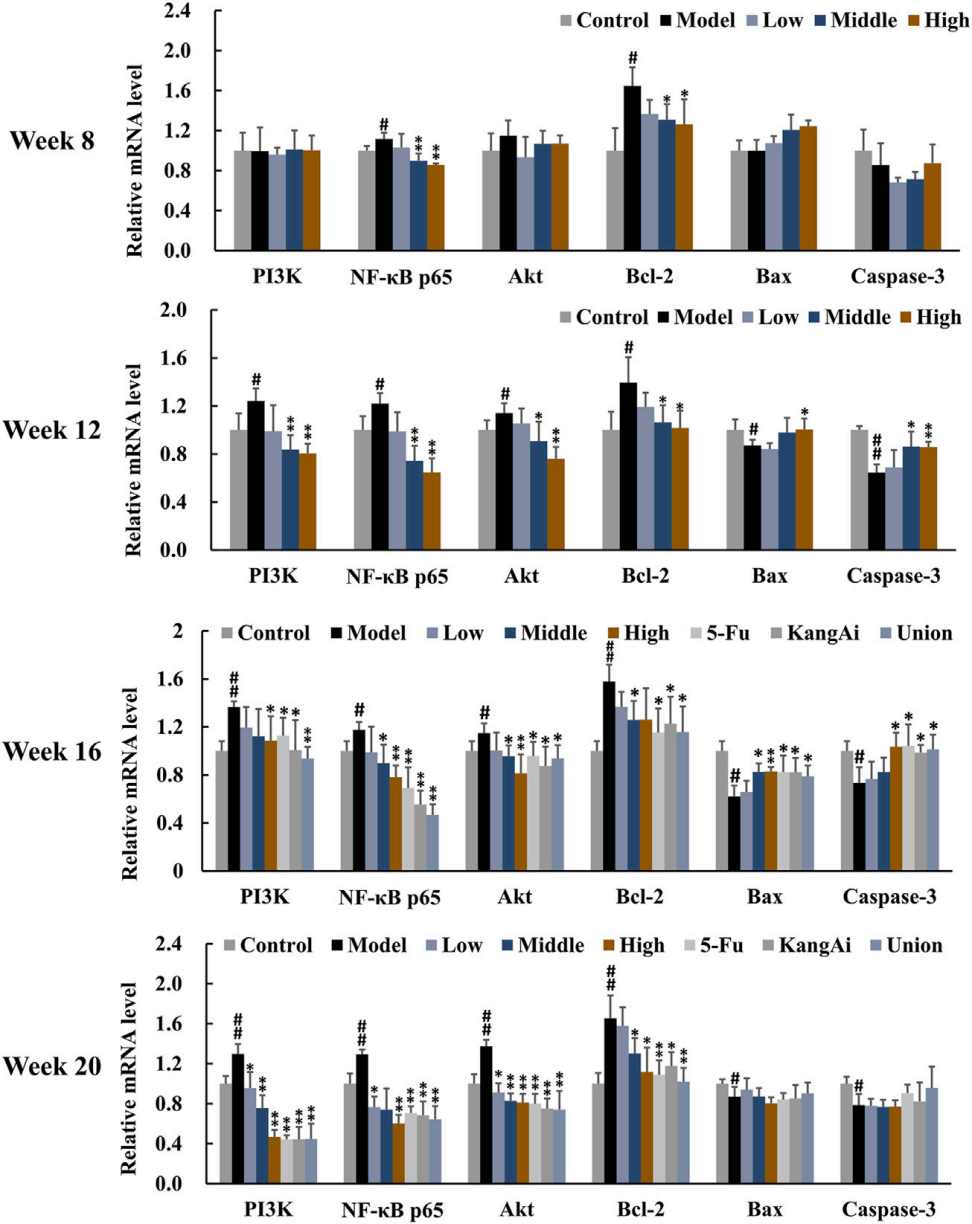
The mRNA expression of PI3K, NF-κB p65, Akt, Bcl-2, Bax, and cleaved caspase-3 in rat liver in the development of HCC (normalized to GAPDH). ^#^ *p* < 0.05, ^##^ *p* < 0.01, compared with control group; *p < 0.05, **p < 0.01, compared with model group.

To investigate the effect of SF-AM herb pair on inflammation and apoptosis, we determined the levels of inflammation and apoptosis-related proteins and mRNAs in the PI3K/Akt signaling pathway by Western blotting (Figure 8) and qRT-PCR (Figure 9), respectively. At inflammation stage of week 8, the protein expression levels of p-Akt, NF-κB p65, NF-κB pp65, Bcl-2, and the mRNA levels of NF-κB p65 and Bcl-2 in the model group were obviously increased, whereas the protein level of IκBα was decreased, indicating that the inflammatory and apoptosis pathways downstream of PI3K/Akt were activated at this time. The levels of proteins and mRNAs were recalled after treatment with SF-AM herb pair. At week 12 and week 16, the expression levels of PI3K, Akt, p-Akt, NF-κB p65, NF-κB pp65, and Bcl-2 in the model group were all increased, while IκB, Bax and cleaved caspase-3 were decreased. These results demonstrated that the PI3K/Akt signaling pathway was further disturbed with the development of HCC. After treatment with SF-AM herb pair, all the proteins and mRNA levels had a significant pullback trend, indicating that SF-AM had anti-inflammatory effects and promoted apoptosis. At week 20, the SF-AM herb pair also reversed the expression of all proteins and their mRNAs except for Bax and cleaved caspase-3. Thus, PI3K, Akt, NF-κB p65, and Bcl-2 might be the main targets of SF-AM herb pair throughout hepatitis-cirrhosis-HCC progression.

### 3.6 SF-AM herb pair inhibits the proliferation of HCC cells and regulates PI3K/Akt pathway related protein expression *in vitro*

**FIGURE 10:**
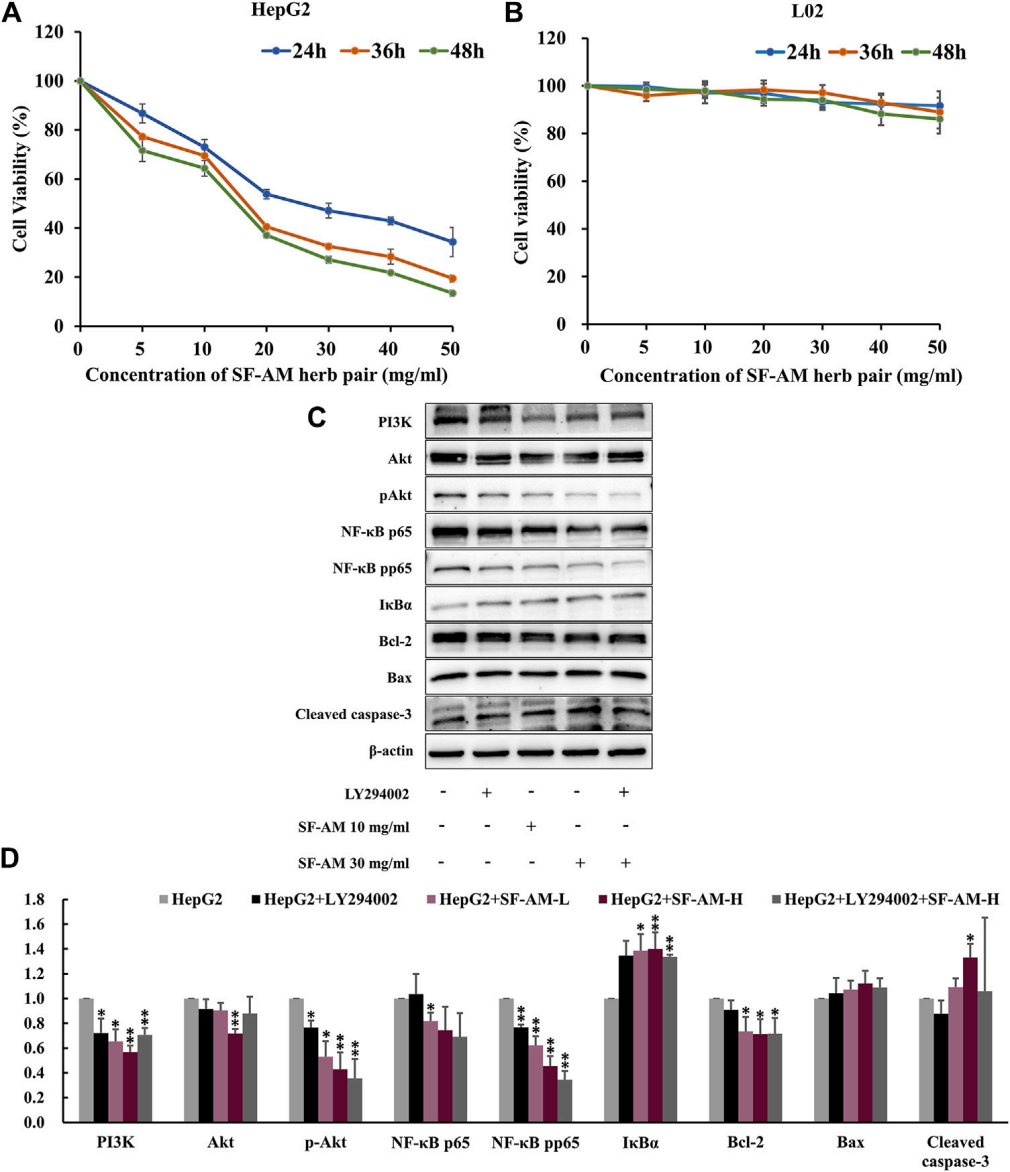
*In vitro* cell experiment validation. **(A,B)** Cell viability test of HepG2 and L02 cell line treated with different concentrations of SF-AM herb pair for 24, 36, and 48 h. **(C,D)** Typical Western blot strips and Densitometric analysis of expression of PI3K, Akt, p-Akt, NF-κB p65, NF-κB pp65, IκBα, Bcl-2, Bax, and cleaved caspase-3. Mean ± SD was calculated for all experiment values. ^*^ *p* < 0.05, ^**^ *p* < 0.01, compared with HepG2 group.

As shown in Figures 10A, B, SF-AM herb pair inhibited the proliferation of HepG2 cell in both time- and dose-dependent manner, but had no obvious effect on L02 cell. After treatment with PI3K inhibitor, LY294002, the expression level of PI3K was significantly lowered. Similarly, the groups treated with SF-AM herb pair showed an obvious decrease in the protein levels of PI3K, Akt, p-Akt, NF-κB p65, NF-κB pp65, IκBα, Bcl-2/Bax, as well as an increased level of cleaved caspase-3 (Figures 10C, D), which was consistent with the results of the animal experiments.

### 3.7 Exploration of the potential therapeutic substances of SF-AM herb pair during the development of HCC

**FIGURE 11:**
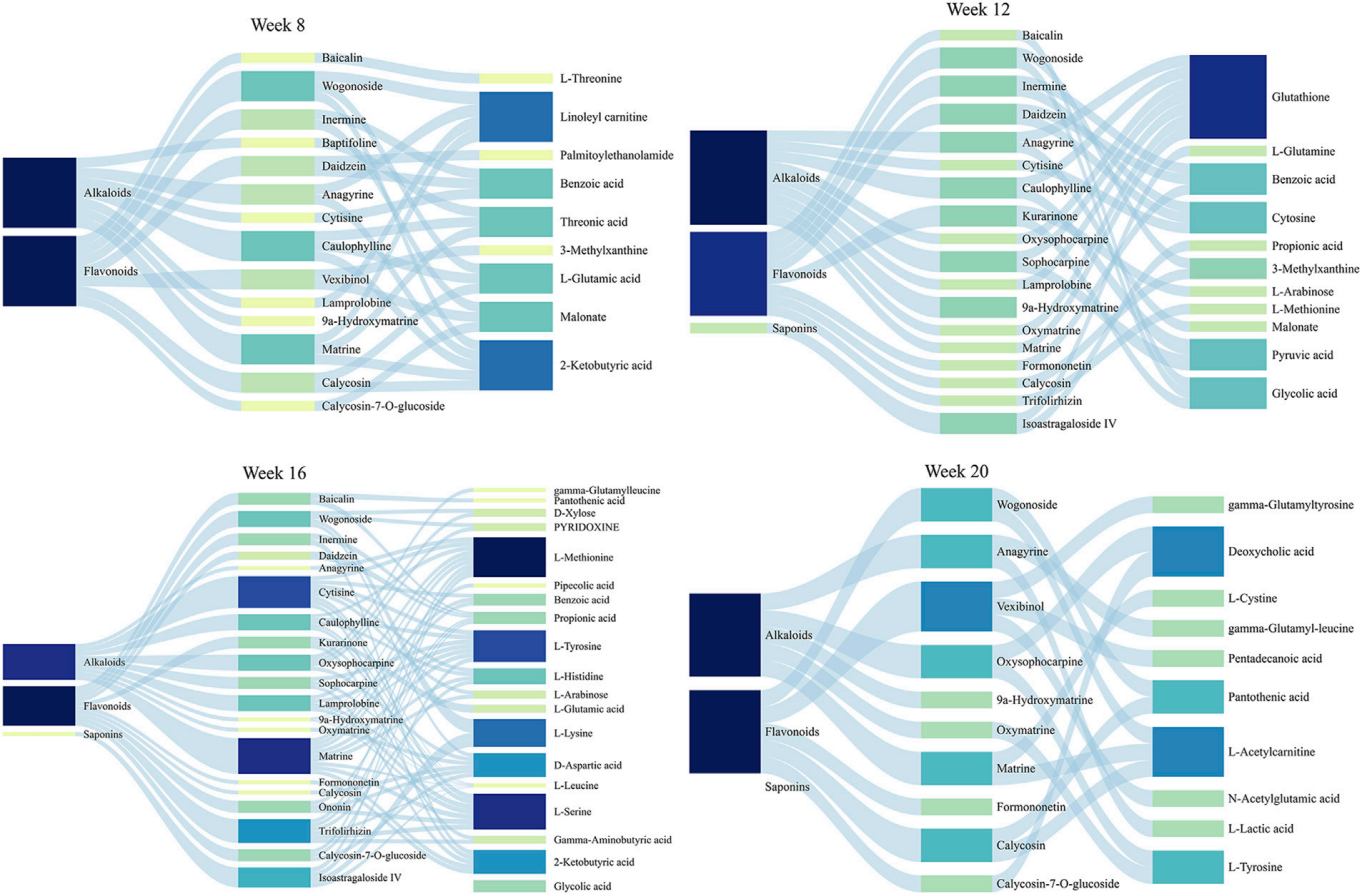
Sanky diagram for the correlation between chemical components *in vivo* and all determined pharmacodynamic indicators.

To identify the potential therapeutic substances of SF-AM herb pair in the treatment of HCC, Pearson correlation analysis was used to examine the relevance between pharmacodynamic indicators and normalized semi-quantify results (A_analytes_/A_IS_) of 23 absorbed prototypical components in rat plasma. Metabolites regulated by drug were considered as pharmacodynamic indicators. The components with │γ│ ≥ 0.6 were selected as potential bioactive components, as shown in Figure 11. The results indicated that SF-AM herb pair exerts anti-HCC effect through multi-targets with multi-components. The main absorbed components of SF-AM herb pair include 2 saponins, 11 flavonoids, and 10 alkaloids. It is evident that the potential therapeutic substances of SF-AM herb pair in different stages are different. For example, at week 8, wogonoside, caulophylline, and matrine were the most relevant components associated with the pharmacodynamic indicators. At week 12, many saponins, flavonoids and alkaloids, such as wogonoside, inermine, daidzein, sophocarpine, 9α-hydroxymatrine, and isoastragaloside IV, were the components most significantly associated with efficacy. As the disease further progressed, matrine, cytisine, trifolirhizin, isoastragaloside IV, and other components gradually played a greater role. In total, according to our results, we predict that the therapeutic substances of SF-AM herb pair may vary during the progression of HCC, and their efficacy may be achieved by the joint action of multiple components.

### 3.8 Molecular docking between potential therapeutical substances and core targets

**FIGURE 12:**
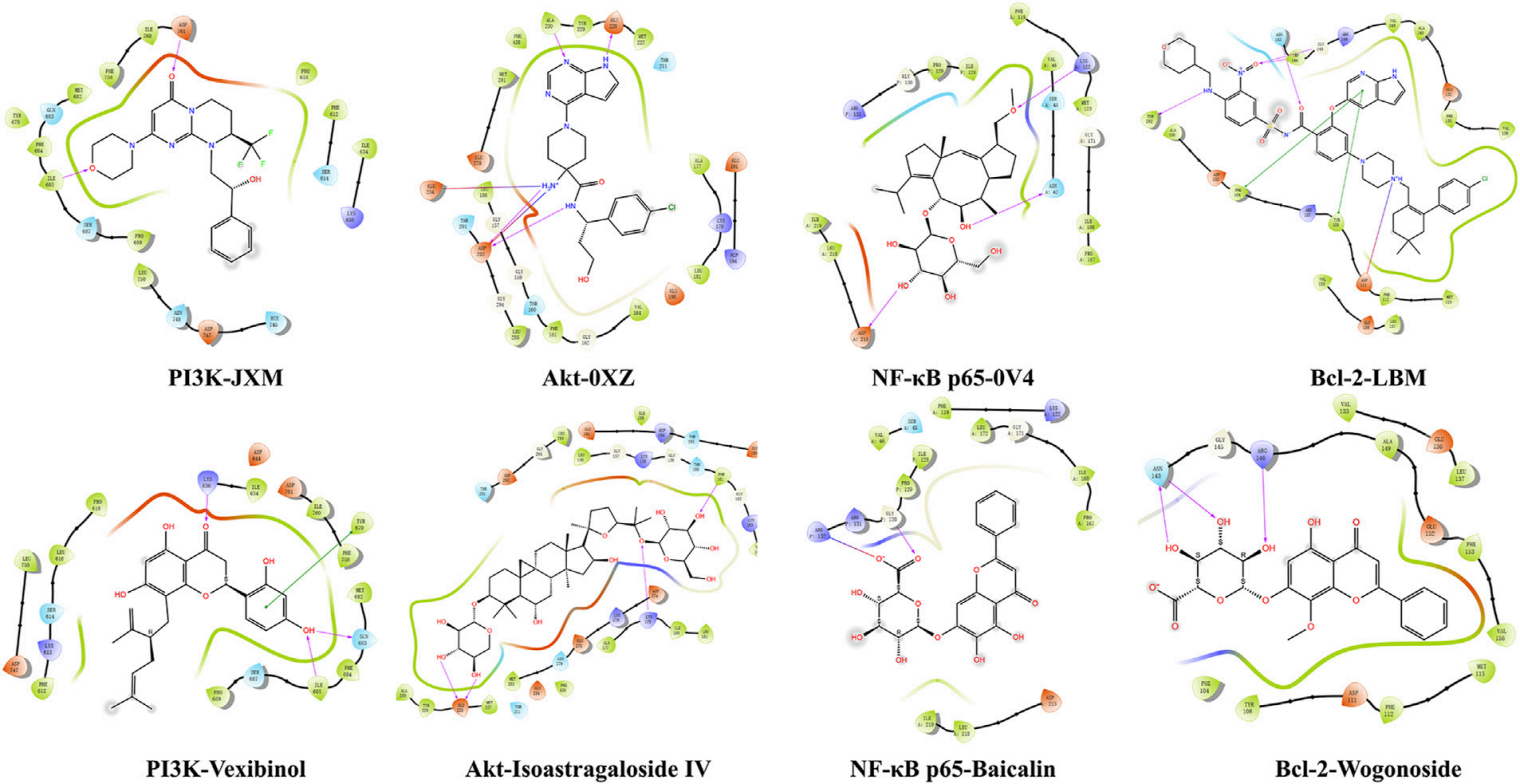
Typical molecular docking interaction diagrams of active components with macromolecular ligands.

To further validate the pharmacodynamic substance of SF-AM herb pair, molecular docking was conducted between PI3K, Akt, NF-κB p65, Bcl-2 and active components, respectively. The details of binding site, docking score, and glide energy were listed in Supplementary Tables S15–S18. The results showed that ILE685, ASP761 were the main active sites of PI3K, ASP292, ALA230, and GLU228 were the main active sites of Akt, ASP215, ASN42, and LYS122 were the main active sites of NF-κB p65, and TYR202, ASN143, TRP144, GLY145 were the main active sites of Bcl-2. Moreover, the docking scores of vexibinol, isoastragaloside IV, baicalin, wogonoside, kurarinone, daidzein, calycosin, formononetin, ononin, calycosin-7-O-glucoside, inermine, oxysophocarpine, trifolirhizin, astragaloside A, oxymatrine, sophocarpine, cytisine, matrine, and caulophylline with at least one protein were less than −5 kcal/mol, and the binding sites were similar to positive drugs. Thus, the above components were identified as key active components. Several typical molecular docking diagrams were listed in Figure 12.

## 4 Discussion

In TCM theory, “strengthening the body” means enhancing the body’s resistance, which is equivalent to boosting the body’s immunity. “Eliminating pathogens” refers to the elimination of various harmful factors that destroy the relative balance between the human body and the environment. Chronic disequilibrium between Yin and Yang caused by interior and exterior factors leads to stasis of “*qi*” (vital energy), blood, dampness and phlegm, and then produced pathogenic heat and toxins, which was similar to inflammation. Tumor cells produced in the progress of diseases are also classified as pathogens. As a classic herb pair for “strengthening the body and eliminating pathogens,” SF-AM herb pair is a promising treatment for cancer.

Considering that HCC often develops from hepatitis or cirrhosis, it is essential to investigate the effect of SF-AM herb pair on the progression of hepatitis, cirrhosis, and HCC. In this study, DEN was used to induce an HCC model that has genetic and molecular features similar to those of human HCC (Kurma et al., 2021). The model experienced three processes: hepatitis (week 8), cirrhosis (week 12), HCC (week 16 and week 20). Based on the results of HE staining and non-targeted metabolomics, SF-AM herb pair had better therapeutic effects on week 8 and week 12 than week 16 and week 20, indicating that SF-AM herb pair may obtain more benefit in the early prevention of HCC.

Cancer cells undergo a profound reprogramming of their metabolism to fulfill their energy and biosynthesis requirements (Schiliro and Firestein, 2021). Our results showed that many amino acid-related metabolic pathways and TCA cycle were disturbed in the development of HCC, which are consistent with current researches (Fitian et al., 2014; Liu et al., 2014; Ferrarini et al., 2019). Amino acids not only serve as crude materials for synthesizing energy and proteins, but also act as signal molecules. 2-Ketobutyric acid, also known as 2-oxobutanoate, is an intermediate in the metabolism of amino acids, especially in the synthesis of valine, leucine, and isoleucine, as well as in the metabolism of glycine, serine, and threonine. The level of 2-ketobutyric acid is elevated in hepatitis, cirrhosis, and HCC, while decreased after administration. Valine, leucine and isoleucine are all branch chain amino acids (BCAAs), and the loss of BCAA catabolism leads to the accumulation of BCAAs in tumors, thereby promoting tumor development and growth (Ericksen et al., 2019). In addition to serving as building blocks for the synthesis of nitrogenous chemicals, BCAAs also function as signaling molecules that control immunity, lipid and protein synthesis, and glucose metabolism through a specific signaling network, particularly the PI3K/AKT/mTOR signal pathway (Nie et al., 2018). L-glutamic acid can be synthesized by glutamine synthase, and glutamine can be metabolized to produce α-ketoglutaric acid and oxaloacetic acid, thereby supporting the TCA cycle (Hensley et al., 2013). Additionally, L-glutamic acid could provide sufficient raw materials for the synthesis of glutamine to meet the material and energy requirements of rapid proliferation of cancer cells. Our study found that L-glutamic acid was significantly increased in rats during the development of HCC and decreased after treatment with SF-AM herb pair. Research has shown glutamine is crucial for the growth of lymphocytes and the synthesis of cytokines (Cruzat et al., 2018), indicating that SF-AM herb pair may further affect the body’s immunity and inflammation.

Immunotherapy could enhance the ability of the immune system to control the progression of HCC. Hence, immunotherapy has sparked new hope for the treatment of HCC (Zhou et al., 2022). Although considerable literatures have reported that both SF and AM have immunomodulatory effects individually (Chen et al., 2020; Alhazmi et al., 2021; Li et al., 2021), there is no report on the immunomodulatory effect of SF-AM herb pair. In our study, we found that SF-AM herb pair could enhance immunity by increasing the ratio of cytotoxic CD8^+^ T lymphocyte, which can release perforin and particle enzymes to destroy and disintegrate tumor cells and cause target cells to undergo apoptosis. In addition, the immune-regulation effect of SF-AM herb pair is similar to KangAi injection, an anti-tumor Chinese patent medicine containing AM and kushenin, which is commonly used in clinic since it has a major efficacy on Nourishes Qi and Righteousness. Modern pharmacological researches have suggested that KangAi injection may have anticancer benefits by enhancing the immune system, triggering cell apoptosis, and preventing cell proliferation, invasion, and metastasis (Wan et al., 2018; Li et al., 2019; Song et al., 2020). This also proved that SF-AM herb pair could enhance immunity. Moreover, the immunomodulatory effect of SF-AM herb pair was more obvious in the hepatitis and cirrhosis stages, and the combination with 5-Fu was better than 5-Fu alone, indicating that the SF-AM herb pair can be used for early prevention and as adjuvant therapy for HCC in clinical practice.

PI3K/Akt signaling pathway is highly altered and activated in a variety of malignancies since it regulates cell proliferation, survival, migration, inflammation, and angiogenesis, and is associated with poor prognosis and survival in HCC patients (Guerrero-Zotano et al., 2016). NF-κB signaling pathway is the downstream of PI3K/Akt, which actively participates in inflammation through factors like NF-κB p65, whose level might indicate the severity of inflammation. Chronic inflammation may cause fibrosis, cirrhosis and HCC due to its association with ongoing hepatic injury and regeneration. Our study found that the PI3K/Akt/NF-κB signal pathway was activated in the development of HCC. After PI3K activation, Akt was phosphorylated, thereby inducing the expression of members of the NF-κB family like p65. SF-AM herb pair can significantly inhibit the expression of Akt and p-Akt, and then suppress the phosphorylation of NF-κB p65 as well as the degradation of IκBα. Bcl-2, Bax, and cleaved caspase-3 are apoptosis-related proteins, Bax and cleaved caspase-3 promote apoptosis, while Bcl-2 inhibit apoptosis. Apoptosis is inhibited in the tumor and thus promotes tumor metastasis (Zhu et al., 2019). SF-AM herb pair can promote apoptosis by upregulating the ratio of Bax/Bcl-2 and cleaved caspase-3 at both the protein and mRNA levels. Additionally, the SF-AM herb pair had the ability of regulating PI3K, Akt, NF-κB p65, and Bcl-2 in the whole process of hepatitis-cirrhosis-HCC at both the protein and mRNA levels, suggesting that they might be the main targets of SF-AM herb pair.

Systematic metabolomics is consistent with the holistic view of TCM, which has been widely used in TCM research. To investigate the potential therapeutic substances, Pearson corelation analysis was conducted between the absorbed prototypical components and biomarkers recalled by SF-AM herb pair. Although the therapeutic substances in different stages are not exactly the same, flavonoids, alkaloids, and saponins are all the main pharmacodynamic material basis of SF-AM herb pair. At week 8, wogonoside, matrine, and caulophylline are the most relevant components correlated with the pharmacodynamic indicators. Wogonoside can inhibit inflammatory cytokine production in LPS-stimulated macrophage by suppressing the activation of the JNK/c-Jun signaling pathway (Yu et al., 2020). Matrine is the index components for content determination in the Chinese Pharmacopoeia (2020 edition) of SF, and it is reported to have many pharmacological activities, including anti-inflammatory, anti-fibrosis, and anti-cancer (Yu et al., 2014; Wang et al., 2021). Moreover, matrine has been demonstrated efficacy in preventing HCC migration and proliferation *in vitro*, and it can prevent the early lesions of HCC in rat liver (Shi et al., 2019). At week 12, many saponins, flavonoids and alkaloids, such as wogonoside, inermine, daidzein, sophocarpine, 9α-hydroxymatrine, and isoastragaloside IV, are the components most significantly associated with efficacy. The total flavonoids of AM are reported to have immunomodulatory and anti-inflammatory effects by controlling NF-κB and MAPK signaling pathways (Li J. et al., 2018). Alkaloids, including matrine, sophocarpine, 9α-hydroxymatrine, are the main activity components of SF, and most of them are reported to have anti-inflammatory and anti-HCC activity (Dai et al., 2021). In addition, saponins are also the main active components of AM with anti-inflammatory and anticancer effect (Ionkova et al., 2010; Li Y. et al., 2018). As the disease progresses, matrine, cytisine, trifolirhizin, isoastragaloside IV, and other components, gradually played a greater role. Molecular docking technique is a simulation method that can predict binding patterns and affinity, and visualize the interaction between receptor and drug molecule. By referring the results of molecular docking, we found that most of the potential therapeutical substances bind well with PI3K, Akt, NF-κB p65, and Bcl-2. In total, the therapeutic substances of SF-AM herb pair were different in the progression of HCC, and their efficacy could be achieved by the joint action of multiple components.

## 5 Conclusion

In this study, we demonstrated that SF-AM herb pair could alleviate the liver pathological damage, reverse abnormal metabolism in hepatitis-cirrhosis-HCC rats, and enhance immunity, anti-inflammatory, and promoting apoptosis via regulating the percentage of CD4^+^ and CD8^+^ T lymphocytes, as well as PI3K/Akt pathway-related proteins and mRNAs, especially by targeting PI3K, Akt, NF-κB p65, Bcl-2. However, the therapeutic effect of SF-AM herb pair was more effective at hepatitis and cirrhosis stages compared to HCC, suggesting that SF-AM herb pair might be a potential therapeutic candidate in blocking the progress of hepatitis-cirrhosis-HCC axis. Moreover, Pearson correlation analysis and molecular docking demonstrated that SF-AM herb pair could exert effect through multi-targets and multi-components. Overall, this study revealed the efficacy, mechanisms and therapeutic substances of SF-AM herb pair on hepatitis-cirrhosis-HCC and provided an experimental basis for the clinical application of the SF-AM herb pair.

## Data Availability

The original contributions presented in the study are included in the article/Supplementary Material, further inquiries can be directed to the corresponding author.

## Abbreviations

5-Fu, 5-Fluorouracil; AM, *Astragalus mongholicus*; BCAAs, branch chain amino acids; DEN, diethylnitrosamine; HCC, hepatocellular carcinoma; HE, hematoxylin-eosin; qRT-PCR, quantitative real-time polymerase chain reaction; SF, *Sophora flavescens*; TCM, traditional Chinese medicine.

## References

[B1] Alhazmi H. Najmi A. Javed S. Sultana S. Bratty M. Al Makeen H. (2021). Medicinal plants and isolated molecules demonstrating immunomodulation activity as potential alternative therapies for viral diseases including COVID-19. Front. Immunol. 12, 637553. 10.3389/fimmu.2021.637553 34054806 PMC8155592

[B2] Ashida R. Okamura Y. Ohshima K. Kakuda Y. Uesaka K. Sugiura T. (2017). CYP3A4 gene is a novel biomarker for predicting a poor prognosis in hepatocellular carcinoma. Cancer Genomics Proteomics 14, 445–453. 10.21873/cgp.20054 29109094 PMC6070324

[B3] Chen Z. Liu L. Gao C. Chen W. Vong C. Yao P. (2020). Astragali Radix (Huangqi): a promising edible immunomodulatory herbal medicine. J. Ethnopharmacol. 258, 112895. 10.1016/j.jep.2020.112895 32330511

[B4] Cruzat V. Rogero M. M. Keane K. N. Curi R. Newsholme P. (2018). Glutamine: metabolism and immune function, supplementation and clinical translation. Nutrients 10, 1564. 10.3390/nu10111564 30360490 PMC6266414

[B5] Cubero F. J. (2016). Shutting off inflammation: a novel switch on hepatic stellate cells. Hepatology 63, 1086–1089. 10.1002/hep.28442 27008200

[B6] Dai M. Chen N. Li J. Tan L. Li X. Wen J. (2021). *In vitro* and *in vivo* anti-metastatic effect of the alkaliod matrine from Sophora flavecens on hepatocellular carcinoma and its mechanisms. Phytomedicine 87, 153580. 10.1016/j.phymed.2021.153580 34029939

[B7] Ericksen R. Lim S. Mcdonnell E. Shuen W. Vadiveloo M. White P. (2019). Loss of BCAA catabolism during carcinogenesis enhances mTORC1 activity and promotes tumor development and progression. Cell Metab. 29, 1151–1165. 10.1016/j.cmet.2018.12.020 30661928 PMC6506390

[B8] Feng J. Li J. Wu L. Yu Q. Ji J. Wu J. (2020). Emerging roles and the regulation of aerobic glycolysis in hepatocellular carcinoma. J. Exp. Clin. Cancer Res. 39, 126. 10.1186/s13046-020-01629-4 32631382 PMC7336654

[B9] Ferrarini A. Poto C. D. He S. Tu C. Varghese R. Balla A. (2019). Metabolomic analysis of liver tissues for characterization of hepatocellular carcinoma. J. Proteome Res. 18, 3067–3076. 10.1021/acs.jproteome.9b00185 31188000 PMC6677583

[B10] Fitian A. I. Nelson D. R. Liu C. Xu Y. Ararat M. Cabrera R. (2014). Integrated metabolomic profiling of hepatocellular carcinoma in hepatitis C cirrhosis through GC/MS and UPLC/MS-MS. Liver Int. 34, 1428–1444. 10.1111/liv.12541 24661807 PMC4169337

[B11] Guerrero-Zotano A. Mayer I. Arteaga C. (2016). PI3K/AKT/mTOR: role in breast cancer progression, drug resistance, and treatment. Cancer Metastasis Rev. 35, 515–524. 10.1007/s10555-016-9637-x 27896521

[B12] Hensley C. T. Wasti A. T. Deberardinis R. J. (2013). Glutamine and cancer: cell biology, physiology, and clinical opportunities. J. Clin. Invest. 123, 3678–3684. 10.1172/jci69600 23999442 PMC3754270

[B13] Ionkova I. Momekov G. Proksch P. (2010). Effects of cycloartane saponins from hairy roots of Astragalus membranaceus Bge, on human tumor cell targets. Fitoterapia 81, 447–451. 10.1016/j.fitote.2009.12.007 20060881

[B14] Kurma K. Manches O. Chuffart F. Sturm N. Gharzeddine K. Zhang J. (2021). DEN-induced rat model reproduces key features of human hepatocellular carcinoma. Cancers 13, 4981. 10.3390/cancers13194981 34638465 PMC8508319

[B15] Li A. Yang L. Cui T. Zhang L. Liu Y. Yan Y. (2020a). Uncovering the mechanism of Astragali Radix against nephrotic syndrome by intergrating lipidomics and network pharmacology. Phytomedicine 77, 153274. 10.1016/j.phymed.2020.153274 32771537

[B16] Li H. Ji Y. Zhang S. Gao Z. Hu C. Jiang R. (2019). Kangai injection combined with platinum-based chemotherapy for the treatment of stage III/IV non-small cell lung cancer: a meta-analysis and systematic review of 35 randomized controlled trials. J. Cancer 10, 5283–5298. 10.7150/jca.31928 31602279 PMC6775612

[B17] Li J. Xu L. Sang R. Yu Y. Ge B. Zhang X. (2018a). Immunomodulatory and anti-inflammatory effects of total flavonoids of Astragalus by regulating NF-ΚB and MAPK signalling pathways in RAW 264.7 macrophages. Pharmazie 73, 589–593. 10.1691/ph.2018.8633 30223923

[B18] Li S. Sun Y. Huang J. Wang B. Gong Y. Fang Y. (2020b). Anti-tumor effects and mechanisms of Astragalus membranaceus (AM) and its specific immunopotentiation: status and prospect. J. Ethnopharmacol. 258, 112797. 10.1016/j.jep.2020.112797 32243990

[B19] Li Y. Ye Y. Chen H. (2018b). Astragaloside IV inhibits cell migration and viability of hepatocellular carcinoma cells via suppressing long noncoding RNA ATB. Biomed. Pharmacother. 99, 134–141. 10.1016/j.biopha.2017.12.108 29331759

[B20] Li Y. Yu P. Fu W. Cai L. Yu Y. Feng Z. (2021). Ginseng-Astragalus-oxymatrine injection ameliorates cyclophosphamide-induced immunosuppression in mice and enhances the immune activity of RAW264.7 cells. J. Ethnopharmacol. 279, 114387. 10.1016/j.jep.2021.114387 34216728

[B21] Liu Y. Hong Z. Tan G. Dong X. Yang G. Zhao L. (2014). NMR and LC/MS-based global metabolomics to identify serum biomarkers differentiating hepatocellular carcinoma from liver cirrhosis. Int. J. Cancer 135, 658–668. 10.1002/ijc.28706 24382646

[B22] Michael W. David P. Gary J. Leon A. (2015). The evolving epidemiology of hepatocellular carcinoma: a global perspective. Expert Rev. Gastroenterol. Hepatol. 9, 765–779. 10.1586/17474124.2015.1028363 25827821

[B23] Nie C. He T. Zhang W. Zhang G. Ma X. (2018). Branched chain amino acids: beyond nutrition metabolism. Int. J. Mol. Sci. 19, 954. 10.3390/ijms19040954 29570613 PMC5979320

[B24] Schiliro C. Firestein B. (2021). Mechanisms of metabolic reprogramming in cancer cells supporting enhanced growth and proliferation. Cells 10, 1056. 10.3390/cells10051056 33946927 PMC8146072

[B25] Shi J. Han X. Wang J. Han G. Zhao M. Duan X. (2019). Matrine prevents the early development of hepatocellular carcinoma like lesions in rat liver. Exp. Ther. Med. 18, 2583–2591. 10.3892/etm.2019.7875 31555367 PMC6755378

[B26] Shi L. Tang X. Dang X. Wang Q. Wang X. He P. (2015). Investigating herb–herb interactions: The potential attenuated toxicity mechanism of the combined use of Glycyrrhizae radix et rhizoma (Gancao) and Sophorae flavescentis radix (Kushen). J. Ethnopharmacol. 165, 243–250. 10.1016/j.jep.2015.02.022 25701755

[B27] Song Q. Yang W. Meng Z. Wang J. (2020). Protocol for a systematic review and meta-analysis of Kang-ai injection for patients with oesophageal cancer. Medicine 99, e22148. 10.1097/MD.0000000000022148 32899102 PMC7478825

[B28] Sun P. Zhao W. Wang Q. Chen L. Sun K. Zhan Z. (2022). Chemical diversity, biological activities and Traditional uses of and important Chinese herb Sophora. Phytomedicine 100, 154054. 10.1016/j.phymed.2022.154054 35358931

[B29] Sung H. Ferlay J. Siegel R. Laversanne M. Soerjomataram I. Jemal A. (2021). Global cancer statistics 2020: GLOBOCAN estimates of incidence and mortality worldwide for 36 cancers in 185 countries. CA Cancer J. Clin. 71, 209–249. 10.3322/caac.21660 33538338

[B30] Wan Y. Li Y. Xu Z. Wu H. Xu Y. Yang M. (2018). The effect of transarterial chemoembolization in combination with kang’ai injection on patients with intermediate stage hepatocellular carcinoma: a prospective study. Integr. Cancer Ther. 17, 477–485. 10.1177/1534735417734913 29108428 PMC6041935

[B31] Wang G. Ji C. Wang C. Liu Z. Qu A. Wang H. (2021). Matrine ameliorates the inflammatory response and lipid metabolism in vascular smooth muscle cells through the NF-kappa B pathway. Exp. Ther. Med. 22, 1309. 10.3892/etm.2021.10744 34630663 PMC8461614

[B32] Wang S. Long S. Wu W. (2018). Application of traditional Chinese medicines as personalized therapy in human cancers. Am. J. Chin. Med. 46, 953–970. 10.1142/S0192415X18500507 29986595

[B33] Wangensteen K. Chang K. (2020). Multiple roles for hepatitis B and C viruses and the host in the development of hepatocellular carcinoma. Hepatology 73, 27–37. 10.1002/hep.31481 32737895 PMC7855312

[B34] Yang Y. Kim S. Seki E. (2019). Inflammation and liver cancer: molecular mechanisms and therapeutic targets. Semin. Liver Dis. 39, 26–42. 10.1055/s-0038-1676806 30809789 PMC6616367

[B35] Yu J. Li J. Cheng R. Ma Y. Wang X. Liu J. (2014). Effect of matrine on transforming growth factor β1 and hepatocyte growth factor in rat liver fibrosis model. Asian Pac. J. Trop. Med. 7, 390–393. 10.1016/s1995-7645(14)60062-6 25063067

[B36] Yu X. Chen D. Wang L. Li J. Khan H. Chen H. (2020). Wogonoside inhibits inflammatory cytokine production in lipopolysaccharide-stimulated macrophage by suppressing the activation of the JNK/c-Jun signaling pathway. Ann. Transl. Med. 8, 532. 10.21037/atm.2020.04.22 32411755 PMC7214906

[B37] Zhang H. Li X. Yu Y. Zhang X. Wang S. Yin F. (2022). Research progress of relationship between cytochrome P4503A4 and tumor chemotherapy resistance. Med. Recapitulate 28, 2122–2127. 10.3969/j.issn.1006-2084.2022.11.008

[B38] Zhang Q. Li J. Zhang Q. Cui W. Wang H. Li T. (2020). A comparative study on anti-cancer effects of Astragalus membranaceus, Sophora flavescens and gleditsia sinensis. Inf. Traditional Chin. Med. 37, 48–54. 10.19656/j.cnki.1002-2406.200010

[B39] Zhou M. Liu B. Shen J. (2022). Immunotherapy for hepatocellular carcinoma. Clin. Exp. Med. 9, 569–577. 10.1007/s10238-022-00874-5 36001163

[B40] Zhu L. Gu P. Shen H. (2019). Protective effects of berberine hydrochloride on DSS-induced ulcerative colitis in rats. Int. Immunopharmacol. 68, 242–251. 10.1016/j.intimp.2018.12.036 30743078

