# Supplementary material for: Sophora flavescens-Astragalus mongholicus herb pair in the progression of hepatitis, cirrhosis, and hepatocellular carcinoma: a possible mechanisms and relevant therapeutic substances
Source: Front Pharmacol. 2024 May 27;15:1284752. doi: 10.3389/fphar.2024.1284752 (PMC11163057; doi:10.3389/fphar.2024.1284752)
Supplement: Supplementary file 1 [file DataSheet1.pdf]

Supplementary Material

1 Supplementary Data

1.1 HPLC fingerprint analysis of SF-AM herb pair extract

The experiments were performed on a Vanquish HPLC system (Thermo Scientific). The sample was separated on a Shim-pack GIST C18 (250 mm×4.6 mm, 5 μm) at 30°C. The mobile phase consists of 10 mM of Ammonium acetate aqueous solution (adjust pH to 8.50 with ammonia, A) and acetonitrile (B). The gradient program was optimized as follows: 0-8 min, 5-6% B; 8-16 min, 6-10% B; 16-25 min, 10-13% B; 25-35 min, 13-16% B; 35-46 min, 16-18% B; 46-55 min, 18-18% B; 55-60 min, 18-20% B; 60-66 min, 20-22% B; 66-78 min, 22-33% B; 78-90 min, 33-60% B; 90-100 min, 60-60% B. The flow rate was 1.0 ml/min, and the injection volume was 5 μl.

1.2 *In vivo* components analysis of the SF-AM herb pair in the plasma of hepatitis-cirrhosis-HCC rats

The experiments were performed on an Agilent 1260 infinity liquid chromatography system (Agilent, Santa Clara, CA, USA) in tandem with a triple TOF 5600+ quadrupole time-of-flight tandem mass spectrometer (Sciex, Redwood City, CA, USA) via an electrospray ionization (ESI) interface. The sample was separated on an Kinetex XB-C18 column (100 mm × 4.6 mm, 2.6 μm) at 30°C. The mobile phase consists of water-formic acid (100:0.1, *v/v*, A) and acetonitrile (B). The gradient program was optimized as follows: 0-10 min, 8-20% B; 10-40 min, 20-60% B; 40-45 min, 60-90% B; 45-47 min, 90-8% B; 47-52 min, 8-8% B. The flow rate was 0.5 ml/min, and the injection volume was 4 μl.

The optimized parameters for the MS conditions were as follows: ion spray voltage, -4500 V (-) / 5500V (+); declustering potential, ±80 V; the turbo spray temperature, 550ºC; nebulizer gas (nitrogen), 50 psi; heater gas (nitrogen), 50 psi; and curtain gas (nitrogen), 30 psi. In the TOF MS mode, the collision energy was ± 10 V. In the TOF MS/MS mode, a typical information dependent acquisition (IDA) model was used with collision energy setting at ± 40 V. The scan ranges in TOF MS and TOF MS/MS modes were m/z 50-1250 and 50-1000, respectively.

The extracted ion chromatographic peaks of six component (sophocarpine, matrine, inermine, calycosin, calycosin-7-O-glucoside, astragaloside A) were selected for method validation. The precision of the instrument was verified by 6 consecutive injections of the same quality control (QC) sample. To investigate the method repeatability, 6 independently processed parallel samples were prepared and analyzed. The stability of the samples was examined by injection of the same QC sample separately at 0, 2, 4, 8, 12, and 24 h. The precision, repeatability, and sample stability of the developed method were all within acceptable limits.

2 Supplementary Figures and Tables

2.1 Supplementary Figures

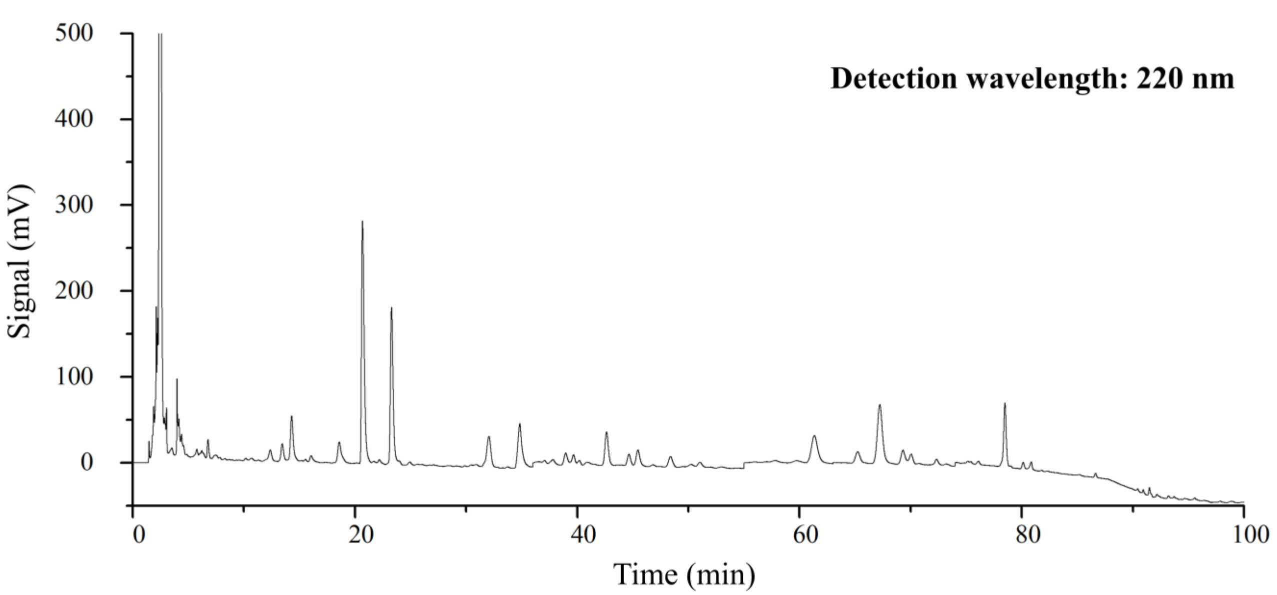
Detection wavelength: 220 nm
Signal (mV)
500
400
300
200
100
0
Time (min)
0
20
40
60
80
100

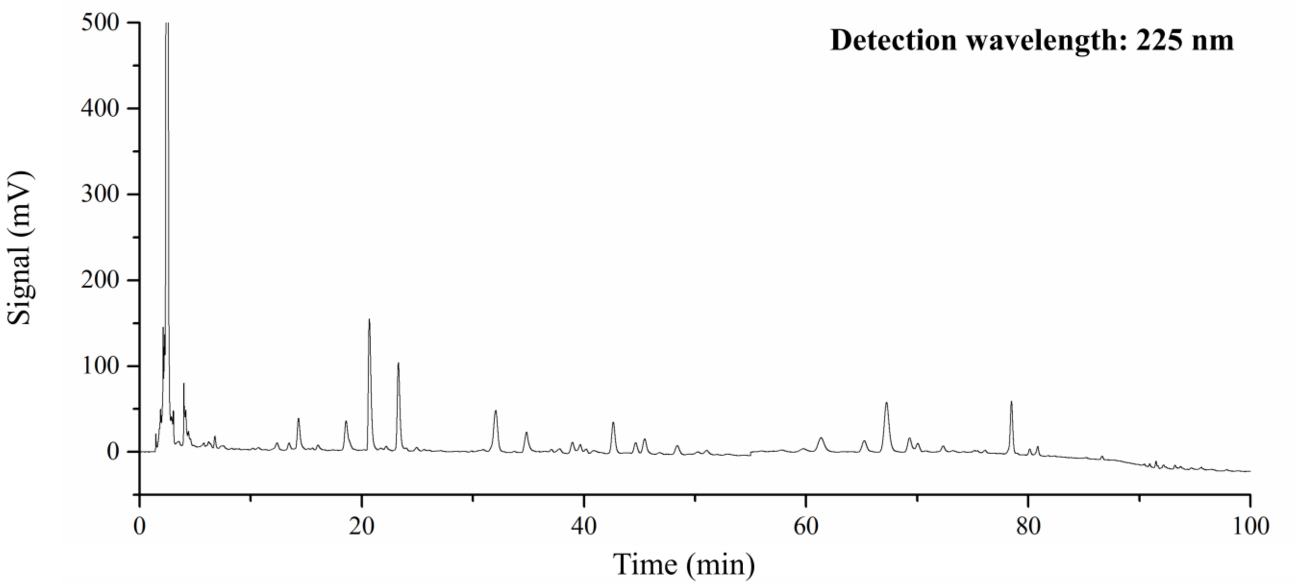
Detection wavelength: 225 nm
Signal (mV)
500
400
300
200
100
0
Time (min)
0
20
40
60
80
100

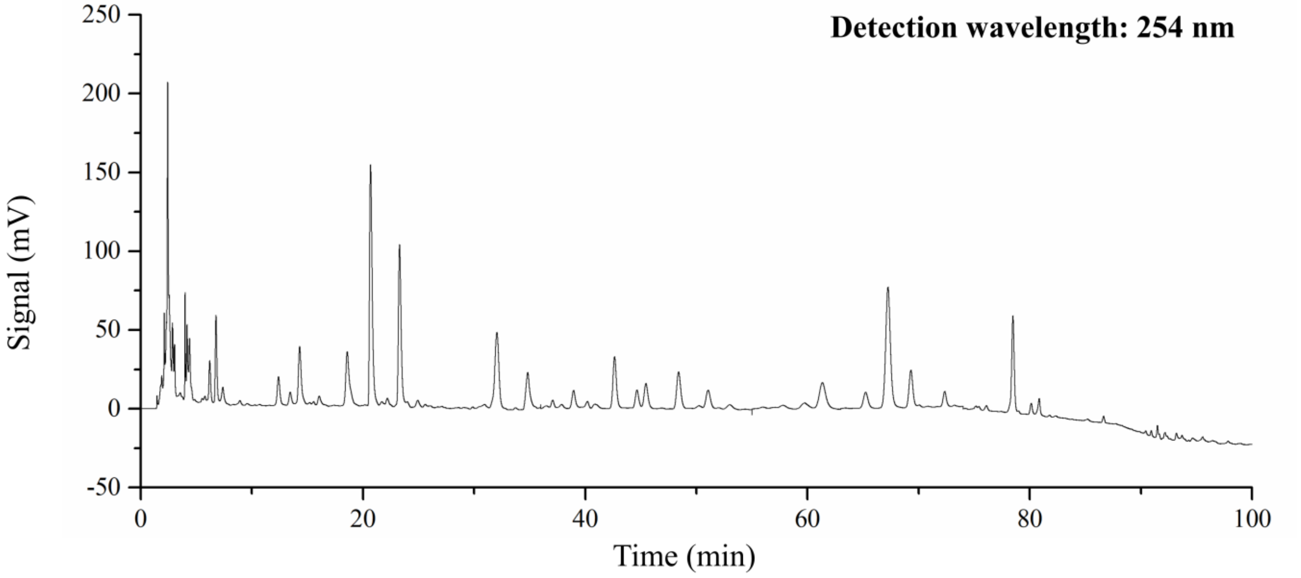
Detection wavelength: 254 nm
Signal (mV)
250
200
150
100
50
0
-50
Time (min)
0
20
40
60
80
100

Supplementary Figure 1. HPLC fingerprint analysis of AM herb pair extract (with three detection SF-wavelengths).

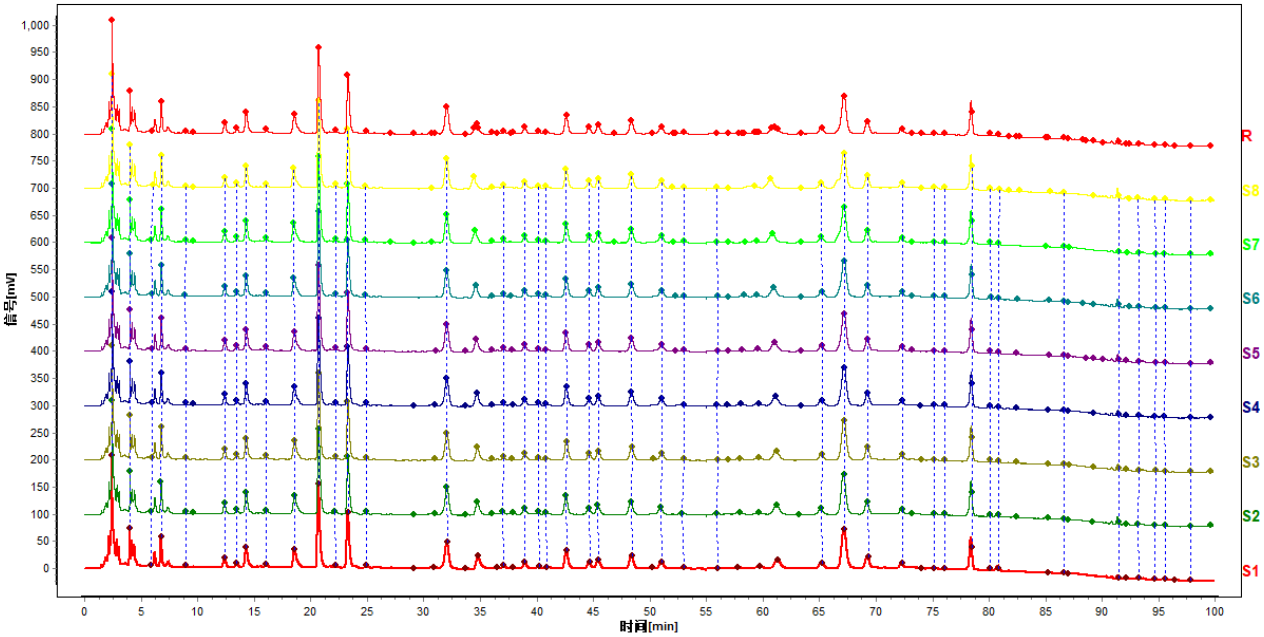

Supplementary Figure 2. HPLC fingerprint analysis of SF-AM herb pair extract extracted at different times.

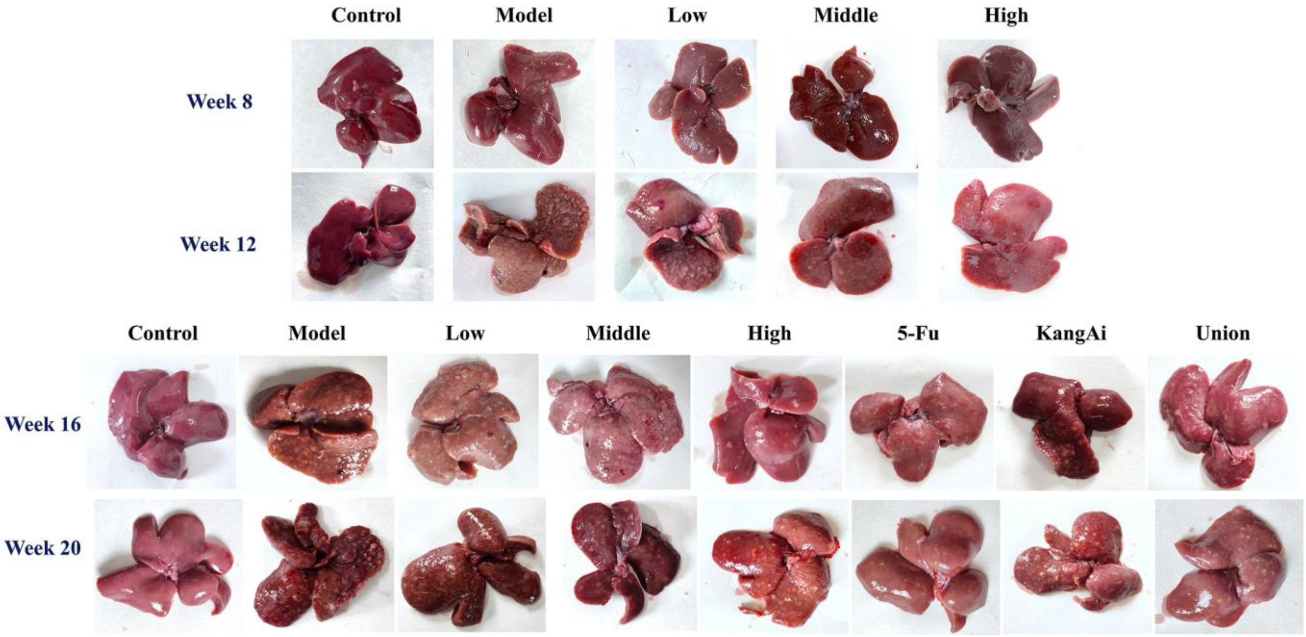

Supplementary Figure S3. Appearance of the liver in different groups of rats at hepatitis-cirrhosis-HCC stages.

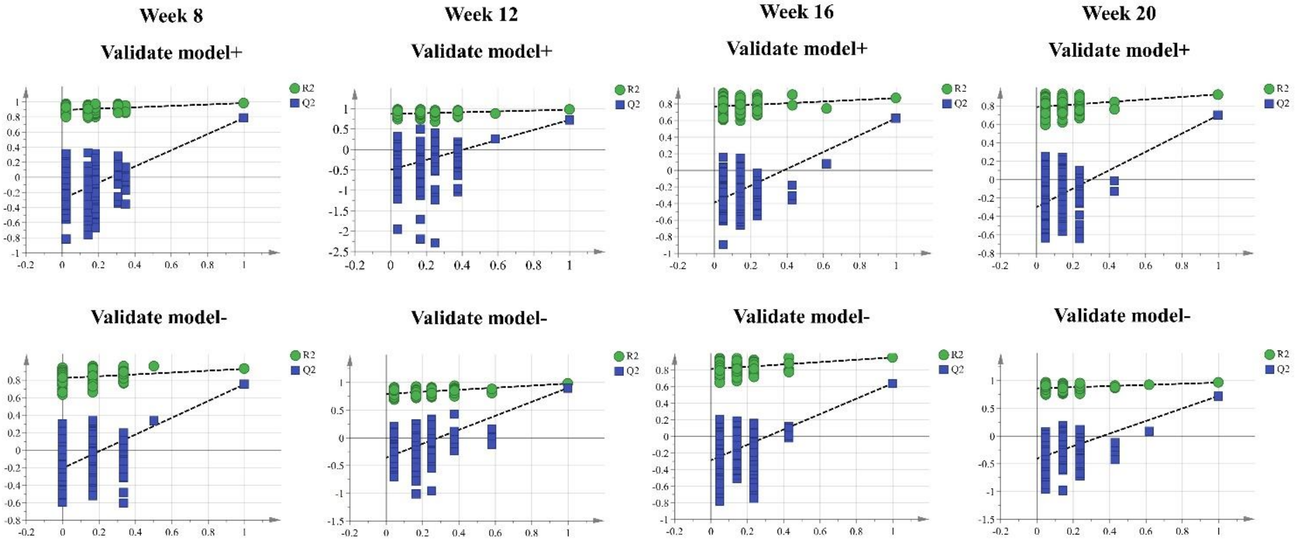

Supplementary Figure S4. Validation plots of the OPLS-DA models obtained using 200 permutation tests in rat plasma.

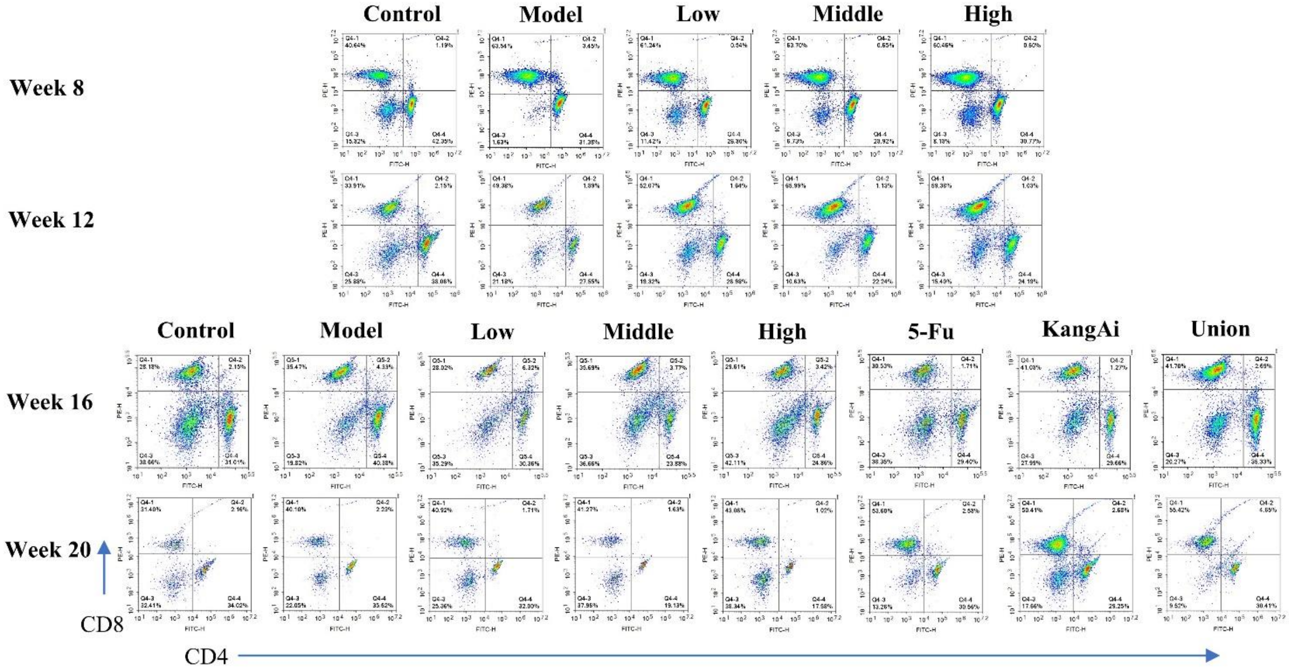

Supplementary Figure S5. Flow cytometric analysis of $CD4^+$ and $CD8^+$ T lymphocytes in rat liver at hepatitis-cirrhosis-HCC.

2.2 Supplementary Tables

Supplementary Table S1. The detail information for chemical reference substances.

Name	Manufacturer
Caulophylline	Chengdu Chroma Biotechnology Ltd.
Matrine	Chengdu Chroma Biotechnology Ltd.
Sophocarpine	Chengdu Chroma Biotechnology Ltd.
Oxysophocarpine	Chengdu Chroma Biotechnology Ltd.
Oxymatrine	Chengdu Chroma Biotechnology Ltd.
Calycosin-7-O-glucoside	National Institutes for Food and Drug control
Ononin	Chengdu Chroma Biotechnology Ltd.
Trifolirhizin	Chengdu Chroma Biotechnology Ltd.
Calycosin	Chengdu Chroma Biotechnology Ltd.
Isoastragaloside IV	Chengdu Chroma Biotechnology Ltd.
Astragaloside A	National Institutes for Food and Drug control
Formononetin	Chengdu Chroma Biotechnology Ltd.
Kurarinone	Chengdu Chroma Biotechnology Ltd.
Vexibinol	Chengdu Chroma Biotechnology Ltd.
Nuciferine	Chengdu Chroma Biotechnology Ltd.
Naringin	Chengdu Chroma Biotechnology Ltd.
Ginsenoside Re	Chengdu Chroma Biotechnology Ltd.
L-2-chlorophenylalanine	Chengdu Chroma Biotechnology Ltd.
d_4-Cortisol	Sigma-Aldrich
Heptadecanoic acid	Chengdu Chroma Biotechnology Ltd.
d_8- arachidonic acid	Sigma-Aldrich

Supplementary Table S2. Design of the SWATH experiment for ESI^+.

Experiment	MS Type	Min *m/z*	Max *m/z*	Accumulation time (ms)	CE (V)
0	SCAN	50	1000	100	30
1	SWATH	49.5	60.5	20	30
2	SWATH	59.5	71.5	20	30
3	SWATH	70.5	81.6	20	30
4	SWATH	80.6	91	20	30
5	SWATH	90	102.6	20	30
6	SWATH	101.6	107.6	20	30
7	SWATH	106.6	122.6	20	30
8	SWATH	121.6	133.5	20	30
9	SWATH	132.5	142.5	20	30
10	SWATH	141.5	149.5	20	30
11	SWATH	148.5	157.6	20	30
12	SWATH	156.6	162.6	20	30
13	SWATH	161.6	178.5	20	30
14	SWATH	177.5	198.1	20	30
15	SWATH	197.1	206.5	20	30
16	SWATH	205.5	220.5	20	30
17	SWATH	219.5	245.7	20	30

18	SWATH	244.7	263.5	20	30
19	SWATH	262.5	279.7	20	30
20	SWATH	278.7	286.7	20	30
21	SWATH	285.7	301.6	20	30
22	SWATH	300.6	307.6	20	30
23	SWATH	306.6	321.6	20	30
24	SWATH	320.6	340.8	20	30
25	SWATH	339.8	357.8	20	30
26	SWATH	356.8	371.8	20	30
27	SWATH	370.8	384.7	20	30
28	SWATH	383.7	404.8	20	30
29	SWATH	403.8	426.8	20	30
30	SWATH	425.8	449.1	20	30
31	SWATH	448.1	472.8	20	30
32	SWATH	471.8	489.3	20	30
33	SWATH	488.3	509	20	30
34	SWATH	508	524.3	20	30
35	SWATH	523.3	544.8	20	30
36	SWATH	543.8	569.7	20	30
37	SWATH	568.7	604.9	20	30
38	SWATH	603.9	693.6	20	30
39	SWATH	692.6	783.2	20	30
40	SWATH	782.2	1000.5	20	30

Supplementary Table S3. Design of the SWATH experiment for ESI^-.

Experiment	MS Type	Min *m/z*	Max *m/z*	Accumulation time (ms)	CE (V)
0	SCAN	50	1000	100	-30
1	SWATH	49.5	130.5	40	-30
2	SWATH	129.5	165.4	40	-30
3	SWATH	164.4	192.4	40	-30
4	SWATH	191.4	229.4	40	-30
5	SWATH	228.4	250.5	40	-30
6	SWATH	249.5	277.5	40	-30
7	SWATH	276.5	297.4	40	-30
8	SWATH	296.4	315.5	40	-30
9	SWATH	314.5	332.6	40	-30
10	SWATH	331.6	355.5	40	-30
11	SWATH	354.5	376.4	40	-30
12	SWATH	375.4	395.8	40	-30
13	SWATH	394.8	425.4	40	-30
14	SWATH	424.4	451.7	40	-30
15	SWATH	450.7	475.8	40	-30
16	SWATH	474.8	511.4	40	-30
17	SWATH	510.4	549.6	40	-30
18	SWATH	548.6	600.8	40	-30
19	SWATH	599.8	701.8	40	-30
20	SWATH	700.8	1000.5	40	-30

Supplementary Table S4. MS-DIAL parameters setting.

Item	Postive	Negative

Addduct	Addcut type	$[M+H]^+$, $[M+NH_4]^+$, $[M+Na]^+$	$[M-H]^-$, $[M+FA-H]^-$
Data collection	MS1 tolerance	0.01 Da	0.01 Da
	MS2 tolerance	0.05 Da	0.05 Da
Peak detection	Minimum peak height	1000 cps	1000 cps
	Mass slice width	0.1 Da	0.1 Da
	Smoothing level	3	3
	Minimum peak width	5	5
MS2Dec	Sigma window value	0.5	0.5
	MS/MS abundance cut off	5 Amplitude	5 Amplitude
Identification	Identification score cut off	80%	80%
Alignment	Retention time tolerance	0.05 min	0.05 min
	MS1 tolerance	0.015 Da	0.015Da

Supplementary Table S5. Precision, repeatability and stability in the method validation of the nontargeted metabolomic study in positive mode.

m/z_t_R	Precision (RSD %)		Repeatability (RSD %)		Stability (RSD %)	
	t_R	Intensity	t_R	Intensity	t_R	Intensity
112.0499_3.66	1.89	8.69	1.58	8.99	1.56	7.31
212.0914_13.56	0.72	7.94	0.92	7.89	0.27	8.51
311.2683_13.92	0.81	13.15	0.61	7.99	0.28	9.06
407.1343_7.13	1.26	11.77	1.55	12.13	1.24	6.51
646.7573_2.32	1.27	3.89	1.91	7.74	1.50	11.15
880.7400_2.30	1.00	8.71	1.25	5.10	1.47	8.36

Supplementary Table S6. Precision, repeatability and stability in the method validation of the nontargeted metabolomic study in negative mode.

m/z_t_R	Precision (RSD %)		Repeatability (RSD %)		Stability (RSD %)	
	t_R	Intensity	t_R	Intensity	t_R	Intensity
112.0512_2.53	1.17	7.28	1.57	10.62	0.16	9.12
213.1109_16.92	0.13	9.25	0.61	14.08	0.18	6.65
332.1168_13.28	0.06	11.31	0.82	8.40	1.30	13.02
413.1186_24.30	0.25	6.82	0.54	8.93	0.34	5.03
645.1493_12.54	1.13	13.06	0.38	12.47	0.12	8.08
814.4772_14.80	0.07	11.26	0.45	11.81	0.13	9.55

Supplementary Table S7. The detailed information of differential metabolites in plasma from rats with different stages of HCC compared to healthy rats.

Note: The red font indicates that metabolite levels increased in the model group and decreased in blue.

NO.	t_R (min)	*m/z*	Identification	Adduct	Formula	Week and tendency
1	2.01	146.1653	Spermidine	$[M+H]^+$	C7H19N3	16
2	2.02	116.0702	L-Proline	$[M+H]^+$	C5H9NO2	12、20
3	2.03	130.0864	Pipecolic acid	$[M+H]^+$	C6H11NO2	16
4	2.03	145.0977	L-Lysine	$[M-H]^-$	C6H14N2O2	12、16、20
5	2.05	161.0947	D-Alanyl-D-alanine	$[M+H]^+$	C6H12N2O3	16
6	2.15	169.0945	Pyridoxamine	$[M+H]^+$	C8H12N2O2	16
7	2.18	133.0981	Ornithine	$[M+H]^+$	C5H12N2O2	20
8	2.33	154.0625	L-Histidine	$[M-H]^-$	C6H9N3O2	8、12、16、20
9	2.34	104.0336	L-Serine	$[M-H]^-$	C3H7NO3	12、16、20
10	2.34	173.1015	L-Arginine	$[M-H]^-$	C6H14N4O2	8、20
11	2.36	124.0063	Taurine	$[M-H]^-$	C2H7NO3S	20
12	2.36	132.0281	D-Aspartic acid	$[M-H]^-$	C4H7NO4	16
13	2.37	147.0766	L-Glutamine	$[M+H]^+$	C5H10N2O3	12、16
14	2.38	120.0641	L-Threonine	$[M+H]^+$	C4H9NO3	8、12
15	2.39	184.0757	Phosphorylcholine	$[M+H]^+$	C5H15NO4P	8
16	2.39	241.0312	Cystine	$[M+Na]^+$	C6H12N2O4S2	16
17	2.41	97.0282	Propionic acid	$[M+H]^+$	C3H6O2	8、12、16
18	2.41	263.0098	L-Cystine	$[M+H]^+$	C6H12N2O4S2	20
19	2.41	146.0454	L-Glutamic acid	$[M-H]^-$	C5H9NO4	8、12、16、20
20	2.41	195.0494	Gluconic acid	$[M-H]^-$	C6H12O7	16、20
21	2.42	193.0347	D-Glucuronic acid	$[M-H]^-$	C6H10O7	12、16、20

22	2.44	162.1113	L-Carnitine	$[M+H]^+$	C7H15NO3	8、12、16、20
23	2.45	198.0831	Citrulline	$[M+H]^+$	C6H13N3O3	12、16、20
24	2.45	165.0396	3-Methylxanthine	$[M-H]^-$	C6H6N4O2	8、12
25	2.46	131.0809	D-Ornithine	$[M-H]^-$	C5H12N2O2	16、20
26	2.46	196.073	N-Acetylhistidine	$[M-H]^-$	C8H11N3O3	12、20
27	2.51	93.0543	Glycerol	$[M+H]^+$	C3H8O3	8、12、20
28	2.52	135.0295	Threonic acid	$[M-H]^-$	C4H8O5	8、12、16、20
29	2.54	61.0408	Urea	$[M+H]^+$	CH4N2O	8、20
30	2.61	102.054	Gamma-Aminobutyric acid	$[M-H]^-$	C4H9NO2	12、16
31	2.62	140.0111	O-Phosphoethanolamine	$[M-H]^-$	C2H8NO4P	16
32	2.64	190.0711	N-Acetylglutamic acid	$[M+H]^+$	C7H11NO5	20
33	2.64	116.0737	L-Valine	$[M-H]^-$	C5H11NO2	12、16
34	2.64	149.0447	L-Arabinose	$[M-H]^-$	C5H10O5	12、16、20
35	2.65	276.1176	Glutamylglutamine	$[M+H]^+$	C10H17N3O6	16
36	2.66	249.108	Glutamylthreonine	$[M+H]^+$	C9H16N2O6	8、12、16、20
37	2.67	112.0498	Cytosine	$[M+H]^+$	C4H5N3O	12
38	2.71	308.0905	Glutathione	$[M+H]^+$	C10H17N3O6S	8、12、16、20
39	2.75	119.0356	Purine	$[M-H]^-$	C5H4N4	20
40	2.90	87.0088	Pyruvic acid	$[M-H]^-$	C3H4O3	12、16、20
41	2.92	75.0078	Glycolic acid	$[M-H]^-$	C2H4O3	8、12、16、20
42	2.92	148.0437	L-Methionine	$[M-H]^-$	C5H11NO2S	8、12、16、20
43	2.96	104.1057	Choline	$[M+H]^+$	C5H14NO	16、20
44	3.06	91.0404	L-Lactic acid	$[M+H]^+$	C3H6O3	20
45	3.07	89.0251	Glyceraldehyde	$[M-H]^-$	C3H6O3	20

46	3.09	103.0029	Malonate	[M-H]-	C3H4O4	8、12、20
47	3.21	204.1226	L-Acetylcarnitine	[M]+	C9H18NO4	12、20
48	3.23	101.0233	2-Ketobutyric acid	[M-H]-	C4H6O3	8、12、16
49	3.23	145.0143	Oxoglutaric acid	[M-H]-	C5H6O5	12、16、20
50	3.24	189.1224	Glycylleucine	[M+H]+	C8H16N2O3	12、16、20
51	3.30	130.0875	L-Leucine	[M-H]-	C6H13NO2	12、16
52	3.44	88.0418	L-Alanine	[M-H]-	C3H7NO2	8、12、16、20
53	3.57	245.075	Uridine	[M+H]+	C9H12N2O6	16
54	3.64	203.1379	Ala-Ile	[M+H]+	C9H18N2O3	16
55	3.68	139.0012	Fumaric acid	[M+H]+	C4H4O4	16、20
56	3.69	215.0152	Citric acid	[M+Na]+	C6H8O7	12、16、20
57	3.78	89.0248	Hydroxypropionic acid	[M-H]-	C3H6O3	20
58	3.84	149.0173	Maltol	[M+H]+	C6H6O3	8、12、20
59	3.87	103.0401	3-Hydroxybutyric acid	[M-H]-	C4H8O3	8、12、16、20
60	4.14	282.1185	1-Methyladenosine	[M+H]+	C11H15N5O4	16
61	4.24	118.0634	Indole	[M+H]+	C8H7N	12、16、20
62	4.27	123.0422	Benzoic acid	[M+H]+	C7H6O2	8、12、16、20
63	4.30	180.0646	L-Tyrosine	[M-H]-	C9H11NO3	8、12、16、20
64	4.96	126.0655	5-Methylcytosine	[M+H]+	C5H7N3O	12、16、20
65	5.14	175.0244	Ascorbic acid	[M-H]-	C6H7O6	12、16
66	5.76	279.0999	gamma-Glutamylmethionine	[M+H]+	C10H17N3O6	8、12、16、20
67	5.83	207.0784	L-Kynurenine	[M-H]-	C10H12N2O3	8、12、16、20
68	6.06	144.0674	4-Acetamidobutanoic acid	[M-H]-	C6H11NO3	8、12、20

69	7.54	137.045	Hypoxanthine	$[M+H]^+$	C5H4N4O	20
70	7.87	220.1174	Pantothenic acid	$[M+H]^+$	C9H17NO5	12、16、20
71	8.12	204.029	Xanthurenic acid	$[M-H]^-$	C10H7NO4	20
72	8.17	261.1422	gamma-Glutamylleucine	$[M+H]^+$	C11H20N2O5	8、12、16、20
73	8.61	311.1249	gamma-Glutamyltyrosine	$[M+H]^+$	C14H18N2O6	8、12、16、20
74	8.87	173.0451	D-Xylose	$[M+Na]^+$	C5H10O5	16、20
75	8.88	151.0604	D-Xylulose	$[M+H]^+$	C5H10O5	12、16、20
76	9.02	203.0819	L-Tryptophan	$[M-H]^-$	C11H12N2O2	20
77	9.09	170.0599	Pyridoxine	$[M+Na]^+$	C8H11NO3	16
78	9.12	89.0582	Butyric acid	$[M+H]^+$	C4H8O2	16
79	9.16	241.083	Thymidine	$[M-H]^-$	C10H14N2O5	16
80	9.27	127.0486	Thymine	$[M+H]^+$	C5H6N2O2	8、12、16
81	9.62	284.0899	N4-Acetylcytidine	$[M-H]^-$	C11H15N3O6	16
82	13.68	204.064	Indolelactic acid	$[M-H]^-$	C11H11NO3	12、16、20
83	14.53	137.0223	4-Hydroxybenzoic acid	$[M-H]^-$	C7H6O3	12、16
84	16.56	514.2806	Taurocholic acid	$[M-H]^-$	C26H45NO7S	8、12、16、20
85	17.12	929.6063	Glycocholic acid	$[M-H]^-$	C26H43NO6	12、16、20
86	17.82	321.0406	5-Thymidylic acid	$[M-H]^-$	C10H15N2O8P	16
87	18.79	500.3002	Taurodeoxycholic acid	$[M+H]^+$	C26H45NO6S	20
88	19.23	498.2829	Taurochenodesoxycholic acid	$[M-H]^-$	C26H45NO6S	12、16、20
89	20.05	901.5485	PI (18:0/12-HETE)	$[M-H]^-$	C47H83O14P	8、12、16、20
90	20.43	393.3001	Deoxycholic acid	$[M+H]^+$	C24H40O4	12、20
91	20.43	858.5894	PC (18:1/13-HODE)	$[M-H]^-$	C44H82NO9P	8、12、20

92	21.12	373.2709	Cholic acid	$[M+H]^+$	C24H40O5	12、16、20
93	21.72	359.2932	Lithocholic acid	$[M+H]^+$	C24H40O3	12、20
94	21.79	882.587	PC (18:0/12-HEPE)	$[M-H]^-$	C46H82NO9P	20
95	21.87	375.2867	Chenodeoxycholic acid	$[M+H2O+H]^+$	C24H40O4	12、20
96	21.91	452.1775	LPE (16:0)	$[M-H]^-$	C21H44NO7P	8、12、16、20
97	21.96	255.2294	Palmitic Acid	$[M-H]^-$	C16H32O2	8、12
98	22.05	303.2324	Arachidonic Acid	$[M-H]^-$	C20H32O2	12
99	22.21	424.3416	Linoleyl carnitine	$[M+H]^+$	C25H45NO4	8
100	22.50	520.3378	LPC (18:2)	$[M+H]^+$	C26H50NO7P	12
101	22.77	400.3394	L-Palmitoylcarnitine	$[M+H]^+$	C23H45NO4	12、20
102	23.07	480.3085	LPE (18:1)	$[M+H]^+$	C23H46NO7P	16、20
103	23.47	884.6055	PC (18:0/18-HETE)	$[M-H]^-$	C46H84NO9P	8、20
104	23.94	522.3551	LPC (18:1)	$[M+H]^+$	C26H52NO7P	8、12、16、20
105	24.18	506.3248	LPE (20:0)	$[M-H]^-$	C25H52NO7P	12、16、20
106	25.03	552.3633	LPC (18:0)	$[M-H]^-$	C26H54NO7P	12
107	29.05	524.2745	LPE (22:0)	$[M-H]^-$	C27H56NO7P	16、20
108	29.99	348.2911	Palmitoylethanolamide	$[M+NH4]^+$	C18H37NO2	8、20
109	31.53	243.2333	Pentadecanoic acid	$[M+H]^+$	C15H30O2	20
110	31.58	319.2259	12-HETE	$[M-H]^-$	C20H32O3	16

Supplementary Table S8. Changes of biomarkers after given SF-AM herb pair in nontargeted metabolomics (Normalized data). **p*<0.05, ***p*<0.01, compared with model group.

Biomarkers	Control	Model	Low	Middle	High	Week

2-Ketobutyric acid	$7.579\times10^{-1}\pm2.052\times10^{-1}$**	$1.497\pm3.78\times10^{-1}$	$1.288\pm3.87\times10^{-1}$	$1.392\pm4.08\times10^{-1}$	$1.020\pm1.23\times10^{-1}$*	8
Malonate	$1.204\times10^{-1}\pm3.95\times10^{-2}$*	$6.483\times10^{-2}\pm2.225\times10^{-2}$	$5.354\times10^{-2}\pm1.848\times10^{-2}$	$7.428\times10^{-2}\pm3.168\times10^{-2}$	$1.308\times10^{-1}\pm4.97\times10^{-2}$*	8
L-Glutamic acid	$3.586\pm7.21\times10^{-1}$**	6.568 ± 1.463	4.674 ± 1.272*	5.326 ± 1.625	$4.304\pm9.32\times10^{-1}$*	8
3-Methylxanthine	$5.435\times10^{-1}\pm9.14\times10^{-2}$**	$3.134\times10^{-1}\pm1.469\times10^{-1}$	$2.921\times10^{-1}\pm9.22\times10^{-2}$	$5.256\times10^{-1}\pm1.295\times10^{-1}$*	$6.209\times10^{-1}\pm1.229\times10^{-1}$**	8
Threonic acid	$6.891\pm6.90\times10^{-1}$**	2.535 ± 1.228	$2.480\pm7.06\times10^{-1}$	3.797 ± 1.557	5.440 ± 1.398**	8
Benzoic acid	5.430 ± 1.167**	$1.126\times10^{1}\pm1.69$	$1.195\times10^{1}\pm3.70$	$1.070\times10^{1}\pm2.11$	$9.12\pm9.0\times10^{-1}$*	8
Palmitoylethanolamide	$3.588\times10^{-1}\pm4.40\times10^{-2}$**	$1.402\pm6.26\times10^{-1}$	$2.163\times10^{-1}\pm7.19\times10^{-2}$**	$3.175\times10^{-1}\pm1.132\times10^{-1}$*	$5.498\times10^{-1}\pm3.067\times10^{-1}$*	8
Linoleyl carnitine	$4.853\times10^{-1}\pm1.391\times10^{-1}$*	$7.713\times10^{-1}\pm2.362\times10^{-1}$	$7.367\times10^{-1}\pm2.163\times10^{-1}$	$4.764\times10^{-1}\pm1.329\times10^{-1}$*	$4.600\times10^{-1}\pm1.126\times10^{-1}$*	8
L-Threonine	$3.656\pm5.01\times10^{-1}$*	$2.869\pm2.81\times10^{-1}$	$3.445\pm7.90\times10^{-1}$	3.837 ± 1.117*	$3.241\pm4.31\times10^{-1}$	8
Glycolic acid	$2.164\times10^{-1}\pm2.56\times10^{-2}$**	$7.212\times10^{-2}\pm1.288\times10^{-2}$	$9.98\times10^{-2}\pm2.47\times10^{-2}$*	$1.007\times10^{-1}\pm3.30\times10^{-2}$	$1.146\times10^{-1}\pm3.01\times10^{-2}$*	12
Pyruvic acid	$2.426\pm3.20\times10^{-1}$**	$3.447\pm2.98\times10^{-1}$	$2.838\pm4.00\times10^{-1}$*	$2.900\pm5.53\times10^{-1}$	$3.498\pm8.46\times10^{-1}$	12
L-Alanine	$5.933\times10^{-2}\pm2.857\times10^{-2}$**	$2.637\times10^{-1}\pm4.52\times10^{-2}$	$1.971\times10^{-1}\pm4.96\times10^{-2}$	$2.146\times10^{-1}\pm2.83\times10^{-2}$	$2.095\times10^{-1}\pm2.28\times10^{-2}$*	12
Malonate	$1.229\times10^{-1}\pm2.46\times10^{-2}$**	$2.646\times10^{-2}\pm1.120\times10^{-2}$	$3.982\times10^{-2}\pm1.499\times10^{-2}$	$5.970\times10^{-2}\pm1.761\times10^{-2}$**	$9.23\times10^{-2}\pm6.73\times10^{-2}$*	12
Threonic acid	5.328 ± 1.521**	$1.570\pm4.40\times10^{-1}$	$1.813\pm6.64\times10^{-1}$	$2.346\pm3.50\times10^{-1}$**	3.427 ± 1.280**	12
L-Methionine	$6.095\times10^{-1}\pm9.22\times10^{-2}$*	$7.569\times10^{-1}\pm1.300\times10^{-1}$	$7.591\times10^{-1}\pm1.382\times10^{-1}$	$6.145\times10^{-1}\pm6.23\times10^{-2}$*	$6.184\times10^{-1}\pm1.263\times10^{-1}$	12
L-Arabinose	$2.145\times10^{-1}\pm4.63\times10^{-2}$**	$4.238\times10^{-1}\pm8.23\times10^{-2}$	$2.985\times10^{-1}\pm5.57\times10^{-2}$*	$4.384\times10^{-1}\pm4.00\times10^{-2}$	$4.802\times10^{-1}\pm6.21\times10^{-2}$	12
3-Methylxanthine	$6.376\times10^{-1}\pm1.469\times10^{-1}$**	$2.993\times10^{-1}\pm8.33\times10^{-2}$	$4.807\times10^{-1}\pm1.517\times10^{-1}$*	$6.632\times10^{-1}\pm2.003\times10^{-1}$**	$8.21\times10^{-1}\pm1.66\times10^{-1}$**	12
L-Tyrosine	$1.522\times10^{1}\pm7.9\times10^{-1}$**	$3.274\times10^{1}\pm4.17$	$2.342\times10^{1}\pm4.08$**	$2.459\times10^{1}\pm4.03$*	$2.453\times10^{1}\pm2.81$**	12
Propionic acid	$1.135\times10^{-1}\pm1.76\times10^{-2}$*	$9.01\times10^{-2}\pm1.13\times10^{-2}$	$9.19\times10^{-2}\pm1.32\times10^{-2}$	$1.106\times10^{-1}\pm1.36\times10^{-2}$*	$8.83\times10^{-2}\pm1.43\times10^{-2}$	12
Cytosine	$9.65\times10^{-2}\pm1.29\times10^{-2}$**	$5.316\times10^{-2}\pm1.906\times10^{-2}$	$8.75\times10^{-2}\pm1.59\times10^{-2}$**	$5.631\times10^{-2}\pm2.547\times10^{-2}$	$5.341\times10^{-2}\pm2.312\times10^{-2}$	12
Benzoic acid	$4.294\times10^{-1}\pm2.99\times10^{-2}$**	$8.38\times10^{-1}\pm1.80\times10^{-1}$	$6.074\times10^{-1}\pm7.44\times10^{-2}$*	$7.139\times10^{-1}\pm1.604\times10^{-1}$	$6.524\times10^{-1}\pm1.106\times10^{-1}$	12
L-Glutamine	$3.054\pm2.73\times10^{-1}$**	$2.624\pm1.70\times10^{-1}$	$2.650\pm3.03\times10^{-1}$	$3.192\pm2.46\times10^{-1}$**	$3.193\pm4.62\times10^{-1}$*	12
Pantothenic acid	$3.517\times10^{-1}\pm5.48\times10^{-2}$**	$5.194\times10^{-1}\pm5.11\times10^{-2}$	$4.555\times10^{-1}\pm1.241\times10^{-1}$	$4.223\times10^{-1}\pm1.049\times10^{-1}$	$3.930\times10^{-1}\pm9.73\times10^{-2}$*	12
Glutathione	$1.344\times10^{-2}\pm1.32\times10^{-3}$**	$4.988\times10^{-3}\pm1.808\times10^{-3}$	$1.428\times10^{-2}\pm5.82\times10^{-3}$**	$8.35\times10^{-3}\pm4.66\times10^{-3}$	$1.163\times10^{-2}\pm7.21\times10^{-3}$	12

Supplementary Table S9. Changes of biomarkers after given SF-AM herb pair in nontargeted metabolomic (Normalized data). $^{*}p<0.05$, $^{**}p<0.01$, compared with model group.

Biomarker	Control	Model	Low	Middle	High	5-Fu	KangAi	Union	Week
Glycolic acid	$2.204\times10^{-1}\pm2.88\times10^{-2}$**	$1.512\times10^{-1}\pm3.41\times10^{-2}$	$2.004\times10^{-1}\pm2.86\times10^{-2}$*	$2.146\times10^{-1}\pm3.08\times10^{-2}$**	$1.591\times10^{-1}\pm3.43\times10^{-2}$	$1.338\times10^{-1}\pm1.52\times10^{-2}$	$1.923\times10^{-1}\pm3.65\times10^{-2}$	$1.475\times10^{-1}\pm2.39\times10^{-2}$	16
2-Ketobutyric acid	$6.421\times10^{-1}\pm1.528\times10^{-1}$**	$1.132\pm2.78\times10^{-1}$	$1.048\pm3.71\times10^{-1}$	$1.048\pm2.66\times10^{-1}$	$6.438\times10^{-1}\pm2.236\times10^{-1}$**	$9.19\times10^{-1}\pm2.54\times10^{-1}$	$1.437\pm7.24\times10^{-1}$	$9.56\times10^{-1}\pm3.75\times10^{-1}$	16
Gamma-Aminobutyric acid	$8.44\times10^{-2}\pm4.80\times10^{-2}$**	$1.756\times10^{-1}\pm3.45\times10^{-2}$	$1.462\times10^{-1}\pm5.62\times10^{-2}$	$1.394\times10^{-1}\pm5.86\times10^{-2}$	$9.60\times10^{-2}\pm3.56\times10^{-2}$**	$1.492\times10^{-1}\pm5.74\times10^{-2}$	$1.748\times10^{-1}\pm8.55\times10^{-2}$	$1.523\times10^{-1}\pm9.64\times10^{-2}$	16
L-Serine	$3.028\times10^{-1}\pm3.31\times10^{-2}$**	$3.915\times10^{-1}\pm5.50\times10^{-2}$	$3.885\times10^{-1}\pm9.32\times10^{-2}$	$3.475\times10^{-1}\pm5.18\times10^{-2}$	$3.106\times10^{-1}\pm3.81\times10^{-2}$*	$3.536\times10^{-1}\pm7.72\times10^{-2}$	$4.354\times10^{-1}\pm9.68\times10^{-2}$	$3.446\times10^{-1}\pm3.79\times10^{-2}$	16
L-Leucine	7.747 ± 1.352*	$6.093\pm8.05\times10^{-1}$	6.295 ± 1.310	7.710 ± 1.472*	$5.782\pm9.17\times10^{-1}$	6.507 ± 1.863	7.837 ± 1.388*	$6.266\pm1.233\times10^{-1}$	16
Ornithine	$1.676\pm4.46\times10^{-1}$**	$2.559\pm4.34\times10^{-1}$	$2.196\pm4.16\times10^{-1}$	$1.714\pm3.52\times10^{-1}$**	$1.673\pm2.41\times10^{-1}$**	$2.311\pm4.09\times10^{-1}$	$2.777\pm6.95\times10^{-1}$	$2.495\pm5.94\times10^{-1}$	16
D-Aspartic acid	$3.284\times10^{-1}\pm1.641\times10^{-1}$*	$5.290\times10^{-1}\pm9.73\times10^{-2}$	$2.948\times10^{-1}\pm8.33\times10^{-2}$**	$2.204\times10^{-1}\pm4.15\times10^{-2}$**	$3.098\times10^{-1}\pm1.214\times10^{-1}$**	$4.089\times10^{-1}\pm2.350\times10^{-1}$	$6.105\times10^{-1}\pm2.601\times10^{-1}$	$3.615\times10^{-1}\pm2.142\times10^{-1}$	16
O-Phosphoethanolamine	$1.582\times10^{-1}\pm4.09\times10^{-2}$**	$2.353\times10^{-1}\pm3.47\times10^{-2}$	$1.963\times10^{-1}\pm3.03\times10^{-2}$	$1.897\times10^{-1}\pm2.51\times10^{-2}$*	$1.931\times10^{-1}\pm5.26\times10^{-2}$	$1.929\times10^{-1}\pm5.04\times10^{-2}$	$2.129\times10^{-1}\pm4.54\times10^{-2}$	$2.125\times10^{-1}\pm4.90\times10^{-2}$	16
L-Lysine	$4.414\times10^{-1}\pm5.23\times10^{-2}$**	$5.878\times10^{-1}\pm4.20\times10^{-2}$	$5.301\times10^{-1}\pm1.063\times10^{-1}$	$5.476\times10^{-1}\pm1.578\times10^{-1}$	$4.511\times10^{-1}\pm9.32\times10^{-2}$**	$5.242\times10^{-1}\pm7.66\times10^{-2}$	$6.358\times10^{-1}\pm1.965\times10^{-1}$	$5.141\times10^{-1}\pm8.75\times10^{-2}$	16
L-Glutamic acid	$3.363\pm8.55\times10^{-1}$**	7.045 ± 1.061	4.989 ± 1.200*	$4.840\pm6.62\times10^{-1}$**	3.998 ± 1.201**	5.287 ± 1.928	7.896 ± 3.514	5.590 ± 2.934	16

L-Arabinose	$2.460\times10^{-1}\pm5.62\times10^{-2}$**	$5.088\times10^{-1}\pm1.129\times10^{-1}$	$4.777\times10^{-1}\pm8.37\times10^{-2}$	$3.572\times10^{-1}\pm4.56\times10^{-2}$*	$4.439\times10^{-1}\pm5.16\times10^{-2}$	$4.216\times10^{-1}\pm6.63\times10^{-2}$	$5.835\times10^{-1}\pm3.570\times10^{-1}$	$4.153\times10^{-1}\pm6.86\times10^{-2}$	16
L-Histidine	$1.173\pm1.34\times10^{-1}$*	$1.505\pm2.45\times10^{-1}$	$1.370\pm2.02\times10^{-1}$	$1.152\pm1.52\times10^{-1}$*	$1.277\pm2.56\times10^{-1}$	$1.202\pm4.64\times10^{-1}$	$1.762\pm4.28\times10^{-1}$	$1.319\pm2.78\times10^{-1}$	16
L-Tyrosine	$1.672\times10^{1}\pm1.99$*	$2.004\times10^{1}\pm2.46$	$2.102\times10^{1}\pm3.25$	$2.061\times10^{1}\pm2.35$	$1.696\times10^{1}\pm2.13$*	$1.902\times10^{1}\pm2.45$	$2.220\times10^{1}\pm4.51$	$1.789\times10^{1}\pm1.34$	16
Gluconic acid	$8.85\times10^{-1}\pm4.32\times10^{-1}$*	$1.390\pm2.99\times10^{-1}$	$1.142\pm3.13\times10^{-1}$	$1.048\pm1.94\times10^{-1}$*	$1.997\pm5.42\times10^{-1}$	$1.026\pm2.59\times10^{-1}$*	$1.050\pm3.57\times10^{-1}$	$1.312\pm4.96\times10^{-1}$	16
Propionic acid	$7.831\times10^{-2}\pm8.55\times10^{-3}$*	$6.291\times10^{-2}\pm9.52\times10^{-3}$	$7.912\times10^{-2}\pm9.21\times10^{-3}$*	$8.84\times10^{-2}\pm3.7\times10^{-2}$**	$1.061\times10^{-1}\pm2.15\times10^{-2}$**	$7.453\times10^{-2}\pm3.83\times10^{-3}$*	$9.85\times10^{-2}\pm1.53\times10^{-2}$**	$9.53\times10^{-2}\pm1.45\times10^{-2}$**	16
Pyruvic acid	$2.208\times10^{-2}\pm7.83\times10^{-3}$**	$3.334\times10^{-2}\pm4.69\times10^{-3}$	$2.680\times10^{-2}\pm6.93\times10^{-3}$	$2.370\times10^{-2}\pm3.68\times10^{-3}$**	$2.709\times10^{-2}\pm8.79\times10^{-3}$	$2.807\times10^{-2}\pm3.48\times10^{-3}$	$3.038\times10^{-2}\pm5.36\times10^{-3}$	$2.780\times10^{-2}\pm1.078\times10^{-2}$	16
Benzoic acid	$3.884\times10^{-1}\pm4.97\times10^{-2}$*	$4.869\times10^{-1}\pm7.92\times10^{-2}$	$5.223\times10^{-1}\pm1.120\times10^{-2}$	$4.496\times10^{-1}\pm5.76\times10^{-2}$	$4.183\times10^{-1}\pm7.47\times10^{-2}$*	$4.282\times10^{-1}\pm7.17\times10^{-2}$	$5.598\times10^{-1}\pm5.56\times10^{-2}$	$5.025\times10^{-1}\pm6.79\times10^{-2}$	16
Pipecolic acid	$4.062\times10^{-1}\pm7.81\times10^{-2}$**	$5.544\times10^{-1}\pm1.47\times10^{-2}$	$5.063\times10^{-1}\pm5.54\times10^{-2}$	$5.018\times10^{-1}\pm1.057\times10^{-1}$	$3.894\times10^{-1}\pm4.88\times10^{-2}$**	$5.009\times10^{-1}\pm6.21\times10^{-2}$	$6.216\times10^{-1}\pm6.34\times10^{-2}$	$5.908\times10^{-1}\pm5.67\times10^{-2}$	16
Fumaric acid	$5.772\times10^{-2}\pm2.105\times10^{-2}$*	$8.42\times10^{-2}\pm1.64\times10^{-2}$	$7.562\times10^{-2}\pm1.537\times10^{-2}$	$5.405\times10^{-2}\pm8.78\times10^{-3}$**	$6.959\times10^{-2}\pm1.470\times10^{-2}$	$6.201\times10^{-2}\pm7.29\times10^{-3}$*	$8.21\times10^{-2}\pm1.91\times10^{-2}$	$7.327\times10^{-2}\pm1.464\times10^{-2}$	16
L-Methionine	$2.997\pm2.36\times10^{-1}$*	$3.432\pm3.21\times10^{-1}$	$3.261\pm3.56\times10^{-1}$	$3.041\pm2.45\times10^{-1}$*	$3.143\pm3.88\times10^{-1}$	$3.611\pm5.00\times10^{-1}$	$4.103\pm3.68\times10^{-1}$	$3.722\pm2.22\times10^{-1}$	16
Pyridoxamine	$1.845\times10^{-1}\pm2.51\times10^{-2}$**	$2.491\times10^{-1}\pm9.4\times10^{-3}$	$2.369\times10^{-1}\pm2.53\times10^{-2}$	$2.352\times10^{-1}\pm3.65\times10^{-2}$	$1.840\times10^{-1}\pm1.68\times10^{-2}$**	$2.325\times10^{-1}\pm2.56\times10^{-2}$	$3.083\times10^{-1}\pm4.65\times10^{-2}$*	$3.055\times10^{-1}\pm3.40\times10^{-2}$**	16

Pyridoxine	$2.665\times10^{-1}\pm4.71\times10^{-2}$*	$2.044\times10^{-1}\pm2.12\times10^{-2}$	$2.461\times10^{-1}\pm3.14\times10^{-2}$*	$2.687\times10^{-1}\pm3.75\times10^{-2}$**	$2.298\times10^{-1}\pm3.85\times10^{-2}$	$2.492\times10^{-1}\pm4.20\times10^{-2}$*	$2.832\times10^{-1}\pm4.70\times10^{-2}$**	$2.817\times10^{-1}\pm3.13\times10^{-2}$**	16
D-Xylose	$2.184\times10^{-2}\pm9.14\times10^{-3}$*	$3.321\times10^{-2}\pm5.13\times10^{-3}$	$2.329\times10^{-2}\pm1.184\times10^{-2}$	$1.742\times10^{-2}\pm1.148\times10^{-2}$*	$2.341\times10^{-2}\pm2.038\times10^{-2}$	$2.220\times10^{-2}\pm1.120\times10^{-2}$	$2.893\times10^{-2}\pm1.352\times10^{-2}$	$2.323\times10^{-2}\pm1.550\times10^{-2}$	16
Citric acid	$1.284\pm5.25\times10^{-1}$*	$1.845\pm1.87\times10^{-1}$	$1.619\pm2.63\times10^{-1}$	$1.238\pm1.86\times10^{-1}$**	$1.548\pm4.20\times10^{-1}$	$1.513\pm2.08\times10^{-1}$*	$1.797\pm2.67\times10^{-1}$	$1.620\pm3.49\times10^{-1}$	16
Pantothenic acid	$3.423\times10^{-1}\pm1.891\times10^{-1}$*	$5.563\times10^{-1}\pm9.57\times10^{-2}$	$4.853\times10^{-1}\pm1.659\times10^{-1}$	$3.450\times10^{-1}\pm3.80\times10^{-2}$**	$4.625\times10^{-1}\pm1.516\times10^{-1}$	$5.024\times10^{-1}\pm1.063\times10^{-1}$	$6.394\times10^{-1}\pm1.323\times10^{-1}$	$6.460\times10^{-1}\pm2.074\times10^{-1}$	16
Uridine	$7.255\times10^{-2}\pm1.679\times10^{-2}$**	$1.225\times10^{-1}\pm1.41\times10^{-2}$	$1.150\times10^{-1}\pm1.98\times10^{-2}$	$1.023\times10^{-1}\pm1.69\times10^{-2}$*	$1.180\times10^{-1}\pm1.98\times10^{-2}$	$1.155\times10^{-1}\pm2.43\times10^{-2}$	$1.293\times10^{-1}\pm2.77\times10^{-2}$	$1.266\times10^{-1}\pm1.42\times10^{-2}$	16
gamma-Glutamyl-leucine	$1.877\times10^{-1}\pm3.55\times10^{-2}$**	$3.247\times10^{-1}\pm7.16\times10^{-2}$	$3.199\times10^{-1}\pm1.343\times10^{-1}$	$2.113\times10^{-1}\pm1.146\times10^{-1}$	$2.070\times10^{-1}\pm1.050\times10^{-1}$*	$3.249\times10^{-1}\pm7.32\times10^{-2}$	$2.774\times10^{-1}\pm1.245\times10^{-1}$	$3.487\times10^{-1}\pm5.07\times10^{-2}$	16
Glutamylglutamine	$6.868\times10^{-1}\pm1.919\times10^{-1}$*	$9.11\times10^{-1}\pm1.22\times10^{-1}$	$8.69\times10^{-1}\pm1.87\times10^{-1}$	$6.335\times10^{-1}\pm1.575\times10^{-1}$**	$1.047\pm1.65\times10^{-1}$	$6.384\times10^{-1}\pm1.452\times10^{-1}$**	$8.92\times10^{-1}\pm2.71\times10^{-1}$	$9.08\times10^{-1}\pm3.25\times10^{-1}$	16
Cystine	$1.764\times10^{-2}\pm2.84\times10^{-3}$*	$2.505\times10^{-2}\pm7.06\times10^{-3}$	$1.565\times10^{-2}\pm2.93\times10^{-3}$*	$1.355\times10^{-2}\pm2.66\times10^{-3}$**	$2.392\times10^{-2}\pm6.07\times10^{-3}$	$1.787\times10^{-2}\pm3.56\times10^{-3}$	$2.182\times10^{-2}\pm6.77\times10^{-3}$	$1.882\times10^{-2}\pm4.13\times10^{-3}$	16
1-Methyladenosine	$8.83\times10^{-2}\pm7.2\times10^{-3}$*	$1.103\times10^{-1}\pm1.58\times10^{-2}$	$8.80\times10^{-2}\pm1.63\times10^{-2}$*	$1.015\times10^{-1}\pm1.03\times10^{-2}$	$9.59\times10^{-2}\pm1.43\times10^{-2}$	$1.016\times10^{-1}\pm2.15\times10^{-2}$	$1.116\times10^{-1}\pm1.53\times10^{-2}$	$1.100\times10^{-1}\pm1.76\times10^{-2}$	16
Hydroxypropionic acid	$2.998\times10^{-2}\pm8.97\times10^{-3}$*	$2.069\times10^{-2}\pm2.51\times10^{-3}$	$2.823\times10^{-2}\pm7.86\times10^{-3}$	$3.111\times10^{-2}\pm6.01\times10^{-3}$**	$3.540\times10^{-2}\pm1.249\times10^{-2}$*	$3.093\times10^{-2}\pm7.95\times10^{-3}$*	$3.410\times10^{-2}\pm5.50\times10^{-3}$**	$2.997\times10^{-2}\pm4.05\times10^{-3}$**	20

Glyceraldehyde	92.5±13.4**	65.14±9.69	86.2±13.5*	50.48±14.35	67.40±15.92	91.1±23.8*	89.5±14.8**	87.8±18.0*	20
L-Tyrosine	15.97±1.795**	19.95±1.239	21.13±2.85	17.68±1.49*	20.12±3.64	17.65±2.51	17.32±2.16*	22.06±3.14	20
L-Lactic acid	$1.759\times10^{-1}\pm3.83\times10^{-2}$**	$1.103\times10^{-1}\pm2.98\times10^{-2}$	$1.909\times10^{-1}\pm3.01\times10^{-2}$**	$9.35\times10^{-2}\pm1.69\times10^{-2}$	$1.528\times10^{-1}\pm6.03\times10^{-2}$	$2.116\times10^{-1}\pm7.12\times10^{-2}$**	$3.018\times10^{-1}\pm5.17\times10^{-2}$**	$2.488\times10^{-1}\pm6.24\times10^{-2}$**	20
N-Acetylglutamic acid	$7.025\times10^{-1}\pm9.64\times10^{-2}$*	$5.131\times10^{-1}\pm1.217\times10^{-1}$	$9.21\times10^{-1}\pm2.40\times10^{-1}$**	$4.108\times10^{-1}\pm6.09\times10^{-1}$	$8.07\times10^{-1}\pm3.46\times10^{-2}$	$7.881\times10^{-1}\pm1.104\times10^{-1}$**	$8.198\times10^{-1}\pm9.28\times10^{-3}$**	$7.294\times10^{-1}\pm1.963\times10^{-1}$*	20
L-Acetylcarnitine	4.736±1.195**	$1.669\pm9.14\times10^{-1}$	$3.817\pm7.31\times10^{-1}$**	9.18×10-1±1.15	2.283±1.387	5.818±1.923**	5.806±1.419**	3.319±1.696	20
Pantothenic acid	$2.982\times10^{-1}\pm4.50\times10^{-2}$**	$4.632\times10^{-1}\pm7.13\times10^{-2}$	$5.276\times10^{-1}\pm1.660\times10^{-1}$	$2.852\times10^{-1}\pm5.21\times10^{-2}$**	$3.084\times10^{-1}\pm6.87\times10^{-2}$**	$5.276\times10^{-1}\pm6.09\times10^{-2}$	$5.657\times10^{-1}\pm1.205\times10^{-1}$	$4.341\times10^{-1}\pm1.093\times10^{-1}$	20
Pentadecanoic acid	$2.013\times10^{-2}\pm2.134\times10^{-2}$**	$1.636\times10^{-1}\pm9.61\times10^{-2}$	$7.755\times10^{-3}\pm1.575\times10^{-2}$**	$6.257\times10^{-2}\pm5.768\times10^{-2}$	$5.504\times10^{-2}\pm7.202\times10^{-2}$	$3.132\times10^{-2}\pm2.752\times10^{-2}$**	$4.295\times10^{-2}\pm1.822\times10^{-2}$*	$4.527\times10^{-2}\pm6.435\times10^{-2}$*	20
gamma-Glutamyl-leucine	$1.275\times10^{-1}\pm1.45\times10^{-2}$**	$3.090\times10^{-1}\pm2.75\times10^{-2}$	$3.217\times10^{-1}\pm1.401\times10^{-1}$	$2.212\times10^{-1}\pm7.48\times10^{-2}$*	$3.812\times10^{-1}\pm2.097\times10^{-1}$	$3.337\times10^{-1}\pm5.15\times10^{-2}$	$3.868\times10^{-1}\pm7.19\times10^{-2}$	$3.540\times10^{-1}\pm1.221\times10^{-1}$	20
L-Cystine	$2.543\times10^{-1}\pm2.33\times10^{-2}$**	$1.696\times10^{-1}\pm2.71\times10^{-2}$	$2.021\times10^{-1}\pm1.40\times10^{-2}$*	$2.004\times10^{-1}\pm1.64\times10^{-2}$*	$2.076\times10^{-1}\pm2.97\times10^{-2}$*	$2.169\times10^{-1}\pm1.23\times10^{-2}$**	$2.177\times10^{-1}\pm1.61\times10^{-2}$**	$2.084\times10^{-1}\pm3.89\times10^{-2}$	20
Deoxycholic acid	$4.039\times10^{-2}\pm2.569\times10^{-2}$**	$1.573\times10^{-1}\pm5.14\times10^{-2}$	$6.257\times10^{-2}\pm1.581\times10^{-2}$**	$1.421\times10^{-1}\pm5.85\times10^{-2}$	$1.358\times10^{-2}\pm8.40\times10^{-2}$	$1.284\times10^{-1}\pm7.85\times10^{-2}$	$1.827\times10^{-1}\pm8.39\times10^{-2}$	$2.030\times10^{-1}\pm1.274\times10^{-1}$	20
gamma-Glutamyltyrosine	$1.214\times10^{-1}\pm8.0\times10^{-3}$**	$2.976\times10^{-1}\pm3.94\times10^{-2}$	$3.722\times10^{-1}\pm9.97\times10^{-2}$	$2.287\times10^{-1}\pm3.64\times10^{-2}$*	$3.241\times10^{-1}\pm1.539\times10^{-1}$	$2.202\times10^{-1}\pm4.00\times10^{-2}$**	$2.625\times10^{-1}\pm5.55\times10^{-2}$	$2.896\times10^{-1}\pm8.51\times10^{-2}$	20

Supplementary Table S10. Validation results of semi-quantitative analysis method of *in vivo* components.

Name	*m/z*_ t_R	Precision (RSD %)		Repeatability (RSD %)		Stability (RSD %)	
		t_R	Intensity	t_R	Intensity	t_R	Intensity
Sophocarpine	247.1777_2.66	0.47	5.4	0.01	4.3	0.45	13.0
Matrine	249.1949_2.58	0.67	3.5	1.38	6.6	0.76	8.2
Inermine	283.0610_21.44	0.02	4.5	0.02	6.4	0.06	7.0
Calycosin	285.0743_20.74	0.04	10.4	0.02	10.4	0.04	8.3
Calycosin-7-O-glucoside	447.1286_14.02	0.01	13.4	0.17	13.8	0.32	10.7
Astragaloside A	785.4676_23.95	0.11	13.3	1.15	12.5	0.07	13.2

Supplementary Table S11. Identification of the prototype compounds of SF-AM herb pair in different stages of HCC rats.

NO.	Name	Formula	Type	t_R (min)	Mass error (ppm)	Main product Ions (*m/z*, Da)
1	Caulophylline*	C12H16N2O	$[M+H]^+$	1.84	-4.0	205.1313; 146.0588; 58.0664
2	Cytisine	C11H14N2O	$[M+H]^+$	2.44	-0.8	191.1192; 162.0964; 148.0739; 146.0578; 120.0820
3	9α-Hydroxymatrine	C15H24N2O2	$[M+H]^+$	2.46	-0.6	265.1909; 247.1800; 168.1378; 150.1272; 148.1120; 112.0773; 98.0613
4	Anagyrine	C15H20N2O	$[M+H]^+$	2.47	-0.9	245.1645; 150.1270; 148.1139; 98.0971
5	Matrine*	C15H24N2O	$[M+H]^+$	2.58	-0.5	249.1949; 247.1803; 176.1064; 150.1254; 148.1095;
6	Sophocarpine*	C15H22N2O	$[M+H]^+$	2.66	0.6	247.1777; 245.1624; 179.1516; 150.1253; 148.1092; 136.1099; 96.0792
7	Oxysophocarpine*	C15H22N2O2	$[M+H]^+$	3.00	0.3	263.1741; 245.1636; 203.1172; 177.1384; 150.1259; 138.1263; 136.1109; 96.0794
8	Oxymatrine*	C15H24N2O2	$[M+H]^+$	3.04	-0.6	265.1913; 247.1799; 205.1324; 148.1118; 150.1274; 136.1113
9	Lamprolobine	C15H24N2O2	$[M+H]^+$	4.23	-0.8	265.1908; 247.1807; 150.1272; 152.1426; 138.1271; 84.9611
10	Baptifoline	C15H20N2O2	$[M+H]^+$	12.15	-0.9	261.1573; 243.1481; 193.1320; 160.0768; 96.0809
11	Calycosin-7-O-glucoside*	C22H22O10	$[M+H]^+$	14.02	-0.6	447.1286; 285.0750; 270.0517; 253.0486; 225.0541
12	Baicalin	C21H18O11	$[M-H]^-$	16.34	-1.0	445.1890; 269.0433; 113.0262
13	Ononin*	C22H22O9	$[M+H]^+$	18.45	-0.3	431.1333; 269.0785; 254.0556; 237.0533; 213.0898
14	Daidzein	C15H10O4	$[M+H]^+$	19.74	-0.6	255.0573; 233.7974; 199.0728; 181.0678; 151.0554; 137.0261; 128.0605
15	Trifolirhizin*	C22H22O10	$[M+H]^+$	20.10	-4.2	447.1282; 285.0746; 255.0642; 175.0385; 151.0384; 123.0439

16	Calycosin*	C16H12O5	$[M+H]^+$	20.74	0.5	285.0743; 270.0513; 253.0490; 225.0536; 213.0536; 197.0591; 137.0227
17	Inermine	C16H12O5	$[M-H]^-$	21.44	-0.8	283.0610; 268.0445; 240.0364; 211.0407; 195.0425; 61.9907
18	Isoastragaloside IV*	C41H68O14	$[M+H]^+$	22.10	-2.3	785.4675; 587.3914; 473.3611; 419.3286; 143.1056
19	Wogonoside	C22H20O11	$[M-H]^-$	17.11	-2.2	459.0979; 283.0606; 255.0661; 175.0231; 113.0523; 85.0321
20	Astragaloside A*	C41H68O14	$[M+H]^+$	23.95	1.9	785.4676; 473.3583; 455.3495; 437.3385; 419.3276; 297.2209; 143.1058
21	Formononetin*	C16H12O4	$[M+H]^+$	25.61	0.5	269.0798; 253.0490; 237.0540; 226.0618; 213.0903; 197.0593; 118.0415
22	Kurarinone*	C26H30O6	$[M+H]^+$	30.50	-1.0	439.2112; 303.1572; 297.0750; 179.03224
23	Vexibinol*	C25H28O6	$[M+H]^+$	36.10	2.6	425.1960; 289.1426; 283.0597; 165.0177

*: Identified by comparing with reference standards.

Supplementary Table S12. Identification of the metabolites of SF-AM herb pair in hepatitis-cirrhosis-HCC rats.

ID	Formula	Source	t_R (min)	Adduct type	Mass error (ppm)	Fragment
M1	C15H22N2O7P2	P4, P10	1.85	$[M+H]^+$	4.5	no MS/MS
M2	C5H9NO2	P9	1.87	$[M+H]^+$	2.6	116.0706; 70.0655
M3	C5H11NO2	P9	1.88	$[M+H]^+$	-0.6	118.0861; 58.0676
M4	C8H15NO2	P9	1.98	$[M+H]^+$	-1.0	158.1170; 140.1083; 112.1118; 98.0962; 82.0666; 71.0745; 58.0673
M5	C12H16N2O	P2	2.38	$[M+H]^+$	-3.5	205.1328; 146.0590; 133.0505; 91.0525; 85.0280; 58.0679

M6	C15H20N2O2	P4, P6, P7	2.45	$[M+H]^+$	-2.0	261.1370; 243.1531; 203.1178; 114.0675; 96.0822
M7	C10H19NO4	P9	2.47	$[M+H]^+$	-1.7	218.1384; 144.1022; 85.0292; 60.0834; 57.0364
M8	C15H24N2O2	P5, P6, P7, P10	2.47	$[M+H]^+$	-1.6	265.1909; 263.1799; 247.1800; 219.1861; 188.1441; 168.1378; 150.1272; 98.0613
M9	C10H12N2O	P1	2.47	$[M+H]^+$	-5.0	177.1022; 160.0756; 143.0727; 132.0287; 115.0535
M10	C15H20N2O	P6, P7, P10	2.47	$[M+H]^+$	-1.0	245.1645; 150.1270; 132.0655; 113.0368; 98.0971; 70.0661
M11	C14H18N2O5	P4, P10	2.54	$[M+H]^+$	-2.3	295.1286; 259.1030; 149.0235; 130.0868; 121.0290; 84.0817
M12	C15H24N2O	P4, P6, P8, P9, P10	2.60	$[M+H]^+$	-0.8	249.1960; 247.1793; 150.1273; 136.1119
M13	C29H48O6	P18, P20	2.66	$[M+H]^+$	2.7	493.3529; 247.1796
M14	C15H22N2O	P4, P5, P7, P8, P9, P10	2.67	$[M+H]^+$	0.2	247.1806; 245.1650; 179.1540; 150.1275; 148.1116; 136.1121; 96.0814
M15	C15H24N2O2	P5, P6, P7, P10	3.07	$[M+H]^+$	0.5	365.1913; 247.1799; 205.1324; 177.1375; 150.1274; 148.1118; 136.1113; 120.0807; 84.9597
M16	C14H18N2O6	P10	3.68	$[M+H]^+$	0.5	311.1237; 294.0951; 248.1026; 202.0865; 182.0849; 165.0546; 136.0759; 123.0434
M17	C6H11NO	P9	6.63	$[M+H]^+$	0.2	114.0917; 96.0815; 79.0556; 77.0400; 69.0713; 55.0571
M18	C15H22N2O2	P4, P5, P6, P8, P9, P10	11.30	$[M+H]^+$	-0.3	263.1755; 245.1617; 202.1191; 176.1072; 164.1045; 136.1103; 96.0798

M19	C15H20N2O2	P4, P5, P6	12.13	$[M+H]^+$	-0.9	261.1595; 243.1481; 193.1320; 160.0768; 96.0809
M20	C15H22N2O2	P7, P8, P9, P4, P6	12.94	$[M+H]^+$	0.4	263.1755; 245.1648; 192.1386; 178.1202; 148.1118
M21	C16H10O2	P11, P13, P16, P21	16.41	$[M+H]^+$	-1.7	235.0757; 207.0819; 192.0884; 178.0784; 152.0630
M22	C22H20O11	P11, P12, P13, P15, P16, P17, P19, P21	16.78	$[M+H]^+$	-0.5	461.1074; 285.0747; 270.0526; 253.0504; 225.0524
M23	C15H12O5	P11, P13, P14, P15, P16, P21	18.17	$[M+H]^+$	1.9	273.1666; 153.0128
M24	C32H50O10	P18, P20	18.69	$[M+H]^+$	1.0	595.3496; 470.2613; 345.1805; 285.1543
M25	C22H20O10	P11	19.12	$[M+H]^+$	0.0	445.1130; 269.0810; 254.0605
M26	C16H12O5	P11, P13, P14, P15, P21	20.78	$[M+H]^+$	1.7	285.0759; 253.0533; 171.9839; 151.0406; 144.0783; 123.0472
M27	C22H20O11	P11, P13, P15, P16, P21	20.82	$[M+H]^+$	-0.8	461.1075; 285.0752; 175.0386; 151.0391; 123.0443
M28	C27H38N2O15/C32H38O13	P6, P7, P22	22.55	$[M+H]^+$	4.5	631.2346; 431.1947; 313.0716; 179.0342
M29	C27H30O12	P13	22.69	$[M+H]^+$	-1.0	547.1806; 509.2642; 371.1488; 315.0850; 165.0178
M30	C22H22O10	P13, P16	24.45	$[M+H]^+$	-1.0	447.1283; 429.1232; 385.0923; 341.0674; 313.0724; 237.0766; 193.0505
M31	C22H20O9	P11, P13, P15, P16, P21	24.46	$[M+H]^+$	-0.9	429.1154; 385.0955; 341.0676; 237.0719; 193.0477; 149.0251

M32	C35H54O10	P18, P20	26.21	$[M+H]^+$	-0.1	635.3727; 459.3478; 405.3092; 300.0518; 193.1587
M33	C29H46O6	P18, P20	26.69	$[M+H]^+$	0.2	491.3367; 473.3190; 455.3114; 437.3028; 373.2375
M34	C21H34N2O7	P8, P9, P10	27.63	$[M+H]^+$	2.5	427.2443; 425.8876
M35	C32H38O10	P22	27.80	$[M+H]^+$	0.8	583.2544; 523.2442; 297.1233
M36	C32H40O11	P22	29.68	$[M+H]^+$	0.8	601.2644; 415.2773; 347.1242; 253.1329; 239.1169
M37	C31H36O12	P22, P23	30.46	$[M+H]^+$	-1.7	601.2275; 477.1022; 301.0704; 283.0607; 165.0181
M38	C32H38O12	P22	31.39	$[M+H]^+$	-1.1	615.2386; 491.1220; 315.0870; 297.0752; 179.0338
M39	C35H54O9	P18, P20	32.07	$[M+H]^+$	-1.2	619.1670; 443.3634; 425.3353; 383.3261
M40	C32H40O10	P22	35.00	$[M+H]^+$	0.1	585.2707; 345.1436; 253.1321; 238.1088
M41	C22H33N5O7S	P1	35.69	$[M+H]^+$	-0.2	512.2177; 495.1909; 439.1278; 369.1845; 313.1218; 181.0265
M42	C25H24O10	P13	37.62	$[M+H]^+$	2.2	485.1469; 467.2399; 185.0741
M43	C21H28N2O8	P4, P10	38.26	$[M+H]^+$	1.7	437.1935; 303.1198
M44	C27H42O4	P18, P20	42.58	$[M+H]^+$	0.2	431.3154; 395.2948; 367.2957; 369.1953; 177.1297; 145.0993
M45	C20H33N4O4	P8	43.92	$[M+H]^+$	-1.2	394.2550; 304.2608; 278.2885
M46	C6H8O6	P12, P19	2.48	$[M-H]^-$	-2.4	175.0251; 87.0087; 69.0365
M47	C6H12O3	P12, P19	9.98	$[M-H]^-$	5.0	131.0719; 85.0672; 69.0361
M48	C22H20O11	P11, P12, P13, P15, P16, P17, P19, P21	17.13	$[M-H]^-$	-0.5	459.0976; 283.0617; 268.0385; 239.0373; 113.0260; 85.0320

M49	C22H20O10	P12, P17, P19	20.13	$[M-H]^-$	-1.5	443.0984; 267.0678; 252.0451; 146.9685; 113.0215; 85.0305
M50	C17H13NO4	P12, P19	20.20	$[M-H]^-$	-0.5	294.0774; 273.0767; 180.9127; 159.0321; 114.9928; 96.9611; 79.9596
M51	C22H20O11	P11, P12, P13, P15, P16, P17, P19, P21	22.48	$[M-H]^-$	0.3	459.0919; 283.0620; 255.0662; 254.0583; 175.0258; 113.0255
M52	C22H22O10	P17, P19	23.44	$[M-H]^-$	-2.1	445.1267; 269.0850; 254.0600; 175.0211; 113.0243; 99.0117
M53	C20H18O9	P12, P19	27.70	$[M-H]^-$	0.8	401.0794; 357.0599; 313.0708; 269.0843; 225.0535; 181.0634; 121.0306

Supplementary Table S13. Semi-quantitative results of prototype components of SF-AM herb pair in different stages of HCC rats (Normalized data).

NO.	Name	Week 8	Week 12	Week 16	Week 20
1	Caulophylline	$6.755\times10^{-3}\pm3.248\times10^{-3}$	$8.79\times10^{-3}\pm3.00\times10^{-3}$	$5.157\times10^{-3}\pm1.399\times10^{-3}$	$6.109\times10^{-3}\pm1.291\times10^{-3}$
2	Cytisine	$1.549\times10^{-3}\pm2.75\times10^{-4}$	$2.257\times10^{-3}\pm8.27\times10^{-4}$	$1.950\times10^{-3}\pm5.02\times10^{-4}$	$1.417\times10^{-3}\pm2.57\times10^{-4}$
3	9α-Hydroxymatrine	$6.725\times10^{-3}\pm1.550\times10^{-3}$	$7.765\times10^{-3}\pm1.912\times10^{-3}$	$5.923\times10^{-3}\pm1.937\times10^{-3}$	$4.711\times10^{-3}\pm4.67\times10^{-4}$
4	Anagyrine	$1.028\times10^{-2}\pm2.03\times10^{-3}$	$8.47\times10^{-3}\pm1.44\times10^{-3}$	$7.175\times10^{-3}\pm2.282\times10^{-3}$	$5.948\times10^{-3}\pm8.45\times10^{-4}$
5	Matrine	$1.262\times10^{-1}\pm2.44\times10^{-2}$	$1.407\times10^{-1}\pm2.81\times10^{-2}$	$1.206\times10^{-1}\pm1.67\times10^{-2}$	$9.41\times10^{-2}\pm9.0\times10^{-3}$
6	Sophocarpine	$7.685\times10^{-2}\pm1.911\times10^{-2}$	$6.638\times10^{-2}\pm1.179\times10^{-2}$	$5.979\times10^{-2}\pm1.459\times10^{-2}$	$3.650\times10^{-2}\pm7.27\times10^{-3}$
7	Oxysophocarpine	$3.182\times10^{-3}\pm2.380\times10^{-3}$	$5.809\times10^{-3}\pm2.736\times10^{-3}$	$2.302\times10^{-3}\pm7.95\times10^{-4}$	$3.955\times10^{-3}\pm1.533\times10^{-3}$
8	Oxymatrine	$4.627\times10^{-3}\pm2.099\times10^{-3}$	$7.147\times10^{-3}\pm3.806\times10^{-3}$	$2.949\times10^{-3}\pm9.89\times10^{-4}$	$5.769\times10^{-3}\pm2.184\times10^{-3}$
9	Lamprolobine	$1.885\times10^{-3}\pm6.88\times10^{-4}$	$1.741\times10^{-3}\pm7.43\times10^{-4}$	$1.935\times10^{-3}\pm1.200\times10^{-3}$	$1.671\times10^{-3}\pm6.59\times10^{-4}$
10	Baptifoline	$6.971\times10^{-3}\pm1.047\times10^{-3}$	$1.248\times10^{-2}\pm4.09\times10^{-3}$	$1.008\times10^{-2}\pm1.97\times10^{-3}$	$5.517\times10^{-3}\pm2.233\times10^{-3}$
11	Calycosin 7-O-glucoside	$1.260\times10^{-3}\pm4.90\times10^{-4}$	$1.284\times10^{-3}\pm3.15\times10^{-4}$	$1.264\times10^{-3}\pm2.78\times10^{-4}$	$9.84\times10^{-4}\pm1.71\times10^{-4}$
12	Baicalin	$3.823\times10^{-2}\pm1.687\times10^{-2}$	$1.081\times10^{-2}\pm1.92\times10^{-3}$	$2.032\times10^{-2}\pm1.006\times10^{-2}$	$1.032\times10^{-2}\pm4.38\times10^{-3}$

13	Ononin	$1.787\times10^{-3}\pm5.\ 92\times10^{-4}$	$1.673\times10^{-3}\pm7.12\times10^{-4}$	$1.606\times10^{-3}\pm5.25\times10^{-4}$	$1.379\times10^{-3}\pm3.18\times10^{-4}$
14	Daidzein	$1.228\times10^{-2}\pm5.23\times10^{-3}$	$4.576\times10^{-3}\pm1.970\times10^{-3}$	$5.465\times10^{-3}\pm2.633\times10^{-3}$	$2.220\times10^{-3}\pm5.64\times10^{-4}$
15	Trifolirhizin	$7.227\times10^{-3}\pm2.803\times10^{-3}$	$5.755\times10^{-3}\pm2.651\times10^{-3}$	$5.070\times10^{-3}\pm4.849\times10^{-3}$	$4.528\times10^{-3}\pm2.177\times10^{-3}$
16	Calycosin	$2.756\times10^{-2}\pm1.009\times10^{-2}$	$3.641\times10^{-2}\pm9.08\times10^{-3}$	$3.841\times10^{-2}\pm1.149\times10^{-2}$	$2.399\times10^{-2}\pm5.81\times10^{-3}$
17	Inermine	$3.194\times10^{-3}\pm2.93\times10^{-4}$	$3.365\times10^{-3}\pm1.729\times10^{-3}$	$2.660\times10^{-3}\pm6.02\times10^{-4}$	$1.560\times10^{-3}\pm7.70\times10^{-4}$
18	Isoastragaloside IV	$1.839\times10^{-3}\pm7.89\times10^{-4}$	$1.523\times10^{-3}\pm2.91\times10^{-4}$	$1.476\times10^{-3}\pm6.37\times10^{-4}$	$1.601\times10^{-3}\pm8.27\times10^{-4}$
19	Wogonoside	$3.507\times10^{-1}\pm1.134\times10^{-1}$	$4.407\times10^{-1}\pm1.146\times10^{-1}$	$4.693\times10^{-1}\pm1.169\times10^{-1}$	$2.999\times10^{-1}\pm1.104\times10^{-1}$
20	Astragaloside A	$2.408\times10^{-3}\pm1.675\times10^{-3}$	$3.633\times10^{-3}\pm1.692\times10^{-3}$	$3.556\times10^{-3}\pm1.121\times10^{-3}$	$3.259\times10^{-3}\pm2.013\times10^{-3}$
21	Formononetin	$8.51\times10^{-3}\pm4.33\times10^{-3}$	$1.381\times10^{-3}\pm7.66\times10^{-3}$	$5.497\times10^{-3}\pm1.472\times10^{-3}$	$4.756\times10^{-3}\pm2.481\times10^{-3}$
22	Kurarinone	$2.773\times10^{-3}\pm1.063\times10^{-3}$	$2.367\times10^{-3}\pm1.376\times10^{-3}$	$3.164\times10^{-3}\pm1.194\times10^{-3}$	$2.349\times10^{-3}\pm8.18\times10^{-4}$
23	Vexibinol	$1.649\times10^{-3}\pm6.32\times10^{-4}$	$1.297\times10^{-3}\pm4.89\times10^{-4}$	$1.664\times10^{-3}\pm4.75\times10^{-4}$	$9.94\times10^{-4}\pm0.173\times10^{-4}$

Supplementary Table S14. Semi-quantitative results of metabolites of SF-AM herb pair in different stages of HCC rats.

ID	Week 8	Week 12	Week 16	Week 20
M1	$2.647\times10^{-2}\pm8.03\times10^{-3}$	$2.430\times10^{-2}\pm1.530\times10^{-2}$	$3.374\times10^{-2}\pm1.127\times10^{-2}$	$1.708\times10^{-2}\pm7.19\times10^{-3}$
M2	$2.345\times10^{-1}\pm2.01\times10^{-2}$	$2.622\times10^{-1}\pm7.57\times10^{-2}$	$3.912\times10^{-1}\pm5.35\times10^{-2}$	$2.168\times10^{-1}\pm2.40\times10^{-2}$
M3	$1.110\pm3.51\times10^{-1}$	$7.455\times10^{-1}\pm1.990\times10^{-1}$	$1.121\pm4.43\times10^{-1}$	$6.484\times10^{-1}\pm6.10\times10^{-2}$
M4	$2.541\times10^{-1}\pm9.34\times10^{-2}$	$4.242\times10^{-1}\pm1.873\times10^{-1}$	$3.656\times10^{-1}\pm1.049\times10^{-1}$	$1.741\times10^{-1}\pm8.90\times10^{-2}$
M5	$7.315\times10^{-2}\pm2.867\times10^{-2}$	$1.146\times10^{-1}\pm2.63\times10^{-2}$	$6.347\times10^{-2}\pm1.491\times10^{-2}$	$8.76\times10^{-2}\pm1.73\times10^{-2}$
M6	$3.005\times10^{-2}\pm8.68\times10^{-3}$	$3.830\times10^{-2}\pm8.09\times10^{-3}$	$2.876\times10^{-2}\pm1.013\times10^{-2}$	$2.660\times10^{-2}\pm0.381\times10^{-2}$
M7	$1.204\times10^{-1}\pm7.02\times10^{-2}$	$3.705\times10^{-2}\pm2.706\times10^{-2}$	$7.241\times10^{-2}\pm1.879\times10^{-2}$	$7.106\times10^{-2}\pm2.825\times10^{-2}$
M8	$8.01\times10^{-2}\pm2.72\times10^{-2}$	$1.163\times10^{-1}\pm4.73\times10^{-2}$	$7.303\times10^{-2}\pm2.190\times10^{-2}$	$8.71\times10^{-2}\pm2.44\times10^{-2}$
M9	$2.576\times10^{-2}\pm1.855\times10^{-2}$	$5.032\times10^{-3}\pm7.24\times10^{-4}$	$5.279\times10^{-3}\pm2.026\times10^{-3}$	$4.013\times10^{-3}\pm2.007\times10^{-3}$
M10	$1.162\times10^{-1}\pm2.75\times10^{-2}$	$1.140\times10^{-1}\pm2.37\times10^{-2}$	$8.97\times10^{-2}\pm2.97\times10^{-2}$	$8.54\times10^{-2}\pm1.15\times10^{-2}$
M11	$3.340\times10^{-2}\pm2.486\times10^{-2}$	$3.509\times10^{-2}\pm2.224\times10^{-2}$	$4.833\times10^{-2}\pm2.221\times10^{-2}$	$1.077\times10^{-1}\pm3.53\times10^{-2}$
M12	$1.411\pm2.18\times10^{-1}$	$1.900\pm5.02\times10^{-1}$	$1.487\pm1.63\times10^{-1}$	$1.359\pm1.86\times10^{-1}$

M13	$2.732\times10^{-1}\pm7.65\times10^{-2}$	$2.408\times10^{-1}\pm4.53\times10^{-2}$	$1.917\times10^{-1}\pm7.27\times10^{-2}$	$1.279\times10^{-1}\pm5.51\times10^{-2}$
M14	$8.54\times10^{-1}\pm1.42\times10^{-1}$	$8.94\times10^{-1}\pm1.84\times10^{-1}$	$7.431\times10^{-1}\pm1.914\times10^{-1}$	$5.294\times10^{-1}\pm1.335\times10^{-1}$
M15	$5.651\times10^{-2}\pm3.923\times10^{-2}$	$9.667\times10^{-2}\pm5.723\times10^{-2}$	$3.718\times10^{-2}\pm1.473\times10^{-2}$	$8.19\times10^{-2}\pm2.83\times10^{-2}$
M16	$2.656\times10^{-2}\pm4.47\times10^{-3}$	$2.825\times10^{-2}\pm4.87\times10^{-3}$	$2.292\times10^{-2}\pm6.17\times10^{-3}$	$1.533\times10^{-2}\pm2.62\times10^{-3}$
M17	$1.134\times10^{-1}\pm3.79\times10^{-2}$	$1.095\times10^{-1}\pm2.19\times10^{-2}$	$1.259\times10^{-1}\pm1.52\times10^{-2}$	$1.119\times10^{-1}\pm1.74\times10^{-2}$
M18	$1.334\times10^{-3}\pm2.79\times10^{-2}$	$3.145\times10^{-1}\pm1.199\times10^{-1}$	$2.899\times10^{-1}\pm5.55\times10^{-2}$	$2.139\times10^{-3}\pm9.40\times10^{-2}$
M19	$7.836\times10^{-2}\pm1.070\times10^{-2}$	$1.642\times10^{-1}\pm5.18\times10^{-2}$	$1.243\times10^{-1}\pm2.13\times10^{-2}$	$7.965\times10^{-2}\pm3.187\times10^{-2}$
M20	$5.249\times10^{-2}\pm7.16\times10^{-3}$	$1.288\times10^{-1}\pm4.87\times10^{-2}$	$1.359\times10^{-1}\pm2.28\times10^{-2}$	$8.52\times10^{-2}\pm3.43\times10^{-2}$
M21	$4.691\times10^{-2}\pm6.44\times10^{-3}$	$5.397\times10^{-2}\pm8.54\times10^{-3}$	$5.981\times10^{-2}\pm1.140\times10^{-2}$	$6.128\times10^{-2}\pm4.29\times10^{-3}$
M22	$1.284\times10^{-1}\pm4.42\times10^{-2}$	$1.421\times10^{-1}\pm2.80\times10^{-2}$	$1.089\times10^{-1}\pm3.17\times10^{-2}$	$7.808\times10^{-2}\pm2.937\times10^{-2}$
M23	$5.831\times10^{-3}\pm2.415\times10^{-3}$	$3.697\times10^{-2}\pm2.478\times10^{-2}$	$8.23\times10^{-3}\pm4.54\times10^{-3}$	$5.148\times10^{-3}\pm3.204\times10^{-3}$
M24	$3.877\times10^{-3}\pm1.045\times10^{-3}$	$1.431\times10^{-2}\pm1.083\times10^{-2}$	$1.196\times10^{-2}\pm9.91\times10^{-3}$	$7.049\times10^{-3}\pm2.227\times10^{-3}$
M25	$3.693\times10^{-2}\pm8.83\times10^{-2}$	$7.310\times10^{-2}\pm3.307\times10^{-2}$	$3.301\times10^{-2}\pm1.007\times10^{-2}$	$2.579\times10^{-2}\pm1.096\times10^{-2}$
M26	$2.756\times10^{-2}\pm1.009\times10^{-2}$	$3.641\times10^{-2}\pm9.08\times10^{-3}$	$3.841\times10^{-2}\pm1.149\times10^{-2}$	$2.399\times10^{-2}\pm5.81\times10^{-3}$
M27	$1.806\times10^{-1}\pm4.15\times10^{-2}$	$2.564\times10^{-1}\pm4.42\times10^{-2}$	$2.286\times10^{-1}\pm6.69\times10^{-2}$	$1.377\times10^{-1}\pm4.52\times10^{-2}$
M28	$2.095\times10^{-2}\pm1.621\times10^{-2}$	$2.735\times10^{-2}\pm1.984\times10^{-2}$	$9.26\times10^{-2}\pm1.179\times10^{-1}$	$5.605\times10^{-3}\pm2.945\times10^{-3}$
M29	$2.261\times10^{-2}\pm8.25\times10^{-3}$	$2.895\times10^{-2}\pm1.538\times10^{-2}$	$3.461\times10^{-2}\pm2.527\times10^{-2}$	$1.688\times10^{-2}\pm1.033\times10^{-2}$
M30	$3.540\times10^{-2}\pm2.455\times10^{-2}$	$3.391\times10^{-2}\pm1.216\times10^{-2}$	$2.728\times10^{-2}\pm2.223\times10^{-2}$	$1.348\times10^{-2}\pm3.76\times10^{-3}$
M31	$4.343\times10^{-2}\pm2.611\times10^{-2}$	$3.897\times10^{-2}\pm1.045\times10^{-2}$	$3.802\times10^{-2}\pm2.392\times10^{-2}$	$1.715\times10^{-2}\pm4.75\times10^{-3}$
M32	$1.508\times10^{-2}\pm6.63\times10^{-3}$	$2.327\times10^{-2}\pm1.056\times10^{-2}$	$3.754\times10^{-2}\pm3.803\times10^{-2}$	$9.14\times10^{-3}\pm1.188\times10^{-2}$
M33	$2.375\times10^{-2}\pm3.49\times10^{-3}$	$4.092\times10^{-2}\pm6.37\times10^{-3}$	$2.160\times10^{-2}\pm1.198\times10^{-2}$	$1.078\times10^{-2}\pm5.38\times10^{-3}$
M34	$4.540\times10^{-2}\pm3.019\times10^{-2}$	$5.650\times10^{-2}\pm3.223\times10^{-2}$	$4.202\times10^{-2}\pm1.873\times10^{-2}$	$2.074\times10^{-2}\pm1.253\times10^{-2}$
M35	$1.253\times10^{-2}\pm1.064\times10^{-2}$	$1.244\times10^{-2}\pm1.375\times10^{-3}$	$9.25\times10^{-3}\pm9.16\times10^{-3}$	$5.148\times10^{-3}\pm1.155\times10^{-3}$
M36	$2.270\times10^{-2}\pm5.03\times10^{-3}$	$2.639\times10^{-2}\pm1.862\times10^{-2}$	$3.677\times10^{-2}\pm3.384\times10^{-2}$	$1.582\times10^{-2}\pm1.655\times10^{-2}$
M37	$1.846\times10^{-2}\pm1.232\times10^{-2}$	$1.824\times10^{-2}\pm1.259\times10^{-2}$	$1.322\times10^{-2}\pm8.28\times10^{-3}$	$4.620\times10^{-3}\pm2.675\times10^{-3}$
M38	$3.771\times10^{-2}\pm1.704\times10^{-2}$	$4.368\times10^{-2}\pm2.209\times10^{-2}$	$4.046\times10^{-2}\pm2.601\times10^{-2}$	$3.129\times10^{-2}\pm2.195\times10^{-2}$

M39	$1.015\times10^{-2}\pm6.64\times10^{-3}$	$2.449\times10^{-2}\pm1.445\times10^{-2}$	$2.683\times10^{-2}\pm1.493\times10^{-2}$	$9.51\times10^{-3}\pm4.00\times10^{-3}$
M40	$9.71\times10^{-2}\pm2.43\times10^{-2}$	$6.537\times10^{-2}\pm3.740\times10^{-2}$	$7.213\times10^{-2}\pm6.188\times10^{-2}$	$5.139\times10^{-2}\pm3.732\times10^{-2}$
M41	$3.157\times10^{-3}\pm1.206\times10^{-3}$	$1.684\times10^{-2}\pm1.043\times10^{-2}$	$5.496\times10^{-3}\pm3.216\times10^{-3}$	$8.22\times10^{-3}\pm3.88\times10^{-3}$
M42	$1.357\times10^{-2}\pm1.413\times10^{-2}$	$4.832\times10^{-2}\pm2.839\times10^{-2}$	$3.079\times10^{-2}\pm1.656\times10^{-2}$	$1.983\times10^{-2}\pm1.085\times10^{-2}$
M43	$5.634\times10^{-2}\pm9.78\times10^{-3}$	$5.513\times10^{-2}\pm1.968\times10^{-2}$	$8.147\times10^{-2}\pm2.973\times10^{-2}$	$7.508\times10^{-2}\pm1.491\times10^{-2}$
M44	$9.34\times10^{-2}\pm9.6\times10^{-3}$	$7.830\times10^{-2}\pm2.613\times10^{-2}$	$8.14\times10^{-2}\pm2.25\times10^{-2}$	$5.244\times10^{-2}\pm2.127\times10^{-2}$
M45	$3.468\times10^{-2}\pm2.109\times10^{-2}$	$2.647\times10^{-2}\pm4.24\times10^{-3}$	$2.891\times10^{-2}\pm1.821\times10^{-2}$	$2.920\times10^{-2}\pm4.44\times10^{-3}$
M47	$1.068\times10^{-1}\pm4.28\times10^{-2}$	$1.063\times10^{-1}\pm7.77\times10^{-2}$	$2.314\times10^{-1}\pm1.218\times10^{-1}$	$1.995\times10^{-3}\pm6.26\times10^{-3}$
M46	$1.005\times10^{-1}\pm7.76\times10^{-2}$	$2.458\times10^{-1}\pm1.532\times10^{-1}$	$1.546\times10^{-1}\pm6.30\times10^{-2}$	$1.353\times10^{-2}\pm4.68\times10^{-3}$
M48	$1.087\pm5.07\times10^{-1}$	$1.065\pm2.14\times10^{-1}$	$9.502\times10^{-1}\pm3.613\times10^{-1}$	$6.649\times10^{-2}\pm2.765\times10^{-2}$
M49	$1.723\times10^{-1}\pm4.27\times10^{-2}$	$3.354\times10^{-1}\pm1.585\times10^{-1}$	$1.667\times10^{-1}\pm3.17\times10^{-2}$	$1.299\times10^{-2}\pm6.38\times10^{-3}$
M50	$5.87\times10^{-2}\pm3.63\times10^{-2}$	$1.223\times10^{-1}\pm1.306\times10^{-1}$	$8.79\times10^{-2}\pm6.08\times10^{-2}$	$1.656\times10^{-2}\pm5.11\times10^{-3}$
M51	3.520 ± 1.267	4.407 ± 1.046	4.693 ± 1.169	$3.380\times10^{-1}\pm6.57\times10^{-2}$
M52	$5.289\times10^{-1}\pm1.975\times10^{-1}$	$5.471\times10^{-1}\pm1.307\times10^{-1}$	$7.791\times10^{-1}\pm3.223\times10^{-1}$	$4.110\times10^{-2}\pm1.030\times10^{-2}$
M53	$1.115\times10^{-1}\pm1.50\times10^{-2}$	$1.214\times10^{-1}\pm3.59\times10^{-2}$	$1.041\times10^{-1}\pm2.06\times10^{-2}$	$1.104\times10^{-2}\pm1.35\times10^{-3}$

Supplementary Table S15. Details of binding energy between PI3K and active components.

Component	PI3K (4UWH)			
	H-bond	Salt bridge	Docking score (kcal/mol)	Glide energy (kcal/mol)
JXM	ILE685, ASP761	-	-7.645	-48.323
Astragaloside_A	LYS613, ASP761, ASP644, LYS636, GLN683	-	-9.909	-44.785
Vexibinol	LYS636, ILE685, GLN683	-	-9.225	-46.531

Kurarinone	SER614, LYS636, TLE685	-	-9.147	-48.109
Daidzein	GLN683, ILT685	-	-8.932	-34.879
Calycosin	ASP761, ILE685, GLN683	-	-8.531	-37.175
Formononetin	ILE685, LYS613	-	-7.983	-34.878
Ononin	ASP761, SER614, LYS636, ILE685	-	-7.43	-45.288
Calycosin-7-O-glucoside	GLN620, GLU692, SER687	-	-7.375	-39.6
Wogonoside	ASP761, LYS636	-	-7.155	-52.543
Inermine	ASP747	-	-6.603	-24.809
Baicalin	LYS613, LYS636	-	-6.164	-52.936
Oxysophocarpine	ASP747	-	-6.127	-27.064
Trifolirhizin	LYS636, ASP761, SER614	-	-6.084	-40.005
Isoastragaloside IV	GLU692, LYS636, ASP639, ASP644	-	-6.068	-19.47
Oxymatrine	-	-	-5.819	-24.776
Sophocarpine	-	-	-5.518	-20.748
Cytisine	-	-	-5.476	-23.329
Matrine	-	ASP761	-5.002	-19.221
Baptifoline	LYS636	-	-4.598	-28.124
Lamprolobine	-	-	-4.575	-22.635
Anagyrine	LYS636	-	-4.473	-26.436
9-alpha-Hydroxymatrine	SER614	ASP761	-3.999	-28.111
Caulophylline	LYS636	ASN748	-3.47	-21.466

Supplementary Table S16. Details of binding energy between Akt and active components.

Component	Akt (4GV1)			
	H-bond	Salt bridge	Docking score (kcal/mol)	Glide energy (kcal/mol)
0XZ	ASP292, ALA230, GLU228	ASP292, GLU234,	-8.523	-63.525
Isoastragaloside Ⅳ	GLU228, LYS179, PHE161	-	-9.091	-16.908
Baicalin	ASP292, GLU234	LYS276	-7.702	-57.175
Calycosin	ALA230, GLU228, GLU234	-	-7.373	-42.888
Wogonoside	LYS179, GLU234, ASN279	-	-6.789	-56.872
Formononetin	GLU228, ALA230	--	-6.647	-38.69
Astragaloside_A	LYS276	-	-6.439	-38.51
Vexibinol	GLU191, ASP292, LYS276, GLU234	-	-6.032	-50.423
Daidzein	ALA230	-	-5.306	-39.122
Kurarinone	GLU234		-5.195	-51.141
Caulophylline	-	ASP292, GLU234	-5.156	-29.462
Calycosin-7-O-glucoside	ASP274, LYS276, GLU234	-	-5.076	-51.476
Trifolirhizin	ASP292	-	-4.88	-49.284
Lamprolobine	-	ASP292, GLU234	-4.725	-34.331
Ononin	ASP274, LYS276, GLU191	-	-4.318	-47.935
Inermine	-	-	-4.121	-34.202
Baptifoline	-	ASP292, GLU234	-3.91	-31.659
Cytisine	GLU278	ASP292, GLU234	-3.881	-33.701
Anagyrine	-	ASP292, GLU234	-3.529	-29.93

Oxysophocarpine	-	ASP292, GLU234	-2.977	-26.361
Oxymatrine	-	ASP292, GLU234	-2.605	-24.794
Matrine	-	ASP292, GLU234	-2.42	-30.5
Sophocarpine	-	ASP292, GLU234	-2.177	-30.829
9-alpha-Hydroxymatrine	LYS276, ASN279	ASP292, GLU234	-2.06	-34.318

Supplementary Table S17. Details of binding energy between NF-κB p65 and active components.

Component	NF-κB p65 (6NV2)			
	H-bond	Salt bridge	Docking score (kcal/mol)	Glide energy (kcal/mol)
0V4	ASP215, ASN42, LYS122	-	-6.039	-43.566
Baicalin	GLY130	ARG132	-7.496	-38.549
Astragaloside_A	ASN42, LYS122	-	-7.444	-40.773
Wogonoside	ARG132, ARG131, GLY130	-	-6.434	-37.726
Calycosin-7-O-glucoside	SER45, LYS122, ASP215	-	-6.176	-40.729
Trifolirhizin	SER45	-	-5.928	-36.88
Vexibinol	ASP215, LYS122	-	-5.733	-40.494
Isoastragaloside	ASP215, ASN42, CSO38	-	-5.479	-31.504
Calycosin	ASP215, SER45	-	-5.233	-35.737
Ononin	GLU14, GLU39	-	-5.027	-38.168
Kurarinone	LYS122	-	-4.976	-37.382
9-alpha-Hydroxymatrine	LYS122	-	-4.897	-31.326

Daidzein	PHE119	-	-4.797	-28.066
Oxysophocarpine	-	-	-4.791	-26.872
Anagyrine	-	-	-4.781	-25.923
Sophocarpine	-	-	-4.773	-29.078
Inermine	LYS122	-	-4.738	-27.209
Baptifoline	LYS122	-	-4.529	-31.856
Cytisine	LYS122, ASN42	-	-4.32	-29.561
Caulophylline	-	-	-4.314	-23.317
Lamprolobine	-	-	-4.275	-24.418
Matrine	-	-	-4.083	-25.279
Oxymatrine	ARG132	-	-4.061	-27.477
Formononetin	ASP215, LYS122	-	-3.295	-33.348

Supplementary Table S18. Details of binding energy between Bcl-2 and active components.

Component	Bcl-2 (6O0K)			
	H-bond	Salt-bridge	Docking score (kcal/mol)	Glide energy (kcal/mol)
LBM	TYR202, ASN143, TRP144, GLY145	ASP111	-8.333	-68.405
Baicalin	ARG146, ASP140, GLU136	-	-6.905	-43.21
Daidzein	ASP103	ARG146	-6.3	-32.546
Wogonoside	ASN143, ARG146	-	-6.149	-43.208
Matrine	ASP103	ASP103	-5.844	-28.176
Isoastragaloside	ASP103, ASN143, ASP140, GLU136	-	-5.804	-46.375
Vexibinol	ARG146, ASP111	-	-5.727	-41.875

Cytisine	ASP103	ASP103	-5.533	-23.988
Astragaloside_A	GLU136, ASP140, ASN143, TYR108, GLN118	-	-5.429	-44.587
Inermine	-	-	-5.419	-28.848
Formononetin	GLU136	-	-5.363	-29.14
Kurarinone	ASP111, LEU137, ASP140	-	-5.305	-42.855
Calycosin	-	ARG146	-5.268	-30.821
Sophocarpine	ASP103	ASP103	-5.049	-28.632
Trifolirhizin	ASP140, GLU136	-	-5.026	-33.028
9-alpha-Hydroxymatrine	ASP103	ASP103	-4.938	-30.306
Anagyrine	ASP103	ASP103	-4.93	-26.085
Calycosin-7-O-glucoside	GLU136, LEU137, ASP140, ARG146	-	-4.89	-41.701
Caulophylline	-	ASP103	-4.877	-22.291
Baptifoline	ASP103, GLY145	-	-4.854	-27.899
Ononin	GLU136, ASP140, ARG146	-	-4.633	-39.055
Lamprolobine	ASP103	ASP103	-4.562	-27.591
Oxysophocarpine	-	ASP103	-3.197	-27.743
Oxymatrine	-	ASP103	-3.087	-26.242

frontiers
